# Supplementary material for: Associations between Prenatal Exposure to Phthalates and Features of the Metabolic Syndrome in Males from Childhood into Adulthood
Source: Int J Environ Res Public Health. 2022 Nov 18;19(22):15244. doi: 10.3390/ijerph192215244 (PMC9690816; doi:10.3390/ijerph192215244)
Supplement: Supplementary file 1 [file ijerph-19-15244-s001.zip › ijerph-1945131-supplementary.pdf]

Table S1. Phthalate metabolites (ng/mL) detected in maternal serum during pregnancy for males who had phthalates and any outcome available (N = 387).

		Phthalates and any outcome available *N=387*			
	LOD (ng/mL)	% > LOD	Min	Median	Max
MEP	0.65	85.79	0.00	3.29	128.39
MiBP	0.75	67.70	0.00	1.23	42.93
MnBP	0.61	96.38	0.00	2.81	210.63
MHBP	0.22	26.36	0.00	0.00	2.03
MBzP	0.26	34.11	0.00	0.00	73.77
MEHP	0.74	100.00	1.30	4.10	32.18
MECPP	0.25	98.97	0.00	1.01	10.84
MCMHP	0.39	98.45	0.00	1.46	17.07
MCPP	0.19	43.41	0.00	0.00	7.58
MINP	0.53	97.42	0.00	3.86	53.68
MCiOP	0.13	66.41	0.00	0.18	15.28
MiDP	0.72	17.05	0.00	0.00	9.80
∑MBP(i+n)			0.00	4.03	253.57
∑DEHPmetab			2.89	9.34	51.46
∑DiNPmetab			0.00	5.75	76.95
∑DEHPDiNPmetab			2.67	11.28	72.35
∑low MW phth.metab			0.00	8.14	241.12
∑high MW phth.metab			3.60	11.70	81.82
∑all phth.metab			7.57	23.98	354.43

Table S2. Outcomes (Median (IQR) or N(%)) of the singleton males in the Raine Study who were (n = 387) and were not (n = 1012) included in the present analysis.

	Not Included in Study (Maternal Serum Phthalate Levels or Outcome Data Unavailable) n=1012		Included in Study (Maternal Serum Phthalate Levels and Outcome Data Available) n=387		
	N	Median (IQR) or N(%)	N	Median (IQR) or N(%)	p-Value
**Age at follow-up (years)**					
Age 3	747	3.10 (3.05, 3.17)	365	3.10 (3.05, 3.16)	0.480
Age 5	755	5.92 (5.84, 6.06)	359	5.91 (5.85, 5.99)	0.331
Age 8	710	8.11 (7.83, 8.40)	353	8.10 (7.81, 8.33)	0.302
Age 10	692	10.56 (10.49, 10.69)	334	10.54 (10.48, 10.65)	0.060
Age 13	626	14.12 (14.04, 14.20)	298	14.13 (14.05, 14.20)	0.261
Age 16	545	17.02 (16.91, 17.13)	271	17.02 (16.91, 17.14)	0.485
Age 20	520	19.94 (19.66, 20.30)	237	20.02 (19.74, 20.39)	**0.041**
Age 22	388	22.07 (21.68, 22.43)	180	22.04 (21.74, 22.53)	0.487
**Alanine transaminase (U/L)**					
Age 13	464	17 (14, 22)	222	17 (14, 21)	0.157
Age 16	426	21 (16, 28)	208	21 (15, 29)	0.998
Age 20	415	30 (23, 42)	191	30 (21, 43)	0.845
Age 22	328	29 (23, 39.5)	148	28.5 (22, 40)	0.471
**Aspartate transaminase (U/L)**					
Age 16	425	26 (22, 31)	208	25 (21.5, 30)	0.164
Age 20	415	25 (21, 30)	191	27 (22, 32)	**0.013**
Age 22	328	26.2 (24, 32)	148	27 (23, 32)	0.831
**Gamma-glutamyl transferase (U/L)**					
Age 13	464	12 (10, 15)	222	12 (10, 13)	**0.025**
Age 16	426	15 (12, 19)	208	14 (11, 18)	0.461
Age 20	415	17 (14, 24)	191	17 (13, 23)	0.405
Age 22	328	19 (15, 26)	148	19 (14.75, 24.8)	0.574
**Dual energy X-ray absorptiometry (g)**					
Fat mass at age 20	411	15306 (10438, 22699)	195	14998 (11088, 22052)	0.889
Fat mass at age 27	300	19986 (14464, 28040)	151	20022 (14980, 27086)	0.865
Lean mass at age 20	411	56541 (52139, 61243)	195	56840 (51278, 62307)	0.741
Lean mass at age 27	300	58091 (53118, 63780)	151	59305 (53507, 65787)	0.248
Age at 20 year follow-up	411	18680 (18488, 18827)	195	18715 (18565, 18816)	**0.003**
Age at 27 year follow-up	300	21145 (20959, 21356)	152	21147 (20978, 21305)	0.626
**Uric acid (mmol/L)**					
Age 13	464	0.33 (0.28, 0.37)	222	0.32 (0.27, 0.36)	0.213
Age 20	415	0.34 (0.30, 0.39)	191	0.34 (0.30, 0.39)	0.859
Age 22	328	0.39 (0.34, 0.44)	148	0.38 (0.33, 0.44)	0.415
**Waist circumference (cm)**					
Age 13	525	73.10 (68.40, 81.00)	259	74.00 (69.10, 83.00)	0.180
Age 16	392	77.50 (73.70, 83.25)	201	78.05 (74.25, 86.25)	0.449
Age 20	448	80.73 (75.50, 87.63)	211	80.75 (75.00, 87.75)	0.949
Age 22	355	83.75 (77.35, 91.50)	157	84.00 (78.30, 91.40)	0.817
**Diastolic blood pressure (mm/Hg)**					
Age 3	418	53 (48, 58)	238	52 (47, 59)	0.687
Age 5	644	54 (50, 59)	327	55 (50, 60)	0.069
Age 8	627	56 (51.5, 60)	327	56 (51, 60)	0.764
Age 10	588	57 (52, 61)	298	57 (52, 61)	0.917
Age 13	521	58.2 (54.2, 63.0)	258	59.0 (53.6, 63.0)	0.613
Age 16	404	57.3 (53.6, 62.8)	204	58.6 (54.3, 62.4)	0.317
Age 20	450	64.5 (59.2, 70.0)	211	65.2 (60.8, 71.0)	0.234
Age 22	352	67.4 (62.4, 71.8)	157	65.4 (60.4, 70.4)	**0.023**
**Systolic blood pressure (mm/Hg)**					
Age 3	417	98 (93, 103)	238	100 (94, 105)	**0.011**
Age 5	644	103 (98, 108)	328	104 (100, 109)	**0.015**
Age 8	627	104 (98,111)	327	104 (99, 110)	0.663
Age 10	588	106 (100, 114)	298	106.5 (100, 112)	0.796
Age 13	521	113.4 (106.4, 120.6)	258	113.4 (107.4, 121.4)	0.557
Age 16	404	117.0 (111.6, 123.2)	204	117.7 (111.2, 123.4)	0.750
Age 20	450	120.4 (114.2, 130.2)	211	123.0 (114.6, 130.4)	0.259
Age 22	352	122.6 (115.8, 129.5)	157	122.0 (115.8, 130.4)	0.850
**Heart rate (BPM)**					

Age 5	333	88 (82, 95)	183	88 (81, 95)	0.746
Age 8	626	84 (76, 90)	328	82 (76,89)	0.179
Age 10	588	79 (72, 86)	297	79 (72, 85)	0.927
Age 13	521	78.2 (71.8, 85.6)	258	78.0 (72.6, 85.0)	0.817
Age 16	404	62.3 (56.8, 68.9)	204	63.6 (57.1, 68.6)	0.485
Age 20	450	74.7 (67.2, 81.6)	211	75.4 (66.6, 82.0)	0.904
Age 22	352	73.1 (64.9, 80.2)	155	70.8 (64.0, 79.2)	0.197
**Low density lipoprotein cholesterol (mmol/L)**					
Age 13	440	2.22 (1.83, 2.61)	209	2.20 (1.80, 2.60)	0.572
Age 16	418	2.16 (1.79, 2.56)	202	2.22 (1.88, 2.70)	0.147
Age 20	406	2.36 (1.98, 2.81)	187	2.39 (1.93, 2.79)	0.684
Age 22	326	2.71 (2.27, 3.22)	147	2.69 (2.38, 3.16)	0.991
**High density lipoprotein cholesterol (mmol/L)**					
Age 13	464	1.31 (1.15, 1.52)	222	1.32 (1.15, 1.55)	0.789
Age 16	426	1.18 (1.03, 1.35)	208	1.19 (1.05, 1.37)	0.444
Age 20	415	1.19 (1.03, 1.35)	191	1.22 (1.07, 1.42)	0.109
Age 22	328	1.20 (1.10, 1.40)	148	1.20 (1.10, 1.40)	0.854
**Triglycerides (mmol/L)**					
Age 13	440	0.85 (0.66, 1.12)	211	0.81 (0.65, 1.12)	0.640
Age 16	419	0.93 (0.72, 1.30)	203	0.90 (0.69, 1.23)	0.288
Age 20	409	0.97 (0.73, 1.34)	189	0.93 (0.73, 1.30)	0.410
Age 22	327	1.00 (0.80, 1.40)	147	1.00 (0.80, 1.40)	0.943
**Glucose (mmol/L)**					
Age 13	433	4.8 (4.6, 5.1)	209	4.9 (4.7, 5.1)	0.673
Age 16	411	4.8 (4.6, 5.1)	201	4.9 (4.6, 5.1)	0.484
Age 20	403	5.0 (4.8, 5.3)	188	5.0 (4.8, 5.3)	0.699
Age 22	321	5.1 (4.9, 5.3)	146	5.1 (4.9, 5.3)	0.763
**Insulin (mU/L)**					
Age 13	460	9.50 (6.89, 13.55)	219	9.60 (6.81, 13.90)	0.854
Age 16	423	7.08 (4.71, 10.50)	207	7.14 (4.50, 11.20)	0.461
Age 20	411	1.41 (1.41, 5.04)	190	1.41 (1.41, 4.28)	0.272
Age 22	325	6.2 (4.9, 8.6)	148	6.8 (5.2, 8.5)	0.232
**Creatinine (mU/L)**					
Age 13	464	60 (54, 68)	222	61 (56, 68)	0.232
Age 20	415	80.2 (73.7, 87.0)	191	79.7 (73.0, 86.1)	0.797
Age 22	328	82.0 (74.0, 88.0)	148	81.0 (74.0, 88.0)	0.614
**High Sensitivity C-Reactive Protein (mg/L)**					
Age 13	310	0.53 (0.29, 1.27)	144	0.56 (0.29, 1.63)	0.668
Age 16	417	0.43 (0.17, 0.98)	207	0.42 (0.19, 1.19)	0.472
Age 20	402	0.59 (0.25, 1.33)	179	0.68 (0.27, 1.39)	0.416
Age 22	321	0.73 (0.33, 1.42)	144	0.63 (0.39, 1.41)	0.661
**Homeostatic Model Assessment**					
Age 13	436	2.00 (1.47, 2.96)	209	2.04 (1.43, 3.03)	0.518
Age 16	416	1.50 (1.00, 2.24)	201	1.57 (0.92, 2.32)	0.681
Age 20	406	0.36 (0.31, 1.14)	188	0.33 (0.31, 1.02)	0.259
Age 22	324	1.41 (1.07, 2.03)	147	1.57 (1.18, 1.97)	0.283
**NAFLD at age 17**	386	42 (10.9)	186	21 (11.3)	0.883

Table S3. Adjusted Associations Between Categorized Phthalate Metabolite Levels and Systolic Blood Pressure (mm/Hg)

		3 year follow-up		5 year follow-up		8 year follow-up		10 year follow-up		14 year follow-up		17 year follow-up		20 year follow-up		22 year follow-up	
		Marginal Mean (95%CI)	p	Marginal Mean (95%CI)	p	Marginal Mean (95%CI)	p	Marginal Mean (95%CI)	p	Marginal Mean (95%CI)	p	Marginal Mean (95%CI)	p	Marginal Mean (95%CI)	p	Marginal Mean (95%CI)	p
MEP	<=1.750	100.07 (98.07, 102.06)		101.54 (99.77, 103.31)		104.16 (102.41, 105.91)		107.90 (106.14, 109.66)		113.51 (111.65, 115.37)		118.01 (115.99, 120.02)		122.28 (120.11, 124.46)		124.93 (122.47, 127.38)	
	>1.750 & <=5.714	100.69 (98.75, 102.62)	0.495	101.81 (100.09, 103.53)	0.738	104.16 (102.46, 105.85)	0.999	107.58 (105.89, 109.28)	0.679	112.75 (110.97, 114.53)	0.379	116.88 (114.96, 118.80)	0.262	120.78 (118.71, 122.84)	0.204	123.15 (120.82, 125.48)	0.182
	>5.714	101.58 (99.72, 103.44)	0.104	102.68 (101.08, 104.28)	0.166	105.01 (103.44, 106.58)	0.274	108.42 (106.85, 109.99)	0.507	113.56 (111.87, 115.24)	0.954	117.67 (115.82, 119.51)	0.740	121.54 (119.53, 123.55)	0.540	123.90 (121.6, 126.2)	0.449
MiBP	<=0.792	101.26 (99.30, 103.21)		102.51 (100.79, 104.24)		104.97 (103.26, 106.67)		108.51 (106.79, 110.22)		113.81 (111.99, 115.63)		118.04 (116.07, 120.01)		122.02 (119.88, 124.16)		124.45 (122.02, 126.88)	
	>0.792 & <=1.900	101.21 (99.32, 103.10)	0.957	102.27 (100.61, 103.93)	0.762	104.57 (102.93, 106.21)	0.607	107.94 (106.29, 109.58)	0.461	112.99 (111.24, 114.75)	0.354	117.02 (115.09, 118.94)	0.322	120.79 (118.68, 122.9)	0.313	123.07 (120.66, 125.48)	0.314
	>1.900	100.21 (98.30, 102.12)	0.250	101.63 (99.97, 103.29)	0.272	104.21 (102.58, 105.84)	0.319	107.90 (106.27, 109.52)	0.424	113.41 (111.69, 115.13)	0.639	117.81 (115.95, 119.67)	0.819	121.97 (119.98, 123.97)	0.967	124.53 (122.28, 126.77)	0.954
MnBP	<=1.999	101.54 (99.62, 103.46)		102.77 (101.07, 104.47)		105.20 (103.52, 106.88)		108.71 (107.02, 110.4)		113.99 (112.19, 115.79)		118.21 (116.25, 120.18)		122.20 (120.05, 124.35)		124.63 (122.18, 127.08)	
	>1.999 & <=4.099	101.02 (99.07, 102.97)	0.571	102.26 (100.55, 103.96)	0.519	104.68 (103.00, 106.36)	0.497	108.20 (106.52, 109.87)	0.500	113.48 (111.70, 115.25)	0.553	117.70 (115.78, 119.62)	0.613	121.69 (119.61, 123.76)	0.670	124.12 (121.77, 126.46)	0.705
	>4.099	100.24 (98.37, 102.12)	0.155	101.52 (99.89, 103.15)	0.119	103.99 (102.38, 105.59)	0.109	107.54 (105.94, 109.14)	0.125	112.88 (111.17, 114.59)	0.198	117.15 (115.29, 119.01)	0.294	121.19 (119.18, 123.19)	0.399	123.65 (121.38, 125.93)	0.469
MHBP	<=0.220	100.91 (99.23, 102.59)		102.19 (100.72, 103.65)		104.65 (103.19, 106.10)		108.20 (106.74, 109.66)		113.54 (112.02, 115.06)		117.82 (116.21, 119.42)		121.86 (120.19, 123.54)		124.34 (122.43, 126.26)	
	>0.220	100.73 (98.71, 102.75)	0.827	101.87 (100.09, 103.65)	0.673	104.23 (102.47, 105.99)	0.557	107.67 (105.90, 109.43)	0.453	112.84 (110.96, 114.72)	0.382	116.98 (114.92, 119.03)	0.368	120.88 (118.63, 123.13)	0.372	123.26 (120.73, 125.79)	0.380
MBzP	<=0.260	101.23 (99.54, 102.92)		102.42 (100.94, 103.90)		104.82 (103.35, 106.30)		108.31 (106.83, 109.79)		113.55 (112.01, 115.09)		117.73 (116.10, 119.36)		121.68 (119.98, 123.39)		124.09 (122.14, 126.05)	
	>0.260	100.13 (98.20, 102.06)	0.164	101.47 (99.79, 103.15)	0.169	103.99 (102.34, 105.64)	0.200	107.60 (105.95, 109.25)	0.281	113.03 (111.26, 114.79)	0.482	117.37 (115.43, 119.30)	0.673	121.47 (119.38, 123.57)	0.839	124.00 (121.63, 126.36)	0.933
MEHP	<=2.960	101.55 (99.64, 103.46)		102.63 (100.96, 104.30)		104.94 (103.30, 106.59)		108.32 (106.68, 109.97)		113.42 (111.68, 115.17)		117.50 (115.60, 119.40)		121.35 (119.30, 123.41)		123.70 (121.38, 126.02)	
	>2.960 & <=5.250	100.11 (98.20, 102.02)	0.116	101.50 (99.82, 103.17)	0.160	104.04 (102.38, 105.7)	0.239	107.69 (106.03, 109.35)	0.411	113.17 (111.40, 114.94)	0.770	117.57 (115.65, 119.48)	0.949	121.74 (119.67, 123.82)	0.743	124.32 (121.97, 126.67)	0.640
	>5.250	100.97 (99.04, 102.91)	0.530	102.21 (100.52, 103.91)	0.602	104.64 (102.97, 106.31)	0.692	108.16 (106.49, 109.83)	0.831	113.45 (111.68, 115.23)	0.972	117.69 (115.76, 119.62)	0.848	121.71 (119.61, 123.82)	0.763	124.18 (121.77, 126.58)	0.721
MECPP	<=0.785	100.89 (98.96, 102.81)		102.06 (100.40, 103.73)		104.44 (102.81, 106.08)		107.90 (106.26, 109.54)		113.10 (111.35, 114.85)		117.24 (115.33, 119.15)		121.16 (119.06, 123.25)		123.54 (121.15, 125.94)	
	>0.785 & <=1.241	101.10 (99.16, 103.04)	0.819	102.14 (100.43, 103.85)	0.923	104.42 (102.73, 106.11)	0.977	107.76 (106.07, 109.45)	0.855	112.79 (110.98, 114.59)	0.726	116.79 (114.83, 118.76)	0.663	120.56 (118.43, 122.7)	0.628	122.85 (120.45, 125.26)	0.613
	>1.241	100.67 (98.77, 102.57)	0.817	102.16 (100.48, 103.84)	0.903	104.78 (103.12, 106.44)	0.659	108.52 (106.87, 110.18)	0.420	114.11 (112.37, 115.85)	0.238	118.58 (116.71, 120.45)	0.177	122.83 (120.83, 124.83)	0.152	125.46 (123.20, 127.72)	0.144
MCMHP	<=1.153	101.44 (99.50, 103.37)		102.60 (100.91, 104.29)		104.99 (103.32, 106.65)		108.45 (106.79, 110.12)		113.66 (111.89, 115.44)		117.83 (115.89, 119.76)		121.76 (119.65, 123.86)		124.15 (121.76, 126.55)	
	>1.153 & <=1.810	100.27 (98.38, 102.16)	0.206	101.38 (99.72, 103.05)	0.132	103.73 (102.09, 105.37)	0.100	107.15 (105.50, 108.79)	0.090	112.29 (110.54, 114.05)	0.115	116.40 (114.48, 118.31)	0.161	120.27 (118.18, 122.35)	0.219	122.63 (120.26, 124.99)	0.261
	>1.810	101.03 (99.12, 102.95)	0.659	102.42 (100.75, 104.09)	0.822	104.98 (103.33, 106.62)	0.988	108.64 (107.00, 110.28)	0.804	114.13 (112.40, 115.86)	0.583	118.53 (116.66, 120.40)	0.480	122.70 (120.68, 124.71)	0.422	125.26 (122.98, 127.55)	0.398
MCPP	<=0.190	100.85 (99.10, 102.60)		102.08 (100.54, 103.61)		104.51 (102.98, 106.03)		108.02 (106.50, 109.55)		113.31 (111.72, 114.90)		117.54 (115.84, 119.24)		121.54 (119.75, 123.33)		123.98 (121.94, 126.02)	

	>0.190	100.90 (99.09, 102.71)	0.948	102.15 (100.56, 103.73)	0.918	104.59 (103.02, 106.16)	0.896	108.12 (106.55, 109.69)	0.875	113.43 (111.77, 115.10)	0.863	117.68 (115.89, 119.48)	0.863	121.70 (119.78, 123.62)	0.868	124.16 (121.98, 126.34)	0.872
MINP	<=2.363	100.30 (98.41, 102.18)	0.554	101.70 (100.08, 103.32)	0.598	104.26 (102.67, 105.85)	0.588	107.93 (106.34, 109.51)	0.517	113.42 (111.72, 115.12)	0.389	117.82 (115.95, 119.68)	0.320	121.98 (119.95, 124.00)	0.282	124.53 (122.24, 126.83)	
	>2.363 & <=5.491	101.12 (99.13, 103.11)	0.374	102.15 (100.38, 103.92)	0.580	104.43 (102.68, 106.18)	0.829	107.76 (106.01, 109.51)	0.829	112.79 (110.95, 114.63)	0.465	116.79 (114.81, 118.77)	0.310	120.55 (118.41, 122.68)	0.232	122.82 (120.40, 125.23)	0.201
	>5.491	101.21 (99.30, 103.11)	0.322	102.52 (100.83, 104.2)	0.311	105.01 (103.34, 106.68)	0.327	108.6 (106.93, 110.27)	0.382	113.97 (112.21, 115.74)	0.518	118.27 (116.37, 120.18)	0.646	122.34 (120.27, 124.40)	0.761	124.82 (122.47, 127.17)	0.827
MCiOP	<=0.130	100.73 (98.87, 102.59)		101.73 (100.13, 103.33)		103.99 (102.41, 105.56)		107.31 (105.74, 108.88)		112.32 (110.63, 114.00)		116.31 (114.46, 118.16)		120.05 (118.03, 122.08)		122.31 (119.98, 124.64)	
	>0.130	101.22 (99.47, 102.96)	0.545	102.56 (101.01, 104.10)	0.245	105.07 (103.53, 106.61)	0.106	108.70 (107.16, 110.24)	**0.038**	114.13 (112.54, 115.73)	**0.016**	118.48 (116.80, 120.17)	**0.013**	122.59 (120.84, 124.34)	**0.014**	125.10 (123.13, 127.08)	**0.016**
MiDP	<=0.720	100.68 (99.04, 102.32)		101.99 (100.56, 103.42)		104.48 (103.05, 105.91)		108.07 (106.64, 109.50)		113.46 (111.97, 114.94)		117.77 (116.21, 119.34)		121.86 (120.24, 123.48)		124.38 (122.52, 126.23)	
	>0.720	101.92 (99.56, 104.29)	0.217	102.77 (100.68, 104.87)	0.378	104.91 (102.87, 106.95)	0.610	108.09 (106.04, 110.13)	0.985	112.89 (110.68, 115.11)	0.555	116.72 (114.26, 119.18)	0.347	120.32 (117.58, 123.06)	0.241	122.48 (119.40, 125.55)	0.199
∑MBP(i+n)	<=2.860	101.49 (99.52, 103.46)		102.62 (100.87, 104.37)	0.149	104.97 (103.24, 106.7)		108.40 (106.67, 110.14)		113.57 (111.73, 115.41)		117.70 (115.70, 119.69)		121.57 (119.41, 123.74)		123.92 (121.46, 126.38)	
	>2.860 & <=5.986	101.43 (99.52, 103.34)	0.948	102.69 (101.03, 104.36)	0.926	105.15 (103.51, 106.79)	0.814	108.70 (107.06, 110.34)	0.695	114.04 (112.29, 115.78)	0.589	118.30 (116.40, 120.21)	0.549	122.33 (120.26, 124.39)	0.532	124.78 (122.43, 127.12)	0.527
	>5.986	99.99 (98.13, 101.85)	0.100	101.30 (99.69, 102.92)	0.101	103.80 (102.21, 105.39)	0.123	107.40 (105.81, 108.99)	0.188	112.80 (111.10, 114.50)	0.371	117.12 (115.27, 118.97)	0.567	121.20 (119.20, 123.19)	0.752	123.69 (121.42, 125.95)	0.861
∑DEHP	<=7.700	100.93 (98.99, 102.88)		102.07 (100.37, 103.78)		104.43 (102.75, 106.11)		107.86 (106.18, 109.54)		113.02 (111.23, 114.81)		117.15 (115.19, 119.10)		121.06 (118.94, 123.17)		123.46 (121.08, 125.85)	
	>7.700 & <=11.320	100.89 (99.00, 102.77)	0.959	102.00 (100.35, 103.65)	0.930	104.34 (102.71, 105.97)	0.908	107.75 (106.12, 109.38)	0.886	112.88 (111.14, 114.61)	0.871	116.98 (115.09, 118.87)	0.870	120.87 (118.83, 122.9)	0.873	123.25 (120.94, 125.56)	0.876
	>11.320	100.88 (98.94, 102.81)	0.952	102.33 (100.64, 104.03)	0.751	104.93 (103.26, 106.6)	0.516	108.64 (106.98, 110.31)	0.310	114.20 (112.44, 115.96)	0.173	118.66 (116.75, 120.56)	0.134	122.91 (120.85, 124.98)	0.121	125.56 (123.20, 127.92)	0.118
∑DiNP	<=3.700	100.54 (98.64, 102.44)		101.77 (100.14, 103.40)		104.19 (102.60, 105.79)		107.70 (106.11, 109.30)		112.97 (111.25, 114.68)		117.18 (115.30, 119.06)		121.17 (119.13, 123.20)		123.61 (121.30, 125.91)	
	>3.700 & <=8.292	100.98 (99.02, 102.93)	0.640	102.13 (100.39, 103.86)	0.662	104.49 (102.78, 106.20)	0.701	107.92 (106.22, 109.63)	0.771	113.09 (111.29, 114.89)	0.885	117.22 (115.28, 119.17)	0.966	121.12 (119.02, 123.23)	0.972	123.50 (121.11, 125.90)	0.940
	>8.292	101.25 (99.34, 103.16)	0.445	102.61 (100.91, 104.30)	0.304	105.13 (103.45, 106.81)	0.223	108.75 (107.07, 110.44)	0.171	114.18 (112.41, 115.96)	0.158	118.54 (116.61, 120.46)	0.178	122.66 (120.58, 124.74)	0.208	125.20 (122.84, 127.57)	0.231
∑DEHPDiNP	<=9.529	100.76 (98.84, 102.69)		101.90 (100.22, 103.57)		104.24 (102.60, 105.89)		107.65 (106.01, 109.29)		112.78 (111.02, 114.53)		116.88 (114.96, 118.8)		120.77 (118.69, 122.84)		123.15 (120.82, 125.49)	
	>9.529 & <=12.850	100.95 (99.02, 102.88)	0.841	102.11 (100.42, 103.81)	0.792	104.48 (102.80, 106.16)	0.757	107.92 (106.24, 109.60)	0.733	113.08 (111.29, 114.87)	0.730	117.21 (115.27, 119.16)	0.744	121.13 (119.02, 123.25)	0.762	123.54 (121.14, 125.94)	0.775
	>12.850	100.98 (99.07, 102.89)	0.814	102.42 (100.74, 104.10)	0.519	105.00 (103.34, 106.66)	0.323	108.68 (107.02, 110.35)	0.179	114.19 (112.45, 115.94)	0.099	118.62 (116.73, 120.5)	0.082	122.84 (120.81, 124.87)	0.079	125.46 (123.14, 127.78)	0.081
∑low MWphth	<=5.550	100.41 (98.42, 102.41)		101.73 (99.97, 103.50)		104.22 (102.48, 105.97)		107.82 (106.07, 109.57)		113.21 (111.35, 115.07)		117.53 (115.51, 119.55)		121.63 (119.43, 123.82)		124.14 (121.65, 126.63)	
	>5.550 & <=12.300	101.44 (99.51, 103.37)	0.260	102.61 (100.91, 104.31)	0.273	104.99 (103.31, 106.67)	0.314	108.45 (106.78, 110.12)	0.409	113.66 (111.88, 115.43)	0.609	117.82 (115.9, 119.74)	0.777	121.76 (119.67, 123.84)	0.916	124.15 (121.79, 126.51)	0.992
	>12.300	100.73 (98.87, 102.59)	0.729	101.97 (100.36, 103.57)	0.772	104.39 (102.81, 105.98)	0.823	107.91 (106.33, 109.49)	0.901	113.20 (111.52, 114.89)	0.992	117.44 (115.6, 119.27)	0.924	121.44 (119.46, 123.42)	0.877	123.89 (121.64, 126.14)	0.853
∑highMWphth	<=9.770	100.93 (99.01, 102.85)		102.07 (100.40, 103.74)		104.42 (102.78, 106.06)		107.83 (106.19, 109.46)		112.95 (111.20, 114.70)		117.04 (115.13, 118.95)		120.93 (118.87, 123.00)		123.32 (120.99, 125.64)	
	>9.770 & <=13.420	100.82 (98.90, 102.73)	0.900	101.92 (100.24, 103.61)	0.854	104.25 (102.58, 105.91)	0.820	107.62 (105.96, 109.29)	0.792	112.70 (110.93, 114.48)	0.779	116.77 (114.83, 118.7)	0.784	120.62 (118.50, 122.73)	0.795	122.98 (120.58, 125.38)	0.802
	>13.420	100.98 (99.06, 102.90)	0.959	102.48 (100.78, 104.18)	0.616	105.10 (103.42, 106.78)	0.373	108.83 (107.15, 110.51)	0.191	114.41 (112.64, 116.17)	0.090	118.88 (116.99, 120.77)	0.066	123.15 (121.12, 125.19)	0.059	125.82 (123.50, 128.14)	0.058

∑allphth	<=19.780	100.97 (98.96, 102.98)		102.10 (100.33, 103.87)		104.46 (102.71, 106.20)		107.88 (106.14, 109.63)		113.04 (111.18, 114.91)		117.16 (115.13, 119.2)		121.04 (118.82, 123.26)		123.41 (120.89, 125.92)	
	>19.780 & <=30.900	101.01 (99.10, 102.93)	0.962	102.27 (100.57, 103.98)	0.834	104.72 (103.03, 106.41)	0.729	108.26 (106.57, 109.95)	0.624	113.58 (111.80, 115.36)	0.540	117.83 (115.91, 119.75)	0.514	121.85 (119.78, 123.91)	0.508	124.30 (121.97, 126.64)	0.509
	>30.900	100.64 (98.78, 102.50)	0.726	101.96 (100.35, 103.57)	0.862	104.45 (102.88, 106.03)	0.999	108.05 (106.47, 109.62)	0.832	113.44 (111.76, 115.12)	0.651	117.75 (115.92, 119.58)	0.565	121.83 (119.85, 123.81)	0.516	124.34 (122.08, 126.59)	0.496

Models were adjusted for age at measurement, BMI z-score at measurement, maternal education and family income at birth, and length of breastfeeding.

Table S4. Adjusted Associations Between Categorized Phthalate Metabolite Levels and Diastolic Blood Pressure (mm/Hg)

		3 year followup		5 year followup		8 year followup		10 year followup		14 year followup		17 year followup		20 year followup		22 year followup	
		Marginal Mean (95%CI)	p	Marginal Mean (95%CI)	p	Marginal Mean (95%CI)	p	Marginal Mean (95%CI)	p	Marginal Mean (95%CI)	p	Marginal Mean (95%CI)	p	Marginal Mean (95%CI)	p	Marginal Mean (95%CI)	p
MEP	<=1.750	53.17 (51.41, 54.93)		54.91 (53.35, 56.47)		55.94 (54.42, 57.45)		56.98 (55.50, 58.45)		58.67 (57.18, 60.16)		60.89 (59.34, 62.45)		64.60 (62.97, 66.23)		68.43 (66.60, 70.26)	
	>1.750 & <=5.714	53.61 (51.89, 55.33)	0.614	55.11 (53.59, 56.63)	0.786	55.96 (54.49, 57.43)	0.969	56.79 (55.37, 58.22)	0.779	58.20 (56.77, 59.63)	0.468	60.18 (58.70, 61.66)	0.316	63.63 (62.09, 65.18)	0.239	67.29 (65.56, 69.02)	0.211
	>5.714	55.06 (53.41, 56.71)	**0.032**	56.29 (54.87, 57.72)	0.071	56.94 (55.57, 58.31)	0.154	57.53 (56.20, 58.85)	0.401	58.59 (57.25, 59.93)	0.908	60.29 (58.88, 61.70)	0.407	63.45 (61.97, 64.94)	0.172	66.9 (65.21, 68.59)	0.102
MiBP	<=0.792	54.07 (52.33, 55.80)		55.52 (54.00, 57.05)		56.34 (54.86, 57.82)		57.13 (55.69, 58.57)		58.46 (57.01, 59.92)		60.38 (58.86, 61.89)		63.76 (62.16, 65.36)		67.36 (65.56, 69.16)	
	>0.792 & <=1.900	53.64 (51.96, 55.33)	0.626	55.13 (53.65, 56.60)	0.601	55.97 (54.54, 57.39)	0.589	56.78 (55.40, 58.16)	0.592	58.15 (56.76, 59.55)	0.638	60.10 (58.62, 61.57)	0.702	63.51 (61.94, 65.08)	0.769	67.13 (65.35, 68.91)	0.812
	>1.900	54.19 (52.49, 55.89)	0.887	55.73 (54.26, 57.21)	0.782	56.62 (55.20, 58.03)	0.689	57.48 (56.12, 58.85)	0.582	58.92 (57.55, 60.30)	0.473	60.93 (59.50, 62.35)	0.433	64.40 (62.92, 65.88)	0.427	68.07 (66.41, 69.73)	0.433
MnBP	<=1.999	53.09 (51.38, 54.81)		54.64 (53.13, 56.15)		55.53 (54.07, 57.00)		56.41 (54.99, 57.83)		57.87 (56.44, 59.31)		59.90 (58.39, 61.41)		63.41 (61.81, 65.01)		67.09 (65.28, 68.91)	
	>1.999 & <=4.099	54.29 (52.57, 56.02)	0.167	55.73 (54.22, 57.24)	0.149	56.53 (55.08, 57.99)	0.145	57.31 (55.90, 58.72)	0.161	58.63 (57.22, 60.05)	0.242	60.54 (59.06, 62.02)	0.370	63.93 (62.38, 65.48)	0.529	67.53 (65.79, 69.27)	0.637
	>4.099	54.41 (52.74, 56.08)	0.130	55.89 (54.43, 57.34)	0.100	56.72 (55.32, 58.11)	0.086	57.53 (56.17, 58.88)	0.083	58.89 (57.53, 60.26)	0.117	60.84 (59.41, 62.27)	0.190	64.26 (62.77, 65.76)	0.302	67.89 (66.21, 69.58)	0.389
MHBP	<=0.220	53.84 (52.39, 55.3)		55.41 (54.14, 56.68)		56.30 (55.05, 57.56)		57.18 (55.95, 58.4)		58.63 (57.40, 59.86)		60.65 (59.38, 61.91)		64.15 (62.86, 65.43)		67.84 (66.38, 69.30)	
	>0.220	54.22 (52.41, 56.02)	0.643	55.57 (53.99, 57.16)	0.818	56.30 (54.77, 57.84)	0.999	56.99 (55.50, 58.47)	0.751	58.17 (56.67, 59.67)	0.448	59.97 (58.39, 61.54)	0.303	63.24 (61.58, 64.90)	0.231	66.77 (64.91, 68.63)	0.205
MBzP	<=0.260	53.88 (52.40, 55.35)		55.38 (54.08, 56.67)		56.22 (54.95, 57.50)		57.05 (55.80, 58.30)		58.44 (57.19, 59.69)		60.40 (59.11, 61.69)		63.84 (62.53, 65.15)		67.48 (65.99, 68.97)	
	>0.260	54.18 (52.47, 55.88)	0.689	55.67 (54.18, 57.16)	0.651	56.51 (55.08, 57.95)	0.626	57.33 (55.94, 58.73)	0.608	58.71 (57.30, 60.13)	0.622	60.67 (59.18, 62.16)	0.664	64.1 (62.53, 65.67)	0.715	67.74 (65.98, 69.5)	0.749
MEHP	<=2.960	54.08 (52.38, 55.78)		55.53 (54.04, 57.01)		56.33 (54.90, 57.77)		57.11 (55.73, 58.50)		58.44 (57.05, 59.84)		60.35 (58.90, 61.81)		63.74 (62.22, 65.26)		67.34 (65.63, 69.05)	
	>2.960 & <=5.250	53.12 (51.43, 54.81)	0.270	54.79 (53.30, 56.27)	0.331	55.76 (54.32, 57.20)	0.413	56.74 (55.34, 58.14)	0.569	58.35 (56.94, 59.76)	0.888	60.50 (59.02, 61.97)	0.844	64.12 (62.58, 65.66)	0.644	67.89 (66.15, 69.62)	0.548
	>5.250	54.79 (53.08, 56.50)	0.416	56.15 (54.65, 57.65)	0.409	56.90 (55.45, 58.35)	0.416	57.60 (56.19, 59.01)	0.450	58.82 (57.40, 60.25)	0.555	60.65 (59.16, 62.14)	0.679	63.95 (62.37, 65.52)	0.803	67.48 (65.69, 69.27)	0.877
MECPP	<=0.785	54.72 (53.02, 56.42)		56.23 (54.76, 57.7)		57.09 (55.67, 58.51)		57.92 (56.54, 59.30)		59.31 (57.92, 60.70)		61.28 (59.81, 62.74)		64.73 (63.17, 66.28)		68.38 (66.61, 70.16)	
	>0.785 & <=1.241	53.61 (51.89, 55.32)	0.205	55.02 (53.51, 56.52)	0.111	55.79 (54.33, 57.25)	0.062	56.53 (55.11, 57.95)	**0.033**	57.79 (56.36, 59.23)	**0.021**	59.65 (58.14, 61.16)	**0.026**	62.99 (61.40, 64.57)	**0.040**	66.57 (64.79, 68.34)	0.055
	>1.241	53.49 (51.80, 55.17)	0.159	55.06 (53.57, 56.54)	0.123	55.96 (54.53, 57.40)	0.104	56.85 (55.46, 58.24)	0.097	58.32 (56.93, 59.7)	0.123	60.35 (58.91, 61.78)	0.187	63.86 (62.37, 65.35)	0.287	67.57 (65.89, 69.24)	0.367
MCMHP	<=1.153	54.10 (52.38, 55.82)		55.69 (54.19, 57.19)		56.60 (55.15, 58.05)		57.50 (56.09, 58.91)		58.99 (57.56, 60.42)		61.03 (59.53, 62.53)		64.56 (62.98, 66.15)		68.27 (66.48, 70.06)	
	>1.153 & <=1.810	53.75 (52.06, 55.44)	0.691	55.17 (53.68, 56.65)	0.494	55.95 (54.52, 57.38)	0.348	56.69 (55.31, 58.08)	0.214	57.96 (56.57, 59.36)	0.120	59.83 (58.36, 61.29)	0.097	63.17 (61.63, 64.72)	0.098	66.75 (65.00, 68.49)	0.106
	>1.810	54.18 (52.47, 55.88)	0.927	55.68 (54.19, 57.17)	0.993	56.53 (55.09, 57.96)	0.915	57.35 (55.96, 58.74)	0.813	58.73 (57.33, 60.12)	0.684	60.68 (59.24, 62.13)	0.616	64.11 (62.61, 65.62)	0.582	67.76 (66.06, 69.45)	0.571
MCPP	<=0.190	53.70 (52.17, 55.22)		55.25 (53.91, 56.59)		56.14 (54.83, 57.45)		57.01 (55.73, 58.30)		58.47 (57.17, 59.76)		60.48 (59.15, 61.81)		63.98 (62.61, 65.34)		67.66 (66.12, 69.20)	

	>0.190	54.32 (52.73, 55.92)	0.384	55.75 (54.36, 57.15)	0.425	56.54 (55.19, 57.9)	0.482	57.30 (55.98, 58.63)	0.589	58.59 (57.26, 59.93)	0.811	60.48 (59.09, 61.86)	0.993	63.83 (62.39, 65.28)	0.834	67.42 (65.79, 69.05)	0.752
MINP	<=2.363	54.29 (52.62, 55.97)		55.74 (54.29, 57.18)		56.54 (55.16, 57.92)		57.31 (55.97, 58.64)		58.62 (57.27, 59.98)		60.53 (59.10, 61.96)		63.91 (62.4, 65.41)		67.51 (65.81, 69.20)	
	>2.363 & <=5.491	53.23 (51.47, 54.99)	0.228	54.77 (53.20, 56.33)	0.207	55.64 (54.13, 57.16)	0.201	56.50 (55.03, 57.97)	0.215	57.94 (56.47, 59.41)	0.296	59.94 (58.42, 61.47)	0.419	63.43 (61.83, 65.03)	0.566	67.11 (65.31, 68.90)	0.665
	>5.491	54.18 (52.48, 55.87)	0.892	55.68 (54.19, 57.17)	0.942	56.53 (55.08, 57.98)	0.993	57.36 (55.95, 58.77)	0.937	58.75 (57.34, 60.17)	0.840	60.72 (59.25, 62.20)	0.781	64.17 (62.63, 65.72)	0.745	67.82 (66.07, 69.57)	0.731
MCiOP	<=0.130	54.35 (52.70, 56.01)		55.78 (54.35, 57.20)		56.57 (55.19, 57.94)		57.32 (55.99, 58.65)		58.62 (57.27, 59.97)		60.50 (59.08, 61.92)		63.85 (62.34, 65.37)		67.43 (65.70, 69.17)	
	>0.130	53.73 (52.21, 55.25)	0.414	55.26 (53.91, 56.61)	0.438	56.13 (54.81, 57.46)	0.477	56.99 (55.69, 58.29)	0.556	58.42 (57.11, 59.73)	0.730	60.42 (59.07, 61.76)	0.895	63.89 (62.53, 65.25)	0.962	67.55 (66.03, 69.07)	0.884
MiDP	<=0.720	53.90 (52.49, 55.31)		55.44 (54.20, 56.68)		56.31 (55.09, 57.54)		57.16 (55.96, 58.37)		58.59 (57.38, 59.80)		60.58 (59.34, 61.82)		64.06 (62.81, 65.31)		67.73 (66.31, 69.15)	
	>0.720	54.11 (51.97, 56.24)	0.829	55.43 (53.56, 57.31)	0.996	56.15 (54.36, 57.93)	0.827	56.81 (55.09, 58.53)	0.617	57.97 (56.22, 59.71)	0.388	59.74 (57.88, 61.59)	0.288	62.99 (60.98, 64.99)	0.242	66.50 (64.27, 68.73)	0.228
∑MBP(i+n)	<=2.860	53.28 (51.52, 55.03)		54.74 (53.19, 56.29)		55.57 (54.06, 57.07)		56.36 (54.91, 57.82)		57.72 (56.25, 59.19)		59.65 (58.12, 61.18)		63.05 (61.44, 64.67)		66.66 (64.84, 68.49)	
	>2.860 & <=5.986	54.25 (52.56, 55.95)	0.261	55.77 (54.29, 57.25)	0.172	56.64 (55.21, 58.07)	0.119	57.48 (56.10, 58.87)	0.081	58.90 (57.51, 60.30)	0.067	60.89 (59.43, 62.35)	0.083	64.35 (62.82, 65.88)	0.118	68.00 (66.27, 69.73)	0.149
	>5.986	54.11 (52.45, 55.77)	0.335	55.61 (54.16, 57.05)	0.254	56.45 (55.06, 57.84)	0.201	57.27 (55.92, 58.62)	0.160	58.66 (57.30, 60.01)	0.149	60.61 (59.19, 62.03)	0.178	64.04 (62.56, 65.53)	0.230	67.67 (66.00, 69.34)	0.273
∑DEHP	<=7.700	54.37 (52.64, 56.09)		55.77 (54.26, 57.28)		56.53 (55.08, 57.99)		57.26 (55.85, 58.68)		58.51 (57.08, 59.94)		60.36 (58.86, 61.86)		63.69 (62.11, 65.27)		67.26 (65.50, 69.03)	
	>7.700 & <=11.320	53.16 (51.49, 54.84)	0.168	54.79 (53.32, 56.25)	0.196	55.73 (54.31, 57.14)	0.242	56.65 (55.28, 58.02)	0.345	58.18 (56.80, 59.56)	0.612	60.26 (58.82, 61.7)	0.893	63.83 (62.33, 65.34)	0.863	67.58 (65.88, 69.27)	0.734
	>11.320	54.48 (52.77, 56.19)	0.893	55.96 (54.46, 57.46)	0.798	56.79 (55.34, 58.24)	0.715	57.59 (56.18, 58.99)	0.619	58.93 (57.52, 60.35)	0.520	60.86 (59.39, 62.34)	0.485	64.27 (62.72, 65.83)	0.481	67.91 (66.14, 69.67)	0.487
∑DiNP	<=3.700	54.43 (52.74, 56.11)		55.76 (54.31, 57.21)		56.48 (55.09, 57.87)		57.14 (55.80, 58.49)		58.31 (56.94, 59.67)		60.09 (58.65, 61.53)		63.37 (61.86, 64.88)		66.92 (65.22, 68.61)	
	>3.700 & <=8.292	53.16 (51.43, 54.90)	0.151	54.85 (53.32, 56.38)	0.232	55.84 (54.35, 57.32)	0.356	56.82 (55.38, 58.26)	0.614	58.42 (56.98, 59.86)	0.858	60.58 (59.08, 62.08)	0.498	64.24 (62.66, 65.81)	0.296	68.05 (66.27, 69.83)	0.220
	>8.292	54.37 (52.67, 56.07)	0.951	55.84 (54.33, 57.35)	0.918	56.66 (55.20, 58.12)	0.795	57.44 (56.02, 58.86)	0.644	58.77 (57.35, 60.20)	0.473	60.70 (59.22, 62.19)	0.396	64.12 (62.56, 65.68)	0.362	67.77 (66.01, 69.53)	0.354
∑DEHPDiNP	<=9.529	54.10 (52.39, 55.81)		55.51 (54.02, 56.99)		56.28 (54.85, 57.71)		57.01 (55.63, 58.39)		58.27 (56.87, 59.66)		60.12 (58.65, 61.59)		63.46 (61.92, 65.00)		67.04 (65.32, 68.76)	
	>9.529 & <=12.850	53.45 (51.73, 55.17)	0.459	55.13 (53.62, 56.64)	0.619	56.11 (54.65, 57.57)	0.805	57.08 (55.66, 58.50)	0.912	58.68 (57.25, 60.11)	0.529	60.82 (59.32, 62.32)	0.335	64.46 (62.88, 66.04)	0.236	68.24 (66.46, 70.02)	0.200
	>12.850	54.41 (52.72, 56.11)	0.723	55.85 (54.35, 57.34)	0.658	56.64 (55.19, 58.08)	0.606	57.39 (55.99, 58.79)	0.555	58.68 (57.28, 60.08)	0.521	60.57 (59.11, 62.02)	0.531	63.93 (62.41, 65.46)	0.562	67.53 (65.80, 69.26)	0.587
∑low MWphth	<=5.550	53.58 (51.82, 55.34)		55.15 (53.60, 56.71)		56.05 (54.54, 57.57)		56.94 (55.46, 58.41)		58.41 (56.92, 59.9)		60.45 (58.89, 62.01)		63.97 (62.33, 65.62)		67.69 (65.84, 69.53)	
	>5.550 & <=12.300	53.59 (51.88, 55.31)	0.990	55.25 (53.74, 56.76)	0.901	56.21 (54.76, 57.67)	0.819	57.17 (55.76, 58.58)	0.719	58.75 (57.33, 60.16)	0.606	60.87 (59.40, 62.34)	0.556	64.49 (62.95, 66.03)	0.537	68.26 (66.53, 70.00)	0.535
	>12.300	54.66 (53.01, 56.31)	0.218	55.94 (54.51, 57.37)	0.301	56.62 (55.24, 57.99)	0.416	57.24 (55.91, 58.57)	0.639	58.35 (57.01, 59.69)	0.925	60.08 (58.68, 61.48)	0.608	63.29 (61.83, 64.76)	0.409	66.78 (65.13, 68.44)	0.326
∑highMWphth	<=9.770	53.88 (52.18, 55.59)		55.36 (53.87, 56.84)		56.18 (54.76, 57.60)		56.97 (55.60, 58.35)		58.32 (56.93, 59.71)		60.24 (58.78, 61.71)		63.65 (62.12, 65.19)		67.28 (65.56, 69.00)	
	>9.770 & <=13.420	53.67 (51.96, 55.37)	0.807	55.25 (53.75, 56.75)	0.889	56.16 (54.71, 57.61)	0.973	57.05 (55.64, 58.46)	0.909	58.53 (57.1, 59.95)	0.749	60.57 (59.07, 62.06)	0.656	64.09 (62.51, 65.68)	0.601	67.80 (66.01, 69.59)	0.579
	>13.420	54.42 (52.71, 56.13)	0.536	55.88 (54.37, 57.39)	0.491	56.69 (55.23, 58.15)	0.461	57.47 (56.06, 58.89)	0.442	58.8 (57.38, 60.21)	0.462	60.71 (59.24, 62.17)	0.517	64.1 (62.56, 65.63)	0.588	67.71 (65.97, 69.45)	0.636

∑allphth	<=19.780	54.03<br>(52.25, 55.80)		55.51<br>(53.95, 57.07)		56.35<br>(54.84, 57.86)		57.17<br>(55.70, 58.64)		58.55<br>(57.06, 60.05)		60.52<br>(58.95, 62.09)		63.96<br>(62.30, 65.62)		67.60<br>(65.74, 69.47)	
	>19.780 &<br><=30.900	53.30<br>(51.60, 55.00)	0.405	54.96<br>(53.45, 56.48)	0.470	55.94<br>(54.47, 57.41)	0.554	56.92<br>(55.5, 58.34)	0.700	58.53<br>(57.1, 59.95)	0.970	60.68<br>(59.20, 62.16)	0.822	64.31<br>(62.78, 65.85)	0.670	68.10<br>(66.37, 69.82)	0.597
	>30.900	54.64<br>(52.99, 56.30)	0.483	55.95<br>(54.52, 57.38)	0.569	56.65<br>(55.27, 58.02)	0.671	57.30<br>(55.98, 58.63)	0.838	58.46<br>(57.12, 59.8)	0.885	60.23<br>(58.83, 61.63)	0.694	63.47<br>(62.01, 64.94)	0.565	66.98<br>(65.32, 68.64)	0.507

Models were adjusted for age at measurement, BMI z-score at measurement, maternal education and family income at birth, and length of breastfeeding.

Table S5. Adjusted Associations Between Categorized Phthalate Metabolite Levels and Heartrate (Beats per Minute – BPM)

		5 year followup		8 year followup		10 year followup		14 year followup		17 year followup		20 year followup		22 year followup	
		Marginal Mean (95%CI)	p	Marginal Mean (95%CI)	p	Marginal Mean (95%CI)	p	Marginal Mean (95%CI)	p	Marginal Mean (95%CI)	p	Marginal Mean (95%CI)	p	Marginal Mean (95%CI)	p
MEP	<=1.750	86.08 (83.52, 88.64)		82.56 (80.31, 84.81)		78.18 (75.95, 80.4)		72.65 (70.41, 74.90)		69.80 (67.38, 72.21)		69.73 (67.13, 72.33)		72.09 (69.16, 75.01)	
	>1.750 & <=5.714	85.02 (82.48, 87.56)	0.373	81.46 (79.26, 83.65)	0.292	77.02 (74.86, 79.17)	0.223	71.42 (69.27, 73.56)	0.202	68.50 (66.20, 70.79)	0.243	68.36 (65.91, 70.82)	0.309	70.67 (67.90, 73.44)	0.358
	>5.714	84.21 (81.73, 86.68)	0.125	80.72 (78.65, 82.78)	0.087	76.36 (74.35, 78.37)	0.064	70.88 (68.86, 72.90)	0.074	68.06 (65.86, 70.26)	0.127	68.03 (65.65, 70.41)	0.215	70.41 (67.69, 73.13)	0.286
MiBP	<=0.792	86.68 (84.23, 89.13)	*	83.05 (80.87, 85.23)	*	78.49 (76.31, 80.67)	*	72.66 (70.46, 74.85)		69.52 (67.17, 71.88)		69.19 (66.65, 71.74)		71.39 (68.50, 74.27)	
	>0.792 & <=1.900	82.95 (80.46, 85.43)	0.002	79.75 (77.64, 81.86)	0.002	75.68 (73.61, 77.75)	0.003	70.54 (68.44, 72.63)	0.031	67.98 (65.70, 70.26)	0.177	68.24 (65.75, 70.74)	0.495	70.86 (68.00, 73.72)	0.741
	>1.900	85.15 (82.61, 87.7)	0.193	81.66 (79.54, 83.79)	0.178	77.26 (75.21, 79.3)	0.188	71.64 (69.58, 73.70)	0.286	68.69 (66.47, 70.90)	0.446	68.54 (66.19, 70.9)	0.624	70.87 (68.24, 73.51)	0.734
MnBP	<=1.999	85.13 (82.66, 87.59)		81.54 (79.36, 83.73)		77.10 (74.92, 79.27)		71.51 (69.32, 73.69)		68.60 (66.24, 70.95)		68.46 (65.90, 71.01)		70.74 (67.83, 73.65)	
	>1.999 & <=4.099	84.34 (81.80, 86.88)	0.508	80.78 (78.60, 82.96)	0.469	76.37 (74.24, 78.50)	0.444	70.82 (68.68, 72.95)	0.480	67.94 (65.65, 70.23)	0.560	67.84 (65.38, 70.30)	0.649	70.15 (67.37, 72.93)	0.703
	>4.099	85.12 (82.58, 87.67)	0.998	81.63 (79.51, 83.75)	0.937	77.28 (75.24, 79.33)	0.846	71.83 (69.78, 73.89)	0.736	69.04 (66.82, 71.26)	0.691	69.03 (66.64, 71.41)	0.676	71.40 (68.71, 74.09)	0.675
MHBP	<=0.220	84.52 (82.31, 86.72)		81.05 (79.19, 82.91)		76.73 (74.87, 78.59)		71.30 (69.43, 73.16)		68.52 (66.55, 70.49)		68.53 (66.50, 70.56)		70.94 (68.64, 73.23)	
	>0.220	85.85 (83.24, 88.45)	0.224	82.15 (79.88, 84.43)	0.257	77.56 (75.33, 79.80)	0.347	71.75 (69.49, 74.01)	0.614	68.66 (66.21, 71.11)	0.893	68.35 (65.68, 71.01)	0.882	70.52 (67.53, 73.51)	0.770
MBzP	<=0.260	85.06 (82.88, 87.25)		81.54 (79.65, 83.42)		77.14 (75.24, 79.04)		71.61 (69.71, 73.50)		68.74 (66.75, 70.74)		68.67 (66.60, 70.73)		71.01 (68.67, 73.36)	
	>0.260	84.51 (81.90, 87.12)	0.603	80.98 (78.80, 83.16)	0.549	76.58 (74.48, 78.68)	0.499	71.03 (68.90, 73.16)	0.496	68.16 (65.84, 70.49)	0.548	68.08 (65.57, 70.59)	0.616	70.42 (67.61, 73.23)	0.660
MEHP	<=2.960	83.89 (81.35, 86.43)		80.44 (78.30, 82.58)		76.14 (74.06, 78.21)		70.72 (68.63, 72.80)		67.94 (65.68, 70.20)		67.95 (65.52, 70.37)		70.34 (67.61, 73.07)	
	>2.960 & <=5.250	86.09 (83.60, 88.58)	0.070	82.53 (80.38, 84.67)	0.051	78.09 (75.97, 80.21)	0.043	72.49 (70.36, 74.62)	0.069	69.56 (67.27, 71.85)	0.146	69.41 (66.95, 71.86)	0.276	71.69 (68.92, 74.46)	0.379
	>5.250	84.67 (82.17, 87.17)	0.520	81.13 (78.96, 83.30)	0.515	76.73 (74.59, 78.87)	0.536	71.17 (69.02, 73.32)	0.640	68.28 (65.97, 70.59)	0.762	68.16 (65.66, 70.67)	0.872	70.47 (67.61, 73.33)	0.932
MECPP	<=0.785	85.72 (83.24, 88.20)		82.24 (80.11, 84.37)		77.91 (75.81, 80.00)		72.47 (70.36, 74.58)		69.69 (67.41, 71.98)		69.72 (67.22, 72.21)		72.14 (69.28, 74.99)	
	>0.785 & <=1.241	83.74 (81.18, 86.29)	0.101	80.36 (78.17, 82.54)	0.077	76.14 (74.00, 78.27)	0.067	70.86 (68.70, 73.02)	0.104	68.23 (65.88, 70.57)	0.199	68.39 (65.85, 70.93)	0.339	70.91 (68.06, 73.75)	0.441
	>1.241	85.07 (82.56, 87.58)	0.589	81.34 (79.19, 83.49)	0.395	76.71 (74.61, 78.82)	0.212	70.86 (68.77, 72.96)	0.095	67.75 (65.52, 69.98)	0.076	67.42 (65.05, 69.79)	0.082	69.59 (66.92, 72.25)	0.092
MCMHP	<=1.153	85.07 (82.55, 87.58)		81.62 (79.43, 83.81)		77.33 (75.17, 79.50)		71.97 (69.79, 74.16)		69.27 (66.91, 71.62)		69.35 (66.8, 71.89)		71.80 (68.93, 74.67)	

	>1.153 & <=1.810	83.97 (81.44, 86.50)	0.366	80.63 (78.49, 82.78)	0.357	76.48 (74.39, 78.57)	0.378	71.30 (69.20, 73.40)	0.496	68.75 (66.47, 71.02)	0.647	68.98 (66.52, 71.45)	0.791	71.54 (68.75, 74.34)	0.872
	>1.810	85.77 (83.28, 88.27)	0.551	81.93 (79.79, 84.07)	0.764	77.19 (75.09, 79.29)	0.880	71.19 (69.10, 73.28)	0.415	67.94 (65.71, 70.18)	0.230	67.48 (65.09, 69.86)	0.157	69.53 (66.84, 72.23)	0.135
MCPP	<=0.190	85.20 (82.87, 87.52)		81.74 (79.78, 83.71)		77.46 (75.51, 79.40)		72.10 (70.16, 74.05)		69.40 (67.33, 71.47)		69.48 (67.33, 71.64)		71.92 (69.49, 74.35)	
	>0.190	84.65 (82.33, 86.97)	0.582	80.95 (78.95, 82.95)	0.360	76.36 (74.37, 78.35)	0.165	70.59 (68.59, 72.59)	0.058	67.54 (65.40, 69.68)	0.042	67.26 (64.99, 69.54)	0.044	69.45 (66.88, 72.02)	0.050
MINP	<=2.363	85.41 (82.82, 88.00)		81.74 (79.62, 83.86)		77.23 (75.21, 79.25)		71.60 (69.56, 73.65)		68.69 (66.45, 70.94)		68.55 (66.13, 70.98)		70.83 (68.10, 73.56)	
	>2.363 & <=5.491	84.84 (82.28, 87.41)	0.649	81.10 (78.83, 83.36)	0.558	76.50 (74.26, 78.74)	0.459	70.75 (68.52, 72.99)	0.388	67.74 (65.36, 70.12)	0.398	67.5 (64.95, 70.05)	0.438	69.70 (66.83, 72.57)	0.469
	>5.491	84.53 (82.09, 86.97)	0.472	81.08 (78.93, 83.23)	0.542	76.83 (74.69, 78.97)	0.684	71.58 (69.44, 73.71)	0.978	68.97 (66.70, 71.25)	0.800	69.15 (66.70, 71.6)	0.656	71.65 (68.86, 74.44)	0.593
MCiOP	<=0.130	85.38 (82.92, 87.85)		81.9 (79.83, 83.97)		77.57 (75.55, 79.59)		72.17 (70.13, 74.21)		69.42 (67.20, 71.64)		69.47 (67.04, 71.89)		71.89 (69.10, 74.67)	
	>0.130	84.47 (82.20, 86.74)	0.391	80.87 (78.91, 82.83)	0.273	76.41 (74.44, 78.38)	0.170	70.82 (68.85, 72.78)	0.114	67.91 (65.85, 69.98)	0.123	67.80 (65.67, 69.92)	0.157	70.10 (67.73, 72.47)	0.187
MiDP	<=0.720	85.05 (82.91, 87.20)		81.48 (79.67, 83.30)		77.06 (75.23, 78.88)		71.50 (69.67, 73.33)		68.63 (66.70, 70.56)		68.54 (66.56, 70.51)		70.85 (68.62, 73.08)	
	>0.720	83.83 (80.75, 86.91)	0.351	80.34 (77.64, 83.03)	0.323	76.00 (73.41, 78.59)	0.317	70.57 (67.95, 73.20)	0.388	67.81 (64.92, 70.69)	0.506	67.82 (64.61, 71.04)	0.633	70.22 (66.60, 73.83)	0.710
∑MBP(i+n)	<=2.860	85.27 (82.77, 87.76)		81.72 (79.50, 83.94)		77.31 (75.09, 79.53)		71.78 (69.55, 74.00)		68.91 (66.53, 71.29)		68.83 (66.25, 71.41)		71.16 (68.23, 74.08)	
	>2.860 & <=5.986	84.20 (81.69, 86.70)	0.365	80.59 (78.45, 82.73)	0.281	76.12 (74.03, 78.22)	0.210	70.50 (68.39, 72.60)	0.189	67.56 (65.29, 69.83)	0.228	67.40 (64.96, 69.85)	0.294	69.68 (66.91, 72.45)	0.342
	>5.986	85.27 (82.73, 87.82)	0.998	81.78 (79.67, 83.89)	0.953	77.44 (75.41, 79.48)	0.890	72.01 (69.96, 74.05)	0.813	69.22 (67.01, 71.44)	0.780	69.23 (66.85, 71.60)	0.769	71.61 (68.93, 74.29)	0.769
∑DEHP	<=7.700	84.47 (81.86, 87.07)		81.05 (78.83, 83.26)		76.79 (74.65, 78.94)		71.48 (69.32, 73.64)		68.8 (66.46, 71.14)		68.90 (66.37, 71.42)		71.33 (68.50, 74.17)	
	>7.700 & <=11.320	85.06 (82.65, 87.47)	0.622	81.51 (79.42, 83.61)	0.661	77.11 (75.04, 79.18)	0.742	71.59 (69.51, 73.67)	0.912	68.74 (66.49, 70.98)	0.952	68.65 (66.24, 71.06)	0.855	70.96 (68.24, 73.68)	0.809
	>11.320	85.10 (82.55, 87.64)	0.609	81.43 (79.26, 83.61)	0.720	76.90 (74.77, 79.03)	0.915	71.19 (69.05, 73.32)	0.763	68.17 (65.89, 70.46)	0.575	67.92 (65.46, 70.39)	0.474	70.12 (67.30, 72.94)	0.435
∑DiNP	<=3.700	85.24 (82.63, 87.84)		81.54 (79.41, 83.68)		77.00 (74.96, 79.03)		71.30 (69.24, 73.35)		68.32 (66.07, 70.57)		68.13 (65.70, 70.56)		70.38 (67.66, 73.10)	
	>3.700 & <=8.292	85.05 (82.51, 87.59)	0.880	81.35 (79.13, 83.57)	0.860	76.80 (74.60, 78.99)	0.840	71.09 (68.90, 73.28)	0.833	68.11 (65.77, 70.44)	0.849	67.91 (65.40, 70.42)	0.871	70.16 (67.30, 73.01)	0.884
	>8.292	84.69 (82.23, 87.14)	0.655	81.27 (79.10, 83.43)	0.799	77.04 (74.88, 79.20)	0.964	71.79 (69.63, 73.95)	0.614	69.19 (66.89, 71.49)	0.437	69.39 (66.92, 71.86)	0.352	71.91 (69.10, 74.72)	0.322
∑DEHPDiNP	<=9.529	85.20 (82.57, 87.82)		81.68 (79.49, 83.87)		77.34 (75.24, 79.44)		71.93 (69.82, 74.05)		69.18 (66.88, 71.48)		69.19 (66.72, 71.67)		71.56 (68.79, 74.33)	
	>9.529 & <=12.850	85.07 (82.60, 87.55)	0.920	81.45 (79.3, 83.60)	0.831	76.98 (74.86, 79.11)	0.715	71.4 (69.25, 73.55)	0.591	68.50 (66.18, 70.83)	0.551	68.36 (65.85, 70.88)	0.547	70.62 (67.77, 73.47)	0.554
	>12.850	84.46 (81.99, 86.92)	0.543	80.85 (78.71, 83.00)	0.442	76.42 (74.29, 78.54)	0.339	70.87 (68.76, 72.99)	0.274	68.01 (65.76, 70.26)	0.289	67.90 (65.49, 70.31)	0.334	70.18 (67.42, 72.94)	0.369

∑low MWphth	<=5.550	85.60 (83.07, 88.13)		81.92 (79.68, 84.17)		77.35 (75.11, 79.58)		71.54 (69.29, 73.78)		68.43 (66.01, 70.85)		68.10 (65.48, 70.72)		70.27 (67.31, 73.23)	
	>5.550 & <=12.300	84.19 (81.65, 86.73)	0.238	80.80 (78.63, 82.97)	0.285	76.55 (74.43, 78.67)	0.404	71.2 (69.08, 73.32)	0.731	68.48 (66.21, 70.75)	0.965	68.55 (66.11, 70.99)	0.743	71.00 (68.22, 73.77)	0.641
	>12.300	84.81 (82.32, 87.31)	0.513	81.35 (79.27, 83.44)	0.590	77.03 (75.00, 79.05)	0.739	71.57 (69.53, 73.6)	0.974	68.76 (66.56, 70.95)	0.770	68.73 (66.38, 71.08)	0.641	71.11 (68.46, 73.77)	0.586
∑highMWphth	<=9.770	84.99 (82.37, 87.60)		81.46 (79.28, 83.64)		77.10 (75.01, 79.19)		71.66 (69.55, 73.76)		68.87 (66.58, 71.17)		68.85 (66.39, 71.32)		71.20 (68.44, 73.97)	
	>9.770 & <=13.420	85.02 (82.55, 87.49)	0.978	81.49 (79.35, 83.62)	0.980	77.12 (75.01, 79.23)	0.984	71.66 (69.52, 73.80)	0.993	68.87 (66.55, 71.19)	0.999	68.84 (66.32, 71.37)	0.994	71.19 (68.32, 74.05)	0.992
	>13.420	84.69 (82.21, 87.17)	0.808	81.05 (78.88, 83.22)	0.703	76.56 (74.41, 78.70)	0.575	70.93 (68.79, 73.06)	0.453	67.99 (65.73, 70.25)	0.425	67.81 (65.39, 70.23)	0.434	70.04 (67.28, 72.81)	0.449
∑allphth	<=19.780	85.28 (82.70, 87.85)		81.69 (79.43, 83.95)		77.23 (75.00, 79.46)		71.62 (69.36, 73.87)		68.69 (66.25, 71.13)		68.54 (65.89, 71.19)		70.81 (67.83, 73.8)	
	>19.780 & <=30.900	84.84 (82.31, 87.36)	0.712	81.23 (79.06, 83.40)	0.669	76.76 (74.63, 78.89)	0.627	71.13 (69.00, 73.27)	0.621	68.19 (65.91, 70.47)	0.659	68.02 (65.58, 70.47)	0.707	70.29 (67.52, 73.06)	0.738
	>30.900	84.62 (82.15, 87.09)	0.583	81.18 (79.10, 83.25)	0.634	76.90 (74.89, 78.92)	0.736	71.55 (69.52, 73.57)	0.942	68.83 (66.65, 71.02)	0.901	68.90 (66.56, 71.24)	0.794	71.33 (68.68, 73.98)	0.744

Models were adjusted for age at measurement, BMI z-score at measurement, maternal education and family income at birth, and length of breastfeeding. * Indicates a significant overall effect of the maternal serum phthalate levels on the outcome.

Table S6. Adjusted Associations Between Categorized Phthalate Metabolite Levels and Waist Circumference (cm)

		14 year followup		17 year followup		20 year followup		22 year followup	
		Marginal Geometric Mean (95%CI)	p	Marginal Geometric Mean (95%CI)	p	Marginal Geometric Mean (95%CI)	p	Marginal Geometric Mean (95%CI)	p
MEP	<=1.750	74.53 (71.40,77.80)		78.33 (75.19,81.59)		82.42 (79.10,85.88)		85.46 (81.87,89.19)	0.791
	>1.750 &	75.06 (72.13,78.11)	0.728	78.52 (75.61,81.55)	0.889	82.25 (79.18,85.44)	0.909	85.00 (81.69,88.43)	0.790
	>5.714	75.88 (73.06,78.82)	0.390	78.82 (76.05,81.69)	0.736	81.96 (79.04,84.98)	0.766	84.26 (81.11,87.53)	0.498
MiBP	<=0.792	73.72 (70.74,76.83)		77.27 (74.31,80.35)		81.09 (77.96,84.35)		83.91 (80.52,87.44)	
	>0.792 &	76.82 (73.83,79.92)	**0.045**	80.00 (77.06,83.05)	0.057	83.40 (80.29,86.64)	0.135	85.91 (82.53,89.42)	0.261
	>1.900	74.85 (72.05,77.75)	0.451	78.28 (75.50,81.17)	0.465	81.98 (79.05,85.02)	0.548	84.70 (81.56,87.97)	0.637
MnBP	<=1.999	75.18 (72.16,78.32)		78.51 (75.54,81.59)		82.08 (78.94,85.34)		84.71 (81.30,88.27)	
	>1.999 &	75.99 (72.96,79.14)	0.602	79.40 (76.41,82.51)	0.530	83.07 (79.94,86.33)	0.514	85.78 (82.42,89.28)	0.540
	>4.099	74.71 (71.91,77.62)	0.760	78.14 (75.36,81.03)	0.798	81.83 (78.89,84.88)	0.872	84.55 (81.38,87.85)	0.929
MHBP	<=0.220	75.33 (72.84,77.91)		78.90 (76.38,81.50)		82.74 (80.09,85.48)		85.57 (82.76,88.48)	
	>0.220	74.63 (71.45,77.94)	0.620	77.55 (74.46,80.77)	0.304	80.68 (77.45,84.03)	0.135	82.97 (79.50,86.59)	0.095
MBzP	<=0.260	75.22 (72.69,77.85)		78.76 (76.21,81.41)		82.57 (79.89,85.35)		85.39 (82.53,88.34)	
	>0.260	75.10 (72.14,78.17)	0.922	78.19 (75.26,81.22)	0.636	81.49 (78.41,84.69)	0.407	83.92 (80.60,87.38)	0.324
MEHP	<=2.960	76.83 (73.86,79.91)		80.08 (77.15,83.12)		83.57 (80.50,86.76)		86.14 (82.84,89.57)	
	>2.960 &	73.46 (70.62,76.41)	**0.026**	77.13 (74.32,80.04)	**0.035**	81.09 (78.11,84.18)	0.098	84.02 (80.78,87.39)	0.214
	>5.250	75.40 (72.45,78.47)	0.348	78.68 (75.75,81.72)	0.320	82.19 (79.10,85.41)	0.360	84.78 (81.43,88.27)	0.428
MECPP	<=0.785	75.78 (72.83,78.86)		79.41 (76.48,82.45)		83.32 (80.21,86.56)		86.21 (82.82,89.75)	
	>0.785 &	75.63 (72.59,78.80)	0.923	78.65 (75.66,81.75)	0.600	81.87 (78.75,85.12)	0.352	84.23 (80.88,87.73)	0.267
	>1.241	74.39 (71.58,77.32)	0.349	77.91 (75.12,80.81)	0.277	81.70 (78.76,84.76)	0.273	84.50 (81.34,87.79)	0.311
MCMHP	<=1.153	76.72 (73.65,79.92)		79.98 (76.94,83.13)		83.46 (80.25,86.80)		86.02 (82.55,89.64)	
	>1.153 &	75.62 (72.68,78.69)	0.483	78.89 (76.00,81.88)	0.454	82.39 (79.36,85.53)	0.489	84.96 (81.69,88.36)	0.549
	>1.810	73.81 (71.03,76.70)	0.050	77.43 (74.65,80.31)	0.065	81.33 (78.39,84.38)	0.150	84.21 (81.04,87.50)	0.284
MCPP	<=0.190	75.92 (73.27,78.67)		79.47 (76.81,82.23)		83.29 (80.48,86.19)		86.10 (83.11,89.20)	
	>0.190	74.35 (71.64,77.17)	0.209	77.56 (74.87,80.35)	0.099	81.01 (78.18,83.93)	0.064	83.54 (80.51,86.68)	0.067
MINP	<=2.363	76.13 (73.27,79.10)		79.25 (76.44,82.17)		82.60 (79.64,85.67)		85.06 (81.86,88.38)	
	>2.363 &	75.17 (72.13,78.35)	0.537	78.63 (75.61,81.77)	0.664	82.35 (79.17,85.66)	0.870	85.09 (81.66,88.66)	0.984
	>5.491	74.15 (71.24,77.18)	0.189	77.74 (74.84,80.75)	0.279	81.60 (78.53,84.79)	0.505	84.46 (81.14,87.90)	0.724
MCiOP	<=0.130	74.79 (71.97,77.72)		78.06 (75.29,80.94)		81.57 (78.63,84.63)		84.16 (80.95,87.49)	
	>0.130	75.57 (72.93,78.32)	0.555	79.03 (76.36,81.80)	0.434	82.75 (79.94,85.65)	0.376	85.49 (82.52,88.56)	0.379
MiDP	<=0.720	75.07 (72.64,77.58)		78.46 (76.00,81.00)		82.11 (79.52,84.78)		84.79 (82.06,87.62)	
	>0.720	76.54 (72.76,80.52)	0.386	79.98 (76.28,83.85)	0.337	83.67 (79.76,87.78)	0.354	86.39 (82.11,90.90)	0.405
∑MBP(i+n)	<=2.860	74.86 (71.78,78.08)		78.19 (75.15,81.35)		81.76 (78.56,85.08)		84.39 (80.94,87.98)	
	>2.860 &	75.77 (72.82,78.85)	0.554	79.33 (76.40,82.37)	0.420	83.16 (80.07,86.37)	0.355	85.99 (82.65,89.46)	0.356
	>5.986	74.97 (72.17,77.88)	0.945	78.28 (75.50,81.15)	0.951	81.82 (78.90,84.86)	0.965	84.44 (81.28,87.71)	0.977
∑DEHP	<=7.700	77.42 (74.34,80.62)		80.54 (77.51,83.68)		83.87 (80.69,87.18)		86.32 (82.89,89.89)	
	>7.700 &	73.77 (70.96,76.70)	**0.017**	77.28 (74.51,80.17)	**0.021**	81.07 (78.14,84.10)	0.063	83.86 (80.70,87.14)	0.151
	>11.320	74.99 (72.12,77.97)	0.109	78.54 (75.67,81.51)	0.156	82.36 (79.31,85.52)	0.318	85.18 (81.87,88.62)	0.510
∑DiNP	<=3.700	75.36 (72.50,78.33)		78.45 (75.64,81.37)		81.77 (78.81,84.83)		84.20 (81.02,87.51)	
	>3.700 &	75.87 (72.87,79.00)	0.740	79.35 (76.38,82.44)	0.530	83.09 (79.95,86.35)	0.383	85.85 (82.46,89.37)	0.338
	>8.292	74.50 (71.53,77.58)	0.569	78.11 (75.15,81.18)	0.807	82.00 (78.88,85.25)	0.877	84.88 (81.51,88.38)	0.692
∑DEHPDiNP	<=9.529	75.58 (72.64,78.63)		78.77 (75.89,81.77)		82.20 (79.17,85.35)		84.72 (81.44,88.13)	
	>9.529 &	75.41 (72.39,78.55)	0.915	78.96 (75.98,82.07)	0.896	82.79 (79.64,86.06)	0.703	85.62 (82.22,89.15)	0.610
	>12.850	74.72 (71.84,77.72)	0.571	78.15 (75.28,81.13)	0.658	81.84 (78.81,84.99)	0.811	84.56 (81.30,87.96)	0.927
∑low MWphth	<=5.550	73.67 (70.61,76.86)		77.33 (74.27,80.51)		81.28 (78.03,84.66)		84.20 (80.68,87.87)	
	>5.550 &	76.18 (73.18,79.30)	0.100	79.43 (76.48,82.50)	0.138	82.92 (79.82,86.13)	0.283	85.48 (82.15,88.95)	0.464
	>12.300	75.33 (72.57,78.18)	0.271	78.60 (75.88,81.43)	0.364	82.12 (79.24,85.11)	0.575	84.71 (81.60,87.94)	0.765
∑highMWphth	<=9.770	75.78 (72.84,78.84)		78.95 (76.07,81.95)		82.35 (79.32,85.50)		84.85 (81.58,88.25)	
	>9.770 &	75.42 (72.43,78.53)	0.816	78.92 (75.96,81.99)	0.980	82.68 (79.55,85.93)	0.832	85.45 (82.07,88.98)	0.731
	>13.420	74.48 (71.58,77.50)	0.391	77.98 (75.09,80.98)	0.492	81.75 (78.70,84.92)	0.690	84.53 (81.25,87.95)	0.854
∑allphth	<=19.780	75.50 (72.35,78.80)		78.68 (75.56,81.94)		82.09 (78.8,85.52)		84.60 (81.05,88.30)	
	>19.780	74.45 (71.49,77.52)	0.492	78.42 (75.47,81.48)	0.852	82.72 (79.6,85.95)	0.684	85.91 (82.55,89.40)	0.454
	>30.900	75.70 (72.92,78.58)	0.901	78.69 (75.97,81.52)	0.995	81.90 (79.04,84.86)	0.899	84.25 (81.17,87.44)	0.840

Models were adjusted for age at measurement, maternal education and family income at birth, and length of breastfeeding.

Table S7. Adjusted Associations Between Categorized Phthalate Metabolite Levels and Alanine Transaminase (ALT, U/L)

		14 year followup		17 year followup		20 year followup		22 year followup	
		Marginal Geometric Mean (95%CI)	p	Marginal Geometric Mean (95%CI)	p	Marginal Geometric Mean (95%CI)	p	Marginal Geometric Mean (95%CI)	p
MEP	<=1.750	16.86 (14.93,19.04)		21.44 (19.05,24.13)		29.90 (26.39,33.88)		31.83 (27.68,36.59)	
	>1.750 &	15.97 (14.23,17.93)	0.336	20.74 (18.52,23.22)	0.487	29.55 (26.25,33.27)	0.842	31.94 (27.98,36.45)	0.963
	>5.714	15.55 (13.92,17.36)	0.158	19.53 (17.50,21.79)	0.057	26.89 (23.94,30.20)	0.078	28.36 (24.82,32.40)	0.130
MiBP	<=0.792	16.24 (14.41,18.30)		20.38 (18.14,22.89)		27.77 (24.54,31.43)		29.29 (25.48,33.67)	
	>0.792 &	15.42 (13.71,17.35)	0.378	20.36 (18.15,22.84)	0.989	29.24 (25.85,33.07)	0.410	31.99 (27.71,36.93)	0.268
	>1.900	16.10 (14.43,17.98)	0.882	20.52 (18.39,22.89)	0.883	28.41 (25.35,31.84)	0.696	30.29 (26.74,34.31)	0.644
MnBP	<=1.999	16.31 (14.51,18.33)		20.46 (18.26,22.92)		27.93 (24.70,31.60)		29.35 (25.45,33.84)	
	>1.999 &	15.57 (13.82,17.54)	0.416	20.23 (18.01,22.71)	0.814	28.63 (25.39,32.28)	0.683	30.85 (27.00,35.26)	0.515
	>4.099	15.89 (14.21,17.76)	0.649	20.39 (18.24,22.78)	0.944	28.49 (25.34,32.03)	0.743	30.43 (26.71,34.66)	0.637
MHBP	<=0.220	15.92 (14.43,17.57)		20.30 (18.35,22.46)		28.27 (25.55,31.29)		30.09 (26.97,33.56)	
	>0.220	16.09 (14.17,18.27)	0.845	20.75 (18.38,23.43)	0.628	29.25 (25.62,33.39)	0.543	31.39 (27.04,36.44)	0.549
MBzP	<=0.260	15.86 (14.33,17.55)		20.25 (18.28,22.42)		28.17 (25.42,31.22)		29.96 (26.78,33.51)	
	>0.260	16.16 (14.38,18.16)	0.707	20.82 (18.52,23.41)	0.509	29.26 (25.83,33.14)	0.470	31.32 (27.30,35.93)	0.500
MEHP	<=2.960	15.27 (13.61,17.14)		20.38 (18.22,22.81)		29.69 (26.34,33.47)		32.25 (28.29,36.77)	
	>2.960 &	15.68 (14.02,17.54)	0.637	20.47 (18.31,22.89)	0.930	29.15 (25.88,32.82)	0.755	31.15 (27.23,35.64)	0.642
	>5.250	16.99 (15.09,19.14)	0.057	20.53 (18.27,23.07)	0.882	27.00 (23.90,30.50)	0.110	27.27 (23.71,31.37)	**0.026**
MECPP	<=0.785	15.12 (13.46,17.00)		20.43 (18.22,22.92)		30.25 (26.78,34.17)		33.86 (29.42,38.98)	
	>0.785 &	16.21 (14.43,18.22)	0.230	20.35 (18.13,22.83)	0.930	27.92 (24.70,31.55)	0.193	29.62 (25.83,33.96)	0.088
	>1.241	16.44 (14.69,18.40)	0.131	20.34 (18.22,22.70)	0.921	27.50 (24.50,30.86)	0.102	28.87 (25.41,32.80)	**0.032**
MCMHP	<=1.153	15.51 (13.78,17.46)		20.51 (18.23,23.06)		29.78 (26.25,33.78)		32.65 (28.32,37.65)	
	>1.153 &	15.44 (13.75,17.32)	0.930	19.62 (17.52,21.96)	0.366	27.36 (24.33,30.76)	0.165	29.15 (25.49,33.34)	0.145
	>1.810	16.95 (15.16,18.95)	0.109	21.06 (18.87,23.49)	0.577	28.69 (25.54,32.23)	0.527	30.07 (26.44,34.19)	0.270
MCPP	<=0.190	16.29 (14.72,18.03)		21.15 (19.08,23.43)		29.97 (26.97,33.30)		32.16 (28.67,36.08)	
	>0.190	16.70 (15.03,18.56)	0.5947	20.97 (18.89,23.29)	0.838	28.74 (25.77,32.06)	0.393	30.12 (26.62,34.07)	0.291
MINP	<=2.363	15.26 (13.66,17.04)	*	19.91 (17.83,22.24)		28.53 (25.31,32.16)		30.90 (27.06,35.30)	
	>2.363 &	17.58 (15.57,19.86)	**0.013**	21.28 (18.91,23.94)	0.176	28.21 (24.94,31.90)	0.851	28.92 (25.19,33.20)	0.383
	>5.491	15.66 (13.96,17.57)	0.644	20.54 (18.34,22.99)	0.522	29.58 (26.28,33.30)	0.546	32.16 (28.11,36.80)	0.599
MCiOP	<=0.130	14.86 (13.27,16.64)		19.61 (17.58,21.89)		28.17 (25.04,31.68)		30.70 (26.80,35.17)	
	>0.130	16.59 (14.98,18.37)	**0.027**	21.01 (18.92,23.34)	0.104	28.92 (26.00,32.17)	0.617	30.58 (27.30,34.26)	0.956
MiDP	<=0.720	16.01 (14.53,17.63)		20.43 (18.50,22.57)		28.45 (25.74,31.44)		30.30 (27.21,33.73)	
	>0.720	15.05 (12.95,17.50)	0.334	19.51 (16.92,22.50)	0.398	27.59 (23.63,32.21)	0.644	29.71 (24.90,35.43)	0.813
∑MBP(i+n)	<=2.860	16.42 (14.56,18.51)		20.50 (18.24,23.04)		27.86 (24.60,31.56)		29.19 (25.32,33.65)	
	>2.860 &	15.51 (13.82,17.40)	0.311	20.34 (18.18,22.77)	0.872	29.08 (25.84,32.73)	0.476	31.58 (27.63,36.08)	0.306
	>5.986	15.93 (14.25,17.81)	0.602	20.36 (18.22,22.75)	0.890	28.34 (25.22,31.85)	0.778	30.20 (26.53,34.37)	0.657
∑DEHP	<=7.700	15.25 (13.59,17.10)	*	20.88 (18.66,23.36)		31.21 (27.67,35.20)		34.50 (30.15,39.49)	
	>7.700 &	16.55 (14.85,18.44)	0.144	21.06 (18.92,23.45)	0.856	29.20 (26.07,32.72)	0.264	30.61 (26.90,34.83)	0.112
	>11.320	17.56 (15.67,19.68)	**0.012**	21.42 (19.15,23.95)	0.599	28.42 (25.26,31.98)	0.121	28.88 (25.22,33.07)	**0.020**
∑DiNP	<=3.700	15.10 (13.51,16.86)		19.81 (17.74,22.12)		28.54 (25.34,32.16)		30.98 (27.15,35.35)	
	>3.700 &	17.11 (15.19,19.27)	**0.029**	20.85 (18.58,23.40)	0.296	27.85 (24.66,31.45)	0.683	28.64 (24.95,32.87)	0.304
	>8.292	16.08 (14.32,18.07)	0.261	20.88 (18.62,23.40)	0.281	29.76 (26.41,33.53)	0.491	32.04 (27.97,36.71)	0.657
∑DEHPDiNP	<=9.529	15.13 (13.50,16.96)		19.59 (17.49,21.94)		27.85 (24.64,31.48)		29.82 (26.05,34.13)	
	>9.529 &	15.70 (13.95,17.68)	0.520	20.69 (18.44,23.21)	0.271	29.94 (26.54,33.78)	0.234	32.47 (28.36,37.16)	0.268
	>12.850	16.87 (15.06,18.89)	0.051	20.73 (18.53,23.18)	0.241	27.92 (24.83,31.41)	0.965	28.78 (25.13,32.96)	0.640
∑low MWphth	<=5.550	16.39 (14.52,18.51)		20.67 (18.35,23.27)		28.31 (24.92,32.16)		29.92 (25.94,34.51)	
	>5.550 &	15.09 (13.43,16.97)	0.150	19.99 (17.84,22.39)	0.495	28.80 (25.56,32.46)	0.777	31.55 (27.54,36.15)	0.493
	>12.300	16.33 (14.64,18.22)	0.947	20.6 (18.47,22.98)	0.948	28.24 (25.19,31.66)	0.966	29.85 (26.28,33.92)	0.977
∑highMWphth	<=9.770	15.02 (13.42,16.82)		19.83 (17.73,22.18)		28.77 (25.49,32.48)		31.23 (27.31,35.70)	
	>9.770 &	16.29 (14.48,18.33)	0.156	20.78 (18.52,23.32)	0.339	29.13 (25.79,32.89)	0.842	30.84 (26.89,35.37)	0.872
	>13.420	16.66 (14.85,18.68)	0.067	20.68 (18.47,23.15)	0.385	28.18 (25.04,31.72)	0.729	29.26 (25.54,33.51)	0.390
∑allphth	<=19.780	15.76 (13.93,17.82)		20.46 (18.14,23.08)		29.00 (25.45,33.05)		31.25 (27.00,36.17)	
	>19.780	15.62 (13.91,17.54)	0.878	20.17 (18.02,22.59)	0.770	28.44 (25.27,32.00)	0.745	30.52 (26.71,34.87)	0.759
	>30.900	16.37 (14.67,18.27)	0.510	20.51 (18.40,22.88)	0.959	28.04 (25.02,31.42)	0.582	29.44 (25.89,33.47)	0.444

Models were adjusted for age at measurement, BMI z-score at measurement, maternal education and family income at birth, and length of breastfeeding. * Indicates a significant overall effect of the maternal serum phthalate levels on the outcome.

Table S8. Adjusted Associations Between Categorized Phthalate Metabolite Levels and Gamma-Glutamyl Transferase (GGT, U/L)

		14 year followup		17 year followup		20 year followup		22 year followup	
		Marginal Geometric Mean (95%CI)	p	Marginal Geometric Mean (95%CI)	p	Marginal Geometric Mean (95%CI)	p	Marginal Geometric Mean (95%CI)	p
MEP	<=1.750	11.73 (10.44,13.18)		14.30 (12.75,16.03)		16.97 (15.02,19.18)		19.00 (16.61,21.72)	
	>1.750 & <=5.714	11.12 (9.96,12.41)	0.300	14.14 (12.69,15.75)	0.817	17.52 (15.63,19.65)	0.584	20.22 (17.83,22.94)	0.370
	>5.714	10.97 (9.88,12.19)	0.206	13.56 (12.22,15.05)	0.295	16.33 (14.59,18.28)	0.520	18.46 (16.28,20.94)	0.691
MiBP	<=0.792	11.47 (10.24,12.84)		14.29 (12.79,15.97)		17.34 (15.41,19.52)		19.69 (17.26,22.46)	
	>0.792 & <=1.900	11.11 (9.94,12.42)	0.553	13.81 (12.38,15.42)	0.500	16.72 (14.84,18.84)	0.548	18.95 (16.56,21.68)	0.602
	>1.900	11.11 (10,12.34)	0.530	13.79 (12.43,15.30)	0.459	16.67 (14.93,18.61)	0.490	18.87 (16.73,21.29)	0.538
MnBP	<=1.999	11.48 (10.29,12.81)		14.40 (12.93,16.04)		17.58 (15.63,19.76)		20.04 (17.55,22.87)	
	>1.999 & <=4.099	10.68 (9.54,11.96)	0.164	13.34 (11.94,14.90)	0.117	16.20 (14.42,18.21)	0.167	18.41 (16.20,20.92)	0.234
	>4.099	11.28 (10.15,12.54)	0.735	13.90 (12.52,15.44)	0.479	16.67 (14.90,18.65)	0.372	18.77 (16.58,21.24)	0.359
MHBP	<=0.220	11.08 (10.08,12.18)		13.88 (12.63,15.25)		16.94 (15.37,18.67)		19.33 (17.41,21.45)	
	>0.220	11.61 (10.30,13.08)	0.335	14.10 (12.54,15.85)	0.735	16.66 (14.69,18.91)	0.761	18.58 (16.14,21.39)	0.547
MBzP	<=0.260	11.27 (10.23,12.41)		13.97 (12.70,15.37)		16.85 (15.27,18.60)		19.05 (17.12,21.20)	
	>0.260	11.06 (9.90,12.37)	0.690	13.86 (12.41,15.49)	0.857	16.91 (15.01,19.04)	0.954	19.25 (16.88,21.96)	0.864
MEHP	<=2.960	11.34 (10.16,12.65)		14.08 (12.65,15.68)		17.00 (15.16,19.05)	*	19.20 (16.94,21.75)	*
	>2.960 & <=5.250	11.14 (10.01,12.39)	0.734	14.43 (12.99,16.04)	0.616	18.19 (16.24,20.37)	0.240	21.19 (18.68,24.04)	0.153
	>5.250	11.18 (10.00,12.51)	0.792	13.40 (12.01,14.96)	0.308	15.59 (13.88,17.51)	0.133	17.16 (15.08,19.52)	0.103
MECPP	<=0.785	10.74 (9.62,11.98)		13.83 (12.41,15.40)		17.39 (15.48,19.53)		20.27 (17.79,23.10)	
	>0.785 & <=1.241	11.90 (10.65,13.29)	0.052	14.63 (13.12,16.32)	0.258	17.54 (15.60,19.72)	0.882	19.77 (17.37,22.51)	0.732
	>1.241	11.06 (9.95,12.29)	0.561	13.47 (12.15,14.95)	0.586	16.01 (14.34,17.87)	0.147	17.93 (15.89,20.24)	0.074
MCMHP	<=1.153	11.27 (10.07,12.62)		14.00 (12.51,15.67)		16.93 (15.00,19.10)		19.17 (16.75,21.93)	
	>1.153 & <=1.810	11.06 (9.91,12.34)	0.716	13.90 (12.49,15.48)	0.891	17.03 (15.19,19.09)	0.921	19.46 (17.13,22.10)	0.836
	>1.810	11.26 (10.12,12.52)	0.980	13.89 (12.51,15.43)	0.871	16.68 (14.92,18.65)	0.801	18.80 (16.63,21.26)	0.781
MCPP	<=0.190	11.07 (10.02,12.25)		14.00 (12.67,15.48)		17.23 (15.52,19.13)		19.75 (17.64,22.11)	
	>0.190	11.39 (10.28,12.62)	0.510	13.86 (12.53,15.34)	0.808	16.41 (14.75,18.26)	0.311	18.30 (16.27,20.58)	0.186
MINP	<=2.363	10.73 (9.66,11.91)		13.75 (12.40,15.25)		17.15 (15.32,19.19)		19.83 (17.50,22.48)	
	>2.363 & <=5.491	11.05 (9.86,12.39)	0.566	13.53 (12.10,15.14)	0.749	16.10 (14.30,18.12)	0.284	18.01 (15.81,20.52)	0.172
	>5.491	11.90 (10.67,13.28)	0.042	14.66 (13.17,16.33)	0.187	17.55 (15.66,19.67)	0.690	19.72 (17.37,22.38)	0.934
MCiOP	<=0.130	11.07 (9.95,12.33)		13.85 (12.48,15.38)		16.87 (15.06,18.89)		19.19 (16.90,21.81)	
	>0.130	11.28 (10.21,12.45)	0.692	13.99 (12.67,15.46)	0.815	16.90 (15.25,18.72)	0.972	19.12 (17.13,21.33)	0.948
MiDP	<=0.720	11.15 (10.16,12.23)		13.92 (12.68,15.27)		16.93 (15.39,18.63)		19.27 (17.39,21.36)	
	>0.720	11.82 (10.26,13.61)	0.320	14.34 (12.49,16.47)	0.590	16.95 (14.59,19.70)	0.986	18.91 (15.98,22.37)	0.805
∑MBP(i+n)	<=2.860	11.73 (10.48,13.13)		14.49 (12.97,16.19)		17.41 (15.45,19.61)		19.63 (17.18,22.42)	
	>2.860 & <=5.986	10.68 (9.58,11.91)	0.066	13.57 (12.19,15.10)	0.175	16.78 (14.97,18.81)	0.532	19.32 (17.02,21.93)	0.821
	>5.986	11.29 (10.16,12.54)	0.462	13.85 (12.47,15.37)	0.363	16.53 (14.78,18.48)	0.379	18.54 (16.39,20.98)	0.423
∑DEHP	<=7.700	11.07 (9.89,12.39)		14.21 (12.72,15.87)		17.72 (15.74,19.95)		20.48 (17.97,23.33)	
	>7.700 & <=11.320	11.35 (10.21,12.61)	0.634	14.04 (12.65,15.59)	0.811	16.87 (15.08,18.87)	0.399	18.98 (16.76,21.50)	0.279
	>11.320	11.12 (9.96,12.42)	0.935	13.58 (12.19,15.14)	0.358	16.10 (14.35,18.06)	0.102	17.95 (15.80,20.39)	0.061
∑DiNP	<=3.700	10.63 (9.57,11.81)		13.57 (12.23,15.06)		16.85 (15.05,18.86)		19.41 (17.12,22.01)	
	>3.700 & <=8.292	11.19 (10.01,12.51)	0.327	13.80 (12.38,15.40)	0.732	16.54 (14.73,18.58)	0.758	18.60 (16.35,21.15)	0.543
	>8.292	12.05 (10.79,13.45)	0.016	14.80 (13.27,16.50)	0.080	17.66 (15.73,19.83)	0.424	19.79 (17.41,22.50)	0.785
∑DEHPDiNP	<=9.529	10.63 (9.54,11.85)		13.58 (12.19,15.13)		16.85 (14.99,18.95)		19.41 (17.06,22.09)	
	>9.529 & <=12.850	11.32 (10.13,12.67)	0.234	14.11 (12.65,15.74)	0.449	17.06 (15.20,19.15)	0.836	19.30 (16.99,21.93)	0.935
	>12.850	11.66 (10.47,12.99)	0.072	14.22 (12.78,15.82)	0.352	16.82 (15.01,18.85)	0.973	18.73 (16.50,21.26)	0.610
∑low MWphth	<=5.550	11.44 (10.19,12.85)		14.04 (12.53,15.73)		16.78 (14.85,18.96)		18.86 (16.47,21.59)	
	>5.550 & <=12.300	11.48 (10.28,12.83)	0.948	14.48 (12.99,16.14)	0.534	17.80 (15.85,19.99)	0.321	20.41 (17.94,23.22)	0.270
	>12.300	10.91 (9.84,12.10)	0.353	13.52 (12.20,14.97)	0.438	16.32 (14.63,18.19)	0.633	18.47 (16.37,20.84)	0.767
∑highMWphth	<=9.770	10.68 (9.59,11.89)		13.70 (12.31,15.24)		17.07 (15.21,19.17)		19.73 (17.36,22.42)	
	>9.770 & <=13.420	11.27 (10.08,12.61)	0.301	13.96 (12.51,15.58)	0.706	16.78 (14.93,18.86)	0.770	18.89 (16.60,21.50)	0.547
	>13.420	11.73 (10.52,13.09)	0.068	14.32 (12.86,15.96)	0.366	16.97 (15.13,19.03)	0.914	18.91 (16.65,21.47)	0.546
∑allphth	<=19.780	11.42 (10.16,12.84)		14.22 (12.67,15.96)		17.22 (15.21,19.51)		19.53 (17.01,22.42)	
	>19.780 & <=30.900	10.73 (9.61,11.97)	0.225	13.64 (12.24,15.19)	0.393	16.88 (15.05,18.93)	0.729	19.42 (17.11,22.05)	0.941
	>30.900	11.51 (10.38,12.76)	0.887	14.00 (12.64,15.50)	0.754	16.55 (14.85,18.46)	0.510	18.45 (16.35,20.83)	0.434

Models were adjusted for age at measurement, BMI z-score at measurement, maternal education and family income at birth, and length of breastfeeding. * Indicates a significant overall effect of the maternal serum phthalate levels on the outcome.

Table S9. Adjusted Associations Between Categorized Phthalate Metabolite Levels and Aspartate Transaminase (AST, U/L)

		17 year followup		20 year followup		22 year followup	
		Marginal Geometric Mean (95%CI)	p	Marginal Geometric Mean (95%CI)	p	Marginal Geometric Mean (95%CI)	p
MEP	<=1.750	25.41 (23.09,27.96)		26.75 (24.41,29.32)		27.90 (24.97,31.17)	
	>1.750 & <=5.714	25.00 (22.69,27.54)	0.742	26.27 (24.07,28.67)	0.669	27.36 (24.64,30.38)	0.747
	>5.714	24.57 (22.37,26.99)	0.511	25.84 (23.75,28.11)	0.426	26.93 (24.26,29.88)	0.566
MiBP	<=0.792	24.08 (21.90,26.48)		25.55 (23.35,27.95)		26.78 (23.97,29.92)	
	>0.792 & <=1.900	26.11 (23.62,28.85)	0.124	27.08 (24.73,29.66)	0.199	27.93 (24.95,31.27)	0.519
	>1.900	24.78 (22.62,27.14)	0.562	26.13 (24.06,28.37)	0.594	27.26 (24.73,30.05)	0.763
MnBP	<=1.999	23.95 (21.78,26.35)		25.11 (22.98,27.45)		26.10 (23.32,29.22)	
	>1.999 & <=4.099	26.28 (23.92,28.89)	0.066	27.07 (24.81,29.53)	0.082	27.78 (25.04,30.82)	0.320
	>4.099	24.63 (22.43,27.05)	0.587	26.48 (24.32,28.83)	0.222	28.02 (25.30,31.03)	0.257
MHBP	<=0.220	24.72 (22.82,26.78)		26.11 (24.24,28.13)		27.29 (25.08,29.69)	
	>0.220	25.43 (23.00,28.11)	0.540	26.45 (24.03,29.12)	0.747	27.35 (24.28,30.81)	0.969
MBzP	<=0.260	24.95 (23.05,27.01)		26.06 (24.16,28.11)		27.02 (24.77,29.48)	
	>0.260	24.89 (22.47,27.57)	0.957	26.52 (24.22,29.04)	0.644	27.89 (25.04,31.07)	0.548
MEHP	<=2.960	25.84 (23.53,28.37)		26.64 (24.46,29.02)		27.37 (24.73,30.28)	
	>2.960 & <=5.250	24.70 (22.50,27.12)	0.372	26.48 (24.27,28.88)	0.883	27.95 (25.11,31.11)	0.724
	>5.250	24.05 (21.79,26.54)	0.154	25.39 (23.19,27.79)	0.260	26.51 (23.72,29.64)	0.605
MECPP	<=0.785	24.13 (21.81,26.70)		27.11 (24.79,29.65)		29.60 (26.50,33.05)	
	>0.785 & <=1.241	25.41 (23.07,27.99)	0.332	26.12 (23.89,28.56)	0.402	26.78 (24.05,29.82)	0.114
	>1.241	24.91 (22.78,27.25)	0.529	25.55 (23.50,27.77)	0.156	26.15 (23.67,28.89)	0.039
MCMHP	<=1.153	24.88 (22.53,27.48)		26.95 (24.56,29.58)		28.68 (25.62,32.11)	
	>1.153 & <=1.810	25.31 (22.96,27.89)	0.753	26.37 (24.21,28.71)	0.620	27.30 (24.58,30.31)	0.433
	>1.810	24.76 (22.61,27.11)	0.921	25.60 (23.51,27.87)	0.225	26.35 (23.81,29.16)	0.161
MCPP	<=0.190	25.28 (23.25,27.49)		26.84 (24.81,29.04)		28.15 (25.72,30.82)	
	>0.190	24.47 (22.40,26.74)	0.437	25.41 (23.41,27.58)	0.118	26.23 (23.77,28.95)	0.159
MINP	<=2.363	26.47 (24.08,29.11)		26.78 (24.58,29.17)		27.14 (24.46,30.10)	
	>2.363 & <=5.491	24.55 (22.31,27.01)	0.140	25.87 (23.66,28.29)	0.425	26.99 (24.22,30.08)	0.930
	>5.491	23.74 (21.61,26.09)	0.032	25.83 (23.67,28.19)	0.407	27.57 (24.79,30.65)	0.796
MCiOP	<=0.130	24.35 (22.20,26.70)		26.51 (24.33,28.89)		28.32 (25.43,31.55)	
	>0.130	25.21 (23.20,27.39)	0.427	26.09 (24.12,28.23)	0.675	26.89 (24.60,29.38)	0.342
MiDP	<=0.720	24.87 (23.02,26.86)		26.20 (24.35,28.19)		27.33 (25.16,29.69)	
	>0.720	25.36 (22.33,28.80)	0.734	26.40 (23.56,29.58)	0.875	27.31 (23.77,31.38)	0.990
∑MBP(i+n)	<=2.860	24.15 (21.92,26.61)		25.29 (23.09,27.70)		26.27 (23.48,29.40)	
	>2.860 & <=5.986	25.66 (23.35,28.21)	0.229	26.74 (24.53,29.16)	0.197	27.69 (24.93,30.75)	0.402
	>5.986	24.82 (22.59,27.26)	0.595	26.37 (24.22,28.70)	0.339	27.67 (24.99,30.63)	0.402
∑DEHP	<=7.700	25.48 (23.12,28.09)		26.88 (24.59,29.39)		28.07 (25.23,31.23)	
	>7.700 & <=11.320	24.69 (22.49,27.11)	0.537	26.44 (24.32,28.74)	0.692	27.89 (25.20,30.88)	0.916
	>11.320	24.48 (22.26,26.92)	0.427	25.14 (22.98,27.50)	0.122	25.75 (23.07,28.74)	0.160
∑DiNP	<=3.700	25.97 (23.59,28.59)		26.89 (24.69,29.30)		27.72 (25.02,30.71)	
	>3.700 & <=8.292	24.60 (22.39,27.02)	0.292	25.57 (23.39,27.95)	0.245	26.42 (23.69,29.47)	0.433
	>8.292	24.07 (21.88,26.49)	0.144	25.92 (23.72,28.32)	0.399	27.47 (24.68,30.57)	0.881
∑DEHPDiNP	<=9.529	26.06 (23.67,28.69)		26.44 (24.24,28.84)		26.86 (24.20,29.81)	
	>9.529 & <=12.850	24.71 (22.43,27.23)	0.309	26.97 (24.70,29.46)	0.648	28.86 (25.94,32.10)	0.240
	>12.850	24.07 (21.95,26.40)	0.115	25.22 (23.10,27.53)	0.272	26.21 (23.55,29.17)	0.686
∑low MWphth	<=5.550	25.36 (23.03,27.92)		26.41 (24.07,28.98)		27.33 (24.39,30.61)	
	>5.550 & <=12.300	25.00 (22.70,27.53)	0.776	26.36 (24.14,28.77)	0.963	27.51 (24.73,30.61)	0.915
	>12.300	24.49 (22.32,26.86)	0.483	25.95 (23.89,28.18)	0.681	27.18 (24.60,30.03)	0.931
∑highMWphth	<=9.770	26.11 (23.72,28.74)		26.91 (24.7,29.33)		27.65 (24.94,30.65)	
	>9.770 & <=13.420	24.60 (22.32,27.11)	0.256	26.19 (23.95,28.64)	0.538	27.53 (24.70,30.68)	0.944
	>13.420	24.05 (21.90,26.41)	0.106	25.40 (23.25,27.75)	0.180	26.54 (23.83,29.56)	0.502
∑allphth	<=19.780	25.92 (23.46,28.64)		26.73 (24.31,29.39)		27.47 (24.46,30.86)	
	>19.780 & <=30.900	25.18 (22.94,27.63)	0.558	26.38 (24.20,28.75)	0.758	27.41 (24.70,30.41)	0.970
	>30.900	24.01 (21.89,26.34)	0.142	25.87 (23.83,28.09)	0.461	27.42 (24.80,30.33)	0.977

Models were adjusted for age at measurement, BMI z-score at measurement, maternal education and family income at birth, and length of breastfeeding.

Table S10. Adjusted Associations Between Categorized Phthalate Metabolite Levels and High-Density Lipoprotein Cholesterol (HDL-C, mmol/L)

		14 year followup		17 year followup		20 year followup		22 year followup	
		Marginal Geometric Mean (95%CI)	p	Marginal Geometric Mean (95%CI)	p	Marginal Geometric Mean (95%CI)	p	Marginal Geometric Mean (95%CI)	p
MEP	<=1.750	1.41 (1.32,1.50)		1.26 (1.19,1.35)		1.26 (1.19,1.35)		1.27 (1.19,1.36)	
	>1.750 & <=5.714	1.35 (1.27,1.44)	0.159	1.23 (1.16,1.31)	0.334	1.25 (1.18,1.33)	0.778	1.28 (1.20,1.36)	0.881
	>5.714	1.37 (1.29,1.46)	0.433	1.25 (1.18,1.32)	0.624	1.26 (1.19,1.34)	0.944	1.28 (1.20,1.36)	0.852
MiBP	<=0.792	1.34 (1.26,1.43)		1.22 (1.15,1.30)		1.24 (1.17,1.32)		1.26 (1.18,1.35)	
	>0.792 & <=1.900	1.40 (1.31,1.49)	0.182	1.25 (1.18,1.33)	0.350	1.25 (1.18,1.33)	0.760	1.26 (1.18,1.34)	0.929
	>1.900	1.38 (1.30,1.47)	0.306	1.26 (1.19,1.33)	0.289	1.27 (1.20,1.35)	0.364	1.29 (1.21,1.37)	0.464
MnBP	<=1.999	1.35 (1.27,1.44)		1.23 (1.16,1.31)		1.25 (1.18,1.33)		1.27 (1.19,1.35)	
	>1.999 & <=4.099	1.38 (1.30,1.47)	0.515	1.25 (1.18,1.33)	0.582	1.26 (1.19,1.34)	0.743	1.28 (1.20,1.36)	0.869
	>4.099	1.39 (1.30,1.47)	0.467	1.26 (1.18,1.33)	0.513	1.26 (1.19,1.34)	0.657	1.28 (1.20,1.36)	0.782
MHBP	<=0.220	1.37 (1.30,1.44)		1.24 (1.18,1.31)		1.26 (1.19,1.32)		1.27 (1.21,1.34)	
	>0.220	1.40 (1.31,1.50)	0.377	1.26 (1.19,1.35)	0.485	1.27 (1.19,1.35)	0.722	1.28 (1.19,1.37)	0.905
MBzP	<=0.260	1.37 (1.30,1.45)		1.25 (1.18,1.31)		1.26 (1.19,1.32)		1.27 (1.21,1.34)	
	>0.260	1.38 (1.30,1.47)	0.817	1.25 (1.18,1.33)	0.841	1.26 (1.18,1.34)	0.900	1.28 (1.20,1.36)	0.945
MEHP	<=2.960	1.37 (1.29,1.46)		1.24 (1.17,1.31)		1.24 (1.17,1.31)		1.25 (1.17,1.33)	
	>2.960 & <=5.250	1.34 (1.27,1.43)	0.502	1.23 (1.16,1.31)	0.854	1.25 (1.18,1.33)	0.691	1.28 (1.20,1.36)	0.479
	>5.250	1.42 (1.33,1.51)	0.254	1.29 (1.21,1.37)	0.136	1.29 (1.22,1.38)	0.112	1.31 (1.23,1.40)	0.135
MECPP	<=0.785	1.36 (1.28,1.45)		1.24 (1.17,1.32)		1.26 (1.19,1.34)		1.28 (1.20,1.37)	
	>0.785 & <=1.241	1.38 (1.30,1.47)	0.670	1.25 (1.18,1.33)	0.804	1.26 (1.18,1.34)	0.999	1.27 (1.19,1.36)	0.883
	>1.241	1.38 (1.30,1.46)	0.689	1.25 (1.18,1.32)	0.868	1.25 (1.18,1.33)	0.897	1.27 (1.19,1.35)	0.772
MCMHP	<=1.153	1.36 (1.28,1.45)		1.25 (1.18,1.33)		1.28 (1.20,1.36)		1.31 (1.22,1.40)	
	>1.153 & <=1.810	1.36 (1.28,1.45)	0.906	1.23 (1.16,1.30)	0.463	1.23 (1.16,1.31)	0.200	1.25 (1.17,1.33)	0.139
	>1.810	1.41 (1.32,1.49)	0.303	1.27 (1.20,1.34)	0.657	1.27 (1.20,1.35)	0.770	1.28 (1.20,1.36)	0.482
MCPP	<=0.190	1.40 (1.33,1.49)		1.28 (1.21,1.35)		1.29 (1.22,1.37)		1.31 (1.24,1.39)	
	>0.190	1.35 (1.27,1.43)	0.088	1.22 (1.15,1.29)	**0.021**	1.22 (1.15,1.29)	**0.011**	1.23 (1.16,1.31)	**0.015**
MINP	<=2.363	1.40 (1.32,1.48)		1.27 (1.20,1.34)		1.28 (1.21,1.36)		1.30 (1.22,1.38)	
	>2.363 & <=5.491	1.41 (1.32,1.50)	0.796	1.26 (1.19,1.35)	0.895	1.26 (1.18,1.34)	0.584	1.26 (1.19,1.35)	0.457
	>5.491	1.32 (1.24,1.40)	0.054	1.21 (1.14,1.28)	0.076	1.23 (1.16,1.31)	0.202	1.26 (1.18,1.34)	0.386
MCiOP	<=0.130	1.39 (1.31,1.48)		1.26 (1.19,1.33)		1.26 (1.19,1.34)		1.27 (1.20,1.36)	
	>0.130	1.36 (1.29,1.44)	0.462	1.24 (1.17,1.31)	0.558	1.25 (1.19,1.32)	0.761	1.27 (1.20,1.34)	0.913
MiDP	<=0.720	1.37 (1.30,1.45)		1.24 (1.18,1.31)		1.25 (1.19,1.32)		1.27 (1.20,1.34)	
	>0.720	1.42 (1.31,1.54)	0.378	1.30 (1.20,1.40)	0.185	1.32 (1.22,1.42)	0.112	1.34 (1.24,1.45)	0.109
∑MBP(i+n)	<=2.860	1.35 (1.27,1.44)		1.23 (1.16,1.31)		1.25 (1.17,1.33)		1.27 (1.19,1.36)	
	>2.860 & <=5.986	1.37 (1.29,1.46)	0.620	1.24 (1.17,1.32)	0.779	1.25 (1.18,1.32)	0.986	1.26 (1.18,1.34)	0.850
	>5.986	1.39 (1.31,1.48)	0.328	1.26 (1.19,1.34)	0.364	1.27 (1.20,1.35)	0.512	1.29 (1.21,1.37)	0.657
∑DEHP	<=7.700	1.36 (1.28,1.45)		1.24 (1.16,1.31)		1.25 (1.17,1.33)		1.26 (1.19,1.35)	
	>7.700 & <=11.320	1.36 (1.28,1.44)	0.981	1.23 (1.16,1.31)	0.906	1.24 (1.17,1.31)	0.840	1.26 (1.18,1.33)	0.816
	>11.320	1.41 (1.32,1.50)	0.263	1.28 (1.20,1.36)	0.208	1.29 (1.21,1.37)	0.243	1.31 (1.23,1.39)	0.316
∑DiNP	<=3.700	1.41 (1.33,1.49)		1.27 (1.20,1.35)		1.28 (1.20,1.35)		1.29 (1.21,1.37)	
	>3.700 & <=8.292	1.38 (1.30,1.47)	0.556	1.25 (1.17,1.33)	0.484	1.25 (1.18,1.33)	0.490	1.26 (1.18,1.35)	0.535
	>8.292	1.32 (1.24,1.41)	**0.046**	1.21 (1.14,1.29)	0.074	1.23 (1.16,1.31)	0.217	1.26 (1.18,1.34)	0.428
∑DEHPDiNP	<=9.529	1.38 (1.30,1.47)	0.899	1.25 (1.17,1.32)	0.677	1.24 (1.17,1.32)	0.352	1.25 (1.18,1.34)	0.249
	>9.529 & <=12.850	1.37 (1.28,1.45)	0.660	1.24 (1.16,1.31)	0.768	1.24 (1.17,1.32)	0.941	1.26 (1.18,1.34)	0.948
	>12.850	1.38 (1.30,1.47)	0.913	1.26 (1.19,1.34)	0.581	1.29 (1.21,1.36)	0.235	1.31 (1.23,1.40)	0.147
∑low MWphth	<=5.550	1.37 (1.28,1.47)		1.24 (1.17,1.32)		1.25 (1.17,1.33)		1.26 (1.18,1.35)	
	>5.550 & <=12.300	1.37 (1.29,1.46)	0.989	1.24 (1.17,1.32)	0.934	1.24 (1.17,1.32)	0.886	1.26 (1.18,1.34)	0.868
	>12.300	1.37 (1.30,1.46)	0.964	1.25 (1.18,1.33)	0.707	1.27 (1.20,1.35)	0.500	1.29 (1.22,1.37)	0.431
∑highMWphth	<=9.770	1.38 (1.30,1.47)		1.24 (1.17,1.32)		1.25 (1.17,1.32)		1.26 (1.18,1.34)	
	>9.770 & <=13.420	1.38 (1.29,1.47)	0.938	1.24 (1.17,1.32)	0.918	1.24 (1.17,1.32)	0.910	1.26 (1.18,1.34)	0.914
	>13.420	1.38 (1.29,1.46)	0.925	1.26 (1.19,1.34)	0.623	1.28 (1.21,1.36)	0.288	1.31 (1.23,1.40)	0.193
∑allphth	<=19.780	1.37 (1.28,1.46)		1.23 (1.15,1.31)		1.22 (1.15,1.30)		1.23 (1.15,1.32)	
	>19.780 & <=30.900	1.37 (1.29,1.46)	0.910	1.25 (1.18,1.33)	0.454	1.27 (1.20,1.35)	0.190	1.29 (1.21,1.38)	0.131
	>30.900	1.38 (1.30,1.46)	0.832	1.25 (1.18,1.33)	0.474	1.26 (1.19,1.34)	0.254	1.28 (1.21,1.36)	0.199

Models were adjusted for age at measurement, BMI z-score at measurement, maternal education and family income at birth, and length of breastfeeding.

Table S11. Adjusted Associations Between Categorized Phthalate Metabolite Levels and Low-Density Lipoprotein Cholesterol (LDL-C, mmol/L)

		14 year followup		17 year followup		20 year followup		22 year followup	
		Marginal Mean (95%CI)	p	Marginal Mean (95%CI)	p	Marginal Mean (95%CI)	p	Marginal Mean (95%CI)	p
MEP	<=1.750	2.27 (2.04, 2.49)		2.27 (2.05, 2.49)		2.45 (2.22, 2.68)		2.69 (2.45, 2.93)	
	>1.750 & <=5.714	2.12 (1.91, 2.33)	0.138	2.14 (1.94, 2.35)	0.171	2.34 (2.13, 2.55)	0.296	2.60 (2.38, 2.83)	0.428
	>5.714	2.12 (1.92, 2.32)	0.157	2.14 (1.94, 2.34)	0.167	2.33 (2.13, 2.53)	0.251	2.58 (2.36, 2.80)	0.348
MiBP	<=0.792	2.13 (1.91, 2.34)		2.11 (1.90, 2.32)		2.27 (2.05, 2.48)		2.49 (2.26, 2.72)	
	>0.792 & <=1.900	2.07 (1.85, 2.29)	0.581	2.10 (1.89, 2.31)	0.909	2.30 (2.08, 2.52)	0.737	2.56 (2.32, 2.80)	0.574
	>1.900	2.22 (2.02, 2.42)	0.351	2.25 (2.05, 2.45)	0.126	2.46 (2.26, 2.66)	0.057	2.72 (2.50, 2.93)	**0.046**
MnBP	<=1.999	2.14 (1.93, 2.35)		2.13 (1.93, 2.34)		2.30 (2.09, 2.52)		2.54 (2.30, 2.77)	
	>1.999 & <=4.099	2.05 (1.83, 2.27)	0.377	2.09 (1.88, 2.30)	0.628	2.30 (2.09, 2.52)	0.991	2.57 (2.34, 2.80)	0.802
	>4.099	2.23 (2.02, 2.43)	0.370	2.24 (2.04, 2.44)	0.271	2.42 (2.21, 2.63)	0.257	2.66 (2.45, 2.88)	0.282
MHBP	<=0.220	2.14 (1.96, 2.32)		2.16 (1.98, 2.34)		2.35 (2.17, 2.53)		2.61 (2.42, 2.80)	
	>0.220	2.21 (1.98, 2.44)	0.457	2.21 (1.98, 2.43)	0.551	2.39 (2.15, 2.62)	0.720	2.63 (2.38, 2.88)	0.842
MBzP	<=0.260	2.14 (1.96, 2.33)		2.16 (1.98, 2.35)		2.36 (2.18, 2.55)		2.62 (2.43, 2.81)	
	>0.260	2.17 (1.95, 2.38)	0.783	2.17 (1.96, 2.38)	0.973	2.34 (2.12, 2.56)	0.831	2.58 (2.35, 2.82)	0.734
MEHP	<=2.960	2.15 (1.94, 2.37)		2.19 (1.98, 2.40)		2.40 (2.18, 2.61)		2.66 (2.43, 2.88)	
	>2.960 & <=5.250	2.17 (1.97, 2.38)	0.844	2.18 (1.98, 2.39)	0.960	2.37 (2.16, 2.58)	0.773	2.61 (2.39, 2.84)	0.685
	>5.250	2.12 (1.90, 2.33)	0.710	2.12 (1.91, 2.33)	0.468	2.30 (2.08, 2.51)	0.335	2.54 (2.31, 2.77)	0.303
MECPP	<=0.785	2.15 (1.94, 2.36)		2.18 (1.97, 2.39)	*	2.38 (2.17, 2.60)		2.64 (2.41, 2.87)	
	>0.785 & <=1.241	2.27 (2.06, 2.49)	0.228	2.29 (2.08, 2.50)	0.255	2.48 (2.26, 2.69)	0.376	2.73 (2.50, 2.95)	0.495
	>1.241	2.05 (1.85, 2.26)	0.314	2.06 (1.86, 2.26)	0.197	2.25 (2.04, 2.45)	0.172	2.49 (2.28, 2.71)	0.190
MCMHP	<=1.153	2.16 (1.95, 2.38)		2.16 (1.94, 2.37)		2.33 (2.10, 2.55)		2.56 (2.32, 2.80)	*
	>1.153 & <=1.810	2.16 (1.95, 2.37)	0.950	2.23 (2.02, 2.43)	0.457	2.48 (2.27, 2.68)	0.151	2.77 (2.54, 2.99)	0.084
	>1.810	2.12 (1.91, 2.32)	0.638	2.10 (1.90, 2.30)	0.567	2.27 (2.06, 2.47)	0.554	2.50 (2.28, 2.71)	0.573
MCPP	<=0.190	2.17 (1.97, 2.36)		2.19 (2.00, 2.38)		2.39 (2.20, 2.59)		2.65 (2.45, 2.85)	
	>0.190	2.14 (1.94, 2.34)	0.738	2.14 (1.95, 2.34)	0.521	2.32 (2.12, 2.52)	0.396	2.56 (2.35, 2.77)	0.364
MINP	<=2.363	2.21 (2.01, 2.41)		2.23 (2.04, 2.43)		2.43 (2.22, 2.63)		2.68 (2.46, 2.90)	
	>2.363 & <=5.491	2.05 (1.83, 2.27)	0.099	2.04 (1.83, 2.26)	**0.040**	2.21 (1.99, 2.43)	**0.033**	2.44 (2.21, 2.68)	**0.041**
	>5.491	2.14 (1.93, 2.35)	0.489	2.18 (1.97, 2.38)	0.546	2.39 (2.17, 2.60)	0.671	2.65 (2.42, 2.87)	0.767
MCiOP	<=0.130	2.22 (2.01, 2.42)		2.24 (2.04, 2.44)		2.44 (2.24, 2.65)		2.70 (2.48, 2.93)	
	>0.130	2.10 (1.91, 2.29)	0.195	2.11 (1.92, 2.30)	0.115	2.30 (2.11, 2.49)	0.108	2.55 (2.35, 2.75)	0.130
MiDP	<=0.720	2.15 (1.97, 2.33)		2.17 (1.99, 2.34)		2.35 (2.17, 2.53)		2.60 (2.41, 2.79)	
	>0.720	2.16 (1.89, 2.43)	0.940	2.20 (1.93, 2.46)	0.760	2.41 (2.13, 2.68)	0.624	2.67 (2.38, 2.97)	0.572
∑MBP(i+n)	<=2.860	2.08 (1.87, 2.30)		2.07 (1.86, 2.28)		2.23 (2.01, 2.44)		2.45 (2.22, 2.69)	
	>2.860 & <=5.986	2.09 (1.88, 2.30)	0.912	2.14 (1.93, 2.34)	0.463	2.35 (2.14, 2.56)	0.220	2.62 (2.39, 2.84)	0.157
	>5.986	2.23 (2.03, 2.43)	0.134	2.25 (2.05, 2.45)	0.051	2.44 (2.24, 2.65)	**0.034**	2.69 (2.48, 2.91)	**0.038**
∑DEHP	<=7.700	2.18 (1.96, 2.39)		2.24 (2.02, 2.45)		2.47 (2.25, 2.68)		2.74 (2.51, 2.97)	
	>7.700 & <=11.320	2.15 (1.95, 2.35)	0.758	2.17 (1.97, 2.36)	0.446	2.35 (2.15, 2.56)	0.271	2.60 (2.38, 2.82)	0.222
	>11.320	2.14 (1.92, 2.35)	0.673	2.12 (1.91, 2.33)	0.205	2.27 (2.06, 2.48)	0.055	2.49 (2.26, 2.72)	**0.032**
∑DiNP	<=3.700	2.18 (1.98, 2.39)		2.21 (2.01, 2.41)		2.41 (2.21, 2.62)		2.67 (2.45, 2.89)	
	>3.700 & <=8.292	2.10 (1.88, 2.31)	0.410	2.11 (1.90, 2.32)	0.268	2.29 (2.07, 2.50)	0.221	2.53 (2.30, 2.76)	0.228
	>8.292	2.16 (1.94, 2.37)	0.807	2.17 (1.96, 2.38)	0.683	2.36 (2.15, 2.58)	0.609	2.61 (2.38, 2.84)	0.592
∑DEHPDiNP	<=9.529	2.18 (1.97, 2.39)		2.19 (1.99, 2.40)		2.38 (2.17, 2.59)		2.63 (2.40, 2.86)	
	>9.529 & <=12.850	2.18 (1.96, 2.39)	0.984	2.21 (2.00, 2.42)	0.855	2.41 (2.20, 2.63)	0.750	2.67 (2.45, 2.90)	0.708
	>12.850	2.10 (1.89, 2.31)	0.442	2.10 (1.90, 2.31)	0.347	2.28 (2.07, 2.49)	0.332	2.52 (2.30, 2.75)	0.356
∑low MWphth	<=5.550	2.14 (1.91, 2.36)		2.14 (1.92, 2.36)		2.32 (2.09, 2.55)		2.56 (2.32, 2.80)	
	>5.550 & <=12.300	2.15 (1.93, 2.36)	0.930	2.15 (1.94, 2.36)	0.915	2.33 (2.12, 2.54)	0.913	2.57 (2.34, 2.80)	0.917
	>12.300	2.16 (1.96, 2.36)	0.845	2.19 (1.99, 2.39)	0.598	2.40 (2.20, 2.60)	0.438	2.66 (2.45, 2.87)	0.386
∑highMWphth	<=9.770	2.18 (1.97, 2.39)		2.20 (2.00, 2.41)		2.40 (2.19, 2.62)		2.66 (2.44, 2.88)	
	>9.770 & <=13.420	2.15 (1.94, 2.37)	0.774	2.17 (1.95, 2.38)	0.676	2.35 (2.14, 2.57)	0.629	2.60 (2.37, 2.83)	0.625
	>13.420	2.12 (1.91, 2.33)	0.549	2.13 (1.92, 2.34)	0.425	2.31 (2.10, 2.53)	0.381	2.56 (2.33, 2.79)	0.389
∑allphth	<=19.780	2.18 (1.95, 2.41)		2.16 (1.94, 2.39)		2.32 (2.09, 2.56)		2.55 (2.31, 2.80)	
	>19.780 & <=30.900	2.14 (1.93, 2.35)	0.707	2.17 (1.96, 2.37)	0.964	2.37 (2.16, 2.58)	0.650	2.63 (2.41, 2.86)	0.515
	>30.900	2.14 (1.94, 2.34)	0.720	2.17 (1.97, 2.36)	0.977	2.37 (2.17, 2.57)	0.683	2.63 (2.41, 2.84)	0.551

Models were adjusted for age at measurement, BMI z-score at measurement, maternal education and family income at birth, and length of breastfeeding. * Indicates a significant overall effect of the maternal serum phthalate levels on the outcome. Participants who were not fasting for their blood test or who had triglycerides greater than 4.5 were removed.

Table S12. Adjusted Associations Between Categorized Phthalate Metabolite Levels and Uric Acid (mmol/L)

		14 year followup		20 year followup		22 year followup	
		Marginal Mean (95%CI)	p	Marginal Mean (95%CI)	p	Marginal Mean (95%CI)	p
MEP	<=1.750	0.32 (0.30,0.34)		0.34 (0.32,0.36)		0.37 (0.35,0.40)	
	>1.750 & <=5.714	0.31 (0.29,0.33)	0.374	0.34 (0.32,0.36)	0.858	0.37 (0.35,0.39)	0.948
	>5.714	0.30 (0.28,0.32)	0.035	0.34 (0.32,0.35)	0.548	0.37 (0.35,0.39)	0.963
MiBP	<=0.792	0.31 (0.29,0.33)		0.35 (0.33,0.36)		0.38 (0.36,0.40)	
	>0.792 & <=1.900	0.30 (0.28,0.32)	0.479	0.32 (0.30,0.34)	0.032	0.35 (0.33,0.38)	0.028
	>1.900	0.31 (0.30,0.33)	0.764	0.34 (0.32,0.36)	0.696	0.37 (0.36,0.39)	0.591
MnBP	<=1.999	0.31 (0.29,0.33)		0.34 (0.32,0.36)		0.38 (0.36,0.40)	
	>1.999 & <=4.099	0.32 (0.30,0.34)	0.809	0.34 (0.32,0.36)	0.459	0.37 (0.35,0.39)	0.366
	>4.099	0.30 (0.28,0.32)	0.172	0.34 (0.32,0.35)	0.375	0.37 (0.35,0.39)	0.554
MHBP	<=0.220	0.31 (0.29,0.33)		0.34 (0.32,0.35)		0.37 (0.35,0.39)	
	>0.220	0.31 (0.29,0.33)	0.904	0.33 (0.31,0.36)	0.702	0.37 (0.34,0.39)	0.691
MBzP	<=0.260	0.31 (0.29,0.33)		0.34 (0.32,0.36)		0.37 (0.35,0.39)	
	>0.260	0.30 (0.29,0.32)	0.474	0.33 (0.31,0.35)	0.646	0.37 (0.35,0.39)	0.762
MEHP	<=2.960	0.31 (0.29,0.33)		0.34 (0.32,0.35)		0.37 (0.35,0.39)	
	>2.960 & <=5.250	0.31 (0.29,0.33)	0.983	0.34 (0.32,0.36)	0.960	0.37 (0.35,0.39)	0.960
	>5.250	0.31 (0.29,0.32)	0.598	0.34 (0.32,0.36)	0.613	0.38 (0.35,0.40)	0.466
MECPP	<=0.785	0.31 (0.29,0.33)		0.34 (0.32,0.36)		0.38 (0.35,0.40)	
	>0.785 & <=1.241	0.31 (0.29,0.33)	0.801	0.34 (0.32,0.36)	0.804	0.37 (0.35,0.40)	0.838
	>1.241	0.31 (0.29,0.33)	0.953	0.33 (0.31,0.35)	0.329	0.36 (0.34,0.38)	0.280
MCMHP	<=1.153	0.32 (0.30,0.34)		0.34 (0.32,0.36)		0.38 (0.36,0.40)	
	>1.153 & <=1.810	0.31 (0.29,0.33)	0.398	0.34 (0.32,0.36)	0.692	0.38 (0.36,0.40)	0.847
	>1.810	0.30 (0.29,0.32)	0.174	0.33 (0.31,0.35)	0.109	0.36 (0.34,0.38)	0.160
MCPP	<=0.190	0.31 (0.29,0.33)		0.34 (0.32,0.36)		0.38 (0.36,0.39)	
	>0.190	0.31 (0.29,0.33)	0.861	0.33 (0.31,0.35)	0.227	0.36 (0.34,0.38)	0.189
MINP	<=2.363	0.32 (0.30,0.34)	*	0.34 (0.32,0.35)		0.37 (0.34,0.39)	
	>2.363 & <=5.491	0.29 (0.27,0.31)	0.014	0.33 (0.31,0.35)	0.726	0.37 (0.35,0.39)	0.735
	>5.491	0.31 (0.29,0.33)	0.402	0.34 (0.32,0.36)	0.651	0.37 (0.35,0.40)	0.449
MCiOP	<=0.130	0.31 (0.29,0.33)		0.33 (0.32,0.35)		0.37 (0.35,0.39)	
	>0.130	0.31 (0.29,0.33)	0.607	0.34 (0.32,0.36)	0.600	0.37 (0.35,0.39)	0.663
MiDP	<=0.720	0.31 (0.29,0.32)		0.34 (0.32,0.35)		0.37 (0.35,0.39)	
	>0.720	0.32 (0.30,0.35)	0.190	0.35 (0.33,0.38)	0.100	0.39 (0.36,0.41)	0.143
∑MBP(i+n)	<=2.860	0.32 (0.30,0.34)		0.35 (0.33,0.37)		0.38 (0.36,0.41)	
	>2.860 & <=5.986	0.31 (0.29,0.33)	0.258	0.33 (0.31,0.35)	0.025	0.36 (0.34,0.38)	0.029
	>5.986	0.31 (0.29,0.32)	0.180	0.34 (0.32,0.36)	0.223	0.37 (0.35,0.39)	0.332
∑DEHP	<=7.700	0.32 (0.3,0.34)		0.34 (0.32,0.36)		0.38 (0.36,0.40)	
	>7.700 & <=11.320	0.30 (0.28,0.32)	0.075	0.33 (0.31,0.35)	0.205	0.37 (0.35,0.39)	0.372
	>11.320	0.31 (0.29,0.33)	0.412	0.34 (0.32,0.36)	0.632	0.37 (0.35,0.39)	0.768
∑DiNP	<=3.700	0.32 (0.30,0.33)		0.34 (0.32,0.35)		0.37 (0.35,0.39)	
	>3.700 & <=8.292	0.30 (0.28,0.32)	0.028	0.33 (0.31,0.35)	0.773	0.37 (0.35,0.39)	0.740
	>8.292	0.31 (0.29,0.33)	0.616	0.34 (0.32,0.36)	0.584	0.37 (0.35,0.40)	0.441
∑DEHPDiNP	<=9.529	0.31 (0.29,0.33)		0.33 (0.32,0.35)		0.37 (0.35,0.39)	
	>9.529 & <=12.850	0.30 (0.29,0.32)	0.439	0.33 (0.31,0.35)	0.768	0.37 (0.34,0.39)	0.923
	>12.850	0.31 (0.29,0.33)	0.839	0.34 (0.33,0.36)	0.298	0.38 (0.36,0.40)	0.215
∑low MWphth	<=5.550	0.32 (0.30,0.34)		0.34 (0.32,0.36)		0.37 (0.35,0.40)	
	>5.550 & <=12.300	0.31 (0.29,0.33)	0.142	0.34 (0.32,0.36)	0.621	0.37 (0.35,0.39)	0.904
	>12.300	0.30 (0.29,0.32)	0.092	0.34 (0.32,0.36)	0.648	0.37 (0.35,0.39)	0.990
∑highMWphth	<=9.770	0.31 (0.29,0.33)		0.33 (0.32,0.35)		0.37 (0.35,0.39)	
	>9.770 & <=13.420	0.31 (0.29,0.33)	0.543	0.33 (0.31,0.35)	0.747	0.36 (0.34,0.39)	0.857
	>13.420	0.31 (0.29,0.33)	0.822	0.35 (0.33,0.37)	0.191	0.38 (0.36,0.40)	0.120
∑allphth	<=19.780	0.32 (0.30,0.34)		0.34 (0.32,0.36)		0.37 (0.34,0.39)	
	>19.780 & <=30.900	0.31 (0.29,0.33)	0.115	0.34 (0.32,0.36)	0.679	0.38 (0.36,0.40)	0.351
	>30.900	0.30 (0.29,0.32)	0.067	0.34 (0.32,0.36)	0.959	0.37 (0.35,0.39)	0.546

Models were adjusted for age at measurement, BMI z-score at measurement, maternal education and family income at birth, and length of breastfeeding. * Indicates a significant overall effect of the maternal serum phthalate levels on the outcome.

Table S13. Adjusted Associations Between Categorized Phthalate Metabolite Levels and High Sensitivity C-Reactive Protein (hsCRP, mg/L)

		14 year followup		17 year followup		20 year followup		22 year followup	
		Marginal Geometric Mean (95%CI)	p	Marginal Geometric Mean (95%CI)	p	Marginal Geometric Mean (95%CI)	p	Marginal Geometric Mean (95%CI)	p
MEP	<=1.750	0.48 (0.34, 0.68)		0.43 (0.32, 0.58)		0.61 (0.46, 0.81)		0.69 (0.51, 0.94)	
	>1.750 & <=5.714	0.40 (0.29, 0.55)	0.333	0.37 (0.28, 0.49)	0.223	0.53 (0.41, 0.69)	0.256	0.61 (0.47, 0.79)	0.390
	>5.714	0.40 (0.30, 0.53)	0.293	0.36 (0.28, 0.47)	0.165	0.52 (0.40, 0.66)	0.183	0.59 (0.45, 0.77)	0.292
MiBP	<=0.792	0.54 (0.39, 0.74)	*	0.44 (0.33, 0.58)		0.55 (0.42, 0.73)		0.58 (0.43, 0.78)	
	>0.792 & <=1.900	0.42 (0.31, 0.58)	0.169	0.39 (0.30, 0.52)	0.429	0.58 (0.44, 0.75)	0.745	0.67 (0.49, 0.91)	0.400
	>1.900	0.34 (0.25, 0.44)	**0.003**	0.33 (0.26, 0.43)	**0.019**	0.51 (0.40, 0.65)	0.493	0.61 (0.48, 0.78)	0.697
MnBP	<=1.999	0.43 (0.31, 0.59)		0.40 (0.30, 0.54)		0.59 (0.44, 0.79)		0.69 (0.51, 0.94)	
	>1.999 & <=4.099	0.42 (0.31, 0.58)	0.949	0.36 (0.28, 0.48)	0.373	0.48 (0.38, 0.61)	0.090	0.52 (0.41, 0.67)	0.071
	>4.099	0.39 (0.29, 0.53)	0.627	0.38 (0.29, 0.50)	0.608	0.56 (0.43, 0.73)	0.723	0.67 (0.51, 0.87)	0.841
MHBP	<=0.220	0.40 (0.31, 0.52)		0.37 (0.29, 0.47)		0.52 (0.42, 0.66)		0.59 (0.48, 0.74)	
	>0.220	0.47 (0.33, 0.66)	0.330	0.43 (0.31, 0.59)	0.165	0.62 (0.46, 0.83)	0.138	0.71 (0.52, 0.98)	0.202
MBzP	<=0.260	0.44 (0.33, 0.57)		0.39 (0.31, 0.50)		0.56 (0.44, 0.70)		0.64 (0.50, 0.81)	
	>0.260	0.37 (0.27, 0.50)	0.273	0.34 (0.26, 0.46)	0.195	0.49 (0.38, 0.64)	0.232	0.57 (0.44, 0.74)	0.369
MEHP	<=2.960	0.39 (0.28, 0.54)		0.36 (0.27, 0.48)		0.52 (0.40, 0.68)		0.60 (0.46, 0.79)	
	>2.960 & <=5.250	0.45 (0.34, 0.60)	0.376	0.41 (0.31, 0.54)	0.320	0.57 (0.44, 0.74)	0.422	0.65 (0.50, 0.84)	0.600
	>5.250	0.39 (0.29, 0.54)	0.976	0.36 (0.27, 0.48)	0.963	0.51 (0.39, 0.68)	0.895	0.58 (0.43, 0.79)	0.878
MECPP	<=0.785	0.38 (0.28, 0.53)		0.36 (0.27, 0.48)		0.53 (0.40, 0.69)		0.62 (0.46, 0.83)	
	>0.785 & <=1.241	0.41 (0.31, 0.55)	0.676	0.38 (0.29, 0.50)	0.593	0.56 (0.43, 0.73)	0.646	0.65 (0.48, 0.88)	0.735
	>1.241	0.45 (0.33, 0.62)	0.355	0.39 (0.3, 0.52)	0.489	0.54 (0.41, 0.69)	0.904	0.59 (0.46, 0.76)	0.807
MCMHP	<=1.153	0.39 (0.29, 0.52)		0.37 (0.28, 0.49)		0.55 (0.42, 0.73)		0.65 (0.48, 0.90)	
	>1.153 & <=1.810	0.46 (0.33, 0.65)	0.313	0.41 (0.31, 0.55)	0.422	0.57 (0.44, 0.74)	0.843	0.64 (0.49, 0.83)	0.868
	>1.810	0.41 (0.30, 0.56)	0.763	0.37 (0.28, 0.48)	0.887	0.51 (0.39, 0.66)	0.509	0.57 (0.44, 0.74)	0.407
MCPP	<=0.190	0.41 (0.31, 0.54)		0.39 (0.30, 0.50)		0.57 (0.44, 0.72)		0.66 (0.52, 0.83)	
	>0.190	0.42 (0.32, 0.56)	0.885	0.37 (0.28, 0.49)	0.704	0.51 (0.40, 0.65)	0.325	0.57 (0.43, 0.74)	0.250
MINP	<=2.363	0.32 (0.24, 0.43)	*	0.32 (0.25, 0.41)	*	0.51 (0.40, 0.64)		0.62 (0.49, 0.78)	
	>2.363 & <=5.491	0.49 (0.35, 0.68)	**0.013**	0.46 (0.34, 0.62)	**0.005**	0.66 (0.50, 0.89)	**0.026**	0.77 (0.56, 1.05)	0.148
	>5.491	0.48 (0.35, 0.65)	**0.012**	0.40 (0.30, 0.53)	0.064	0.51 (0.39, 0.67)	0.913	0.54 (0.41, 0.71)	0.331
MCiOP	<=0.130	0.39 (0.28, 0.55)		0.37 (0.28, 0.50)		0.55 (0.42, 0.72)		0.64 (0.48, 0.86)	
	>0.130	0.43 (0.33, 0.55)	0.602	0.39 (0.3, 0.49)	0.793	0.54 (0.42, 0.68)	0.840	0.61 (0.48, 0.76)	0.673
MiDP	<=0.720	0.42 (0.32, 0.54)		0.38 (0.30, 0.49)		0.54 (0.43, 0.68)		0.62 (0.49, 0.77)	
	>0.720	0.39 (0.26, 0.58)	0.727	0.36 (0.26, 0.51)	0.709	0.52 (0.38, 0.72)	0.773	0.60 (0.43, 0.84)	0.859
∑MBP(i+n)	<=2.860	0.48 (0.34, 0.66)		0.42 (0.32, 0.57)		0.59 (0.44, 0.79)		0.66 (0.48, 0.90)	
	>2.860 & <=5.986	0.39 (0.29, 0.53)	0.253	0.35 (0.27, 0.47)	0.147	0.50 (0.39, 0.64)	0.182	0.56 (0.43, 0.73)	0.316
	>5.986	0.40 (0.29, 0.53)	0.280	0.37 (0.29, 0.49)	0.314	0.55 (0.43, 0.70)	0.587	0.64 (0.49, 0.83)	0.849
∑DEHP	<=7.700	0.39 (0.28, 0.53)		0.37 (0.28, 0.48)		0.54 (0.41, 0.70)		0.62 (0.47, 0.82)	
	>7.700 & <=11.320	0.48 (0.36, 0.64)	0.173	0.41 (0.31, 0.54)	0.306	0.55 (0.42, 0.71)	0.868	0.59 (0.45, 0.78)	0.745
	>11.320	0.37 (0.27, 0.50)	0.816	0.35 (0.27, 0.47)	0.766	0.52 (0.40, 0.67)	0.783	0.61 (0.46, 0.79)	0.839
∑DiNP	<=3.700	0.33 (0.25, 0.44)		0.33 (0.26, 0.43)		0.53 (0.41, 0.67)		0.64 (0.50, 0.82)	
	>3.700 & <=8.292	0.46 (0.33, 0.63)	0.053	0.42 (0.32, 0.57)	0.051	0.61 (0.46, 0.81)	0.211	0.70 (0.52, 0.94)	0.552
	>8.292	0.48 (0.35, 0.65)	**0.023**	0.40 (0.30, 0.54)	0.114	0.52 (0.40, 0.68)	0.960	0.55 (0.42, 0.73)	0.303
∑DEHPDiNP	<=9.529	0.36 (0.26, 0.50)		0.36 (0.27, 0.47)		0.55 (0.43, 0.71)		0.66 (0.50, 0.86)	
	>9.529 & <=12.850	0.47 (0.36, 0.62)	0.083	0.41 (0.31, 0.54)	0.226	0.56 (0.43, 0.73)	0.925	0.60 (0.46, 0.80)	0.589
	>12.850	0.42 (0.30, 0.58)	0.359	0.38 (0.28, 0.50)	0.615	0.53 (0.41, 0.69)	0.742	0.59 (0.45, 0.78)	0.450
∑low MWphth	<=5.550	0.48 (0.34, 0.67)		0.42 (0.31, 0.56)		0.57 (0.42, 0.76)		0.63 (0.47, 0.84)	
	>5.550 & <=12.300	0.37 (0.28, 0.51)	0.153	0.36 (0.28, 0.47)	0.250	0.54 (0.42, 0.70)	0.742	0.65 (0.49, 0.85)	0.854
	>12.300	0.41 (0.31, 0.56)	0.376	0.37 (0.28, 0.49)	0.359	0.52 (0.41, 0.67)	0.523	0.59 (0.46, 0.77)	0.716
∑highMWphth	<=9.770	0.38 (0.27, 0.52)		0.37 (0.28, 0.48)		0.56 (0.44, 0.72)		0.67 (0.51, 0.86)	
	>9.770 & <=13.420	0.45 (0.35, 0.60)	0.237	0.40 (0.30, 0.52)	0.516	0.54 (0.41, 0.72)	0.780	0.59 (0.44, 0.79)	0.458
	>13.420	0.42 (0.30, 0.58)	0.538	0.38 (0.28, 0.50)	0.841	0.53 (0.41, 0.69)	0.603	0.59 (0.45, 0.78)	0.401
∑allphth	<=19.780	0.50 (0.36, 0.69)		0.43 (0.32, 0.58)		0.57 (0.43, 0.76)		0.62 (0.46, 0.84)	
	>19.780 & <=30.900	0.35 (0.26, 0.48)	**0.045**	0.35 (0.27, 0.45)	0.085	0.53 (0.41, 0.69)	0.530	0.63 (0.48, 0.83)	0.918
	>30.900	0.42 (0.31, 0.55)	0.297	0.38 (0.29, 0.50)	0.311	0.53 (0.41, 0.68)	0.542	0.60 (0.46, 0.77)	0.779

Models were adjusted for age at measurement, BMI z-score at measurement, maternal education and family income at birth, and length of breastfeeding. * Indicates a significant overall effect of the maternal serum phthalate levels on the outcome. Participants with hsCRP values greater than 10 were removed. Values below the limit of detection (0.15) were set as LOD/ √2

Table S14. Adjusted Associations Between Categorized Phthalate Metabolite Levels and Insulin (mU/L)

		14 year followup		17 year followup		20 year followup		22 year followup	
		Marginal Geometric Mean (95%CI)	p	Marginal Geometric Mean (95%CI)	p	Marginal Geometric Mean (95%CI)	p	Marginal Geometric Mean (95%CI)	p
MEP	<=1.750	8.47 (7.18, 9.99)		5.53 (4.68, 6.52)		3.03 (2.57, 3.57)		4.97 (4.09, 6.04)	
	>1.750 & <=5.714	9.12 (7.60, 10.95)	0.385	5.48 (4.62, 6.51)	0.907	2.76 (2.35, 3.26)	0.224	4.28 (3.57, 5.12)	0.130
	>5.714	8.57 (7.32, 10.03)	0.884	5.43 (4.58, 6.43)	0.784	2.88 (2.39, 3.47)	0.584	4.63 (3.69, 5.82)	0.553
MiBP	<=0.792	9.21 (7.82, 10.84)		5.71 (4.83, 6.75)		2.98 (2.50, 3.56)		4.76 (3.79, 5.96)	
	>0.792 & <=1.900	8.24 (6.82, 9.96)	0.195	5.37 (4.51, 6.40)	0.367	2.95 (2.48, 3.52)	0.911	4.88 (4.00, 5.96)	0.826
	>1.900	8.78 (7.53, 10.23)	0.530	5.37 (4.56, 6.33)	0.304	2.76 (2.34, 3.26)	0.365	4.37 (3.63, 5.25)	0.448
MnBP	<=1.999	8.44 (6.95, 10.26)		5.30 (4.44, 6.31)		2.78 (2.35, 3.30)		4.44 (3.58, 5.50)	
	>1.999 & <=4.099	8.69 (7.45, 10.14)	0.738	5.51 (4.68, 6.48)	0.536	2.92 (2.47, 3.45)	0.546	4.69 (3.89, 5.67)	0.613
	>4.099	8.91 (7.62, 10.43)	0.550	5.56 (4.70, 6.58)	0.482	2.91 (2.42, 3.49)	0.627	4.62 (3.75, 5.69)	0.739
MHBP	<=0.220	8.73 (7.53, 10.12)		5.54 (4.74, 6.48)		2.94 (2.53, 3.41)		4.74 (4.02, 5.58)	
	>0.220	8.38 (7.03, 9.99)	0.586	5.13 (4.29, 6.13)	0.191	2.61 (2.16, 3.16)	0.147	4.10 (3.24, 5.21)	0.183
MBzP	<=0.260	8.65 (7.41, 10.10)		5.51 (4.71, 6.44)		2.95 (2.53, 3.43)		4.79 (4.07, 5.64)	
	>0.260	8.72 (7.42, 10.24)	0.916	5.28 (4.43, 6.29)	0.464	2.68 (2.23, 3.21)	0.220	4.20 (3.36, 5.26)	0.200
MEHP	<=2.960	9.11 (7.62, 10.91)		5.32 (4.53, 6.25)		2.62 (2.22, 3.09)		4.00 (3.34, 4.79)	
	>2.960 & <=5.250	8.93 (7.55, 10.55)	0.808	5.77 (4.85, 6.87)	0.224	3.15 (2.62, 3.79)	0.027	5.19 (4.19, 6.45)	0.020
	>5.250	8.00 (6.88, 9.30)	0.103	5.20 (4.41, 6.13)	0.706	2.86 (2.44, 3.34)	0.261	4.72 (3.86, 5.78)	0.113
MECPP	<=0.785	8.62 (7.24, 10.26)		5.50 (4.64, 6.51)		2.92 (2.48, 3.43)		4.71 (3.83, 5.79)	
	>0.785 & <=1.241	9.53 (7.94, 11.43)	0.256	5.78 (4.81, 6.94)	0.458	2.91 (2.43, 3.49)	0.994	4.54 (3.72, 5.54)	0.743
	>1.241	8.07 (6.92, 9.43)	0.406	5.21 (4.43, 6.12)	0.397	2.80 (2.35, 3.34)	0.626	4.56 (3.72, 5.58)	0.774
MCMHP	<=1.153	9.07 (7.51, 10.95)		5.56 (4.61, 6.72)		2.85 (2.38, 3.42)		4.47 (3.61, 5.53)	
	>1.153 & <=1.810	8.76 (7.43, 10.33)	0.911	5.52 (4.68, 6.51)	0.911	2.91 (2.48, 3.42)	0.800	4.66 (3.89, 5.58)	0.702
	>1.810	8.33 (7.13, 9.73)	0.535	5.34 (4.54, 6.27)	0.535	2.87 (2.40, 3.42)	0.961	4.64 (3.75, 5.73)	0.756
MCPP	<=0.190	8.89 (7.68, 10.29)		5.51 (4.71, 6.44)		2.86 (2.45, 3.34)		4.53 (3.82, 5.39)	
	>0.190	8.46 (7.17, 9.99)	0.465	5.40 (4.57, 6.39)	0.723	2.90 (2.46, 3.42)	0.852	4.70 (3.86, 5.71)	0.703
MINP	<=2.363	8.88 (7.39, 10.66)		5.42 (4.54, 6.46)		2.77 (2.31, 3.31)		4.35 (3.58, 5.29)	
	>2.363 & <=5.491	8.88 (7.57, 10.41)	0.999	5.57 (4.73, 6.55)	0.656	2.93 (2.49, 3.44)	0.458	4.70 (3.88, 5.69)	0.455
	>5.491	8.44 (7.19, 9.91)	0.555	5.43 (4.60, 6.40)	0.973	2.93 (2.46, 3.48)	0.536	4.79 (3.84, 5.97)	0.435
MCiOP	<=0.130	9.39 (7.77, 11.35)		5.66 (4.73, 6.77)		2.88 (2.42, 3.42)		4.47 (3.67, 5.45)	
	>0.130	8.37 (7.27, 9.65)	0.149	5.32 (4.56, 6.21)	0.325	2.86 (2.45, 3.33)	0.926	4.62 (3.88, 5.50)	0.740
MiDP	<=0.720	8.69 (7.54, 10.01)		5.46 (4.69, 6.37)		2.89 (2.49, 3.34)		4.63 (3.92, 5.46)	
	>0.720	8.53 (6.68, 10.89)	0.856	5.28 (4.23, 6.58)	0.659	2.75 (2.19, 3.45)	0.587	4.35 (3.35, 5.65)	0.613
∑MBP(i+n)	<=2.860	8.73 (7.38, 10.32)		5.48 (4.61, 6.51)		2.89 (2.43, 3.43)		4.63 (3.75, 5.71)	
	>2.860 & <=5.986	8.61 (7.17, 10.34)	0.869	5.51 (4.64, 6.55)	0.922	2.97 (2.49, 3.54)	0.750	4.83 (3.93, 5.93)	0.715
	>5.986	8.78 (7.49, 10.29)	0.944	5.38 (4.55, 6.37)	0.791	2.77 (2.33, 3.30)	0.627	4.37 (3.59, 5.32)	0.602
∑DEHP	<=7.700	9.26 (7.54, 11.36)		5.64 (4.71, 6.75)		2.89 (2.41, 3.48)		4.55 (3.74, 5.54)	
	>7.700 & <=11.320	8.53 (7.32, 9.94)	0.357	5.26 (4.46, 6.19)	0.294	2.73 (2.32, 3.21)	0.481	4.32 (3.52, 5.30)	0.646
	>11.320	8.60 (7.32, 10.11)	0.390	5.62 (4.72, 6.68)	0.946	3.09 (2.57, 3.71)	0.418	5.11 (4.10, 6.36)	0.292
∑DiNP	<=3.700	8.90 (7.41, 10.7)		5.42 (4.54, 6.48)		2.76 (2.31, 3.31)		4.35 (3.58, 5.28)	
	>3.700 & <=8.292	8.77 (7.49, 10.26)	0.863	5.55 (4.72, 6.52)	0.733	2.94 (2.50, 3.46)	0.442	4.76 (3.90, 5.81)	0.403
	>8.292	8.51 (7.22, 10.02)	0.601	5.43 (4.61, 6.41)	0.973	2.91 (2.45, 3.46)	0.560	4.74 (3.82, 5.89)	0.460
∑DEHPDiNP	<=9.529	9.11 (7.52, 11.03)		5.51 (4.61, 6.58)		2.78 (2.32, 3.32)		4.36 (3.57, 5.32)	
	>9.529 & <=12.850	8.96 (7.57, 10.60)	0.859	5.53 (4.66, 6.57)	0.949	2.85 (2.39, 3.41)	0.756	4.55 (3.73, 5.54)	0.707
	>12.850	8.19 (7.05, 9.51)	0.207	5.39 (4.57, 6.34)	0.727	2.96 (2.50, 3.51)	0.456	4.94 (3.98, 6.13)	0.277
∑low MWphth	<=5.550	8.96 (7.46, 10.76)		5.55 (4.65, 6.61)		2.86 (2.41, 3.40)		4.52 (3.66, 5.59)	
	>5.550 & <=12.300	8.08 (6.75, 9.67)	0.240	5.22 (4.41, 6.19)	0.365	2.82 (2.39, 3.34)	0.859	4.60 (3.82, 5.55)	0.868
	>12.300	9.02 (7.76, 10.49)	0.932	5.63 (4.77, 6.64)	0.825	2.93 (2.45, 3.51)	0.798	4.65 (3.77, 5.74)	0.810
∑highMWphth	<=9.770	9.17 (7.59, 11.08)		5.51 (4.62, 6.56)		2.76 (2.31, 3.29)		4.30 (3.53, 5.23)	
	>9.770 & <=13.420	8.75 (7.40, 10.35)	0.616	5.50 (4.61, 6.56)	0.975	2.88 (2.40, 3.46)	0.616	4.64 (3.76, 5.72)	0.506
	>13.420	8.25 (7.08, 9.61)	0.215	5.40 (4.59, 6.35)	0.761	2.95 (2.50, 3.49)	0.415	4.90 (3.97, 6.04)	0.245
∑allphth	<=19.780	9.00 (7.32, 11.06)		5.64 (4.67, 6.81)		2.96 (2.46, 3.55)		4.70 (3.77, 5.86)	
	>19.780 & <=30.900	8.26 (7.02, 9.73)	0.337	5.23 (4.45, 6.15)	0.256	2.77 (2.35, 3.26)	0.431	4.44 (3.68, 5.35)	0.601
	>30.900	8.88 (7.64, 10.31)	0.888	5.58 (4.72, 6.59)	0.890	2.93 (2.46, 3.50)	0.936	4.67 (3.79, 5.75)	0.964

Models were adjusted for age at measurement, BMI z-score at measurement, maternal education and family income at birth, and length of breastfeeding. Diabetics (participants with glucose greater than 7) were removed. Values below the limit of detection (2.0) were set as LOD/√2

Table S15. Adjusted Associations Between Categorized Phthalate Metabolite Levels and Glucose (mmol/L)

		14 year followup		17 year followup		20 year followup		22 year followup	
		Marginal Mean (95%CI)	p	Marginal Mean (95%CI)	p	Marginal Mean (95%CI)	p	Marginal Mean (95%CI)	p
MEP	<=1.750	4.77 (4.66, 4.88)		4.78 (4.67, 4.88)		5.00 (4.90, 5.11)		5.04 (4.93, 5.16)	
	>1.750 & <=5.714	4.86 (4.75, 4.96)	0.121	4.85 (4.75, 4.95)	0.103	5.06 (4.96, 5.16)	0.243	5.09 (4.98, 5.20)	0.448
	>5.714	4.76 (4.66, 4.87)	0.916	4.77 (4.67, 4.87)	0.817	4.99 (4.89, 5.08)	0.764	5.03 (4.92, 5.13)	0.769
MiBP	<=0.792	4.87 (4.76, 4.98)		4.85 (4.75, 4.96)		5.05 (4.95, 5.15)		5.07 (4.95, 5.19)	
	>0.792 & <=1.900	4.80 (4.69, 4.91)	0.199	4.79 (4.69, 4.89)	0.172	5.00 (4.90, 5.10)	0.330	5.03 (4.91, 5.15)	0.520
	>1.900	4.74 (4.64, 4.84)	**0.018**	4.77 (4.67, 4.86)	**0.046**	5.01 (4.91, 5.10)	0.362	5.06 (4.96, 5.16)	0.830
MnBP	<=1.999	4.84 (4.74, 4.95)		4.83 (4.73, 4.93)		5.03 (4.93, 5.13)		5.06 (4.94, 5.18)	
	>1.999 & <=4.099	4.78 (4.67, 4.88)	0.230	4.78 (4.68, 4.89)	0.280	5.01 (4.90, 5.11)	0.580	5.05 (4.94, 5.16)	0.836
	>4.099	4.77 (4.67, 4.87)	0.205	4.78 (4.68, 4.88)	0.248	5.00 (4.9,0 5.10)	0.534	5.04 (4.93, 5.15)	0.797
MHBP	<=0.220	4.79 (4.70, 4.88)		4.79 (4.70, 4.88)		5.01 (4.92, 5.09)		5.04 (4.95, 5.13)	
	>0.220	4.84 (4.72, 4.96)	0.344	4.84 (4.73, 4.94)	0.255	5.05 (4.94, 5.16)	0.323	5.09 (4.96, 5.21)	0.445
MBzP	<=0.260	4.82 (4.73, 4.91)		4.80 (4.71, 4.89)		5.00 (4.91, 5.08)		5.01 (4.92, 5.11)	
	>0.260	4.75 (4.64, 4.86)	0.199	4.80 (4.69, 4.90)	0.967	5.07 (4.96, 5.17)	0.102	5.13 (5.02, 5.24)	**0.025**
MEHP	<=2.960	4.79 (4.68, 4.90)		4.79 (4.69, 4.89)		5.00 (4.90, 5.10)		5.03 (4.92, 5.14)	
	>2.960 & <=5.250	4.77 (4.66, 4.87)	0.670	4.78 (4.68, 4.88)	0.932	5.01 (4.91, 5.11)	0.729	5.06 (4.95, 5.17)	0.600
	>5.250	4.84 (4.74, 4.95)	0.325	4.84 (4.74, 4.94)	0.235	5.05 (4.94, 5.15)	0.312	5.08 (4.96, 5.19)	0.440
MECPP	<=0.785	4.82 (4.72, 4.93)		4.82 (4.72, 4.93)		5.04 (4.93, 5.14)		5.07 (4.95, 5.19)	
	>0.785 & <=1.241	4.80 (4.69, 4.90)	0.630	4.79 (4.69, 4.89)	0.441	4.99 (4.89, 5.10)	0.403	5.02 (4.91, 5.14)	0.453
	>1.241	4.77 (4.67, 4.88)	0.347	4.79 (4.69, 4.88)	0.381	5.01 (4.92, 5.11)	0.624	5.06 (4.95, 5.16)	0.826
MCMHP	<=1.153	4.82 (4.71, 4.92)		4.81 (4.70, 4.91)		5.01 (4.91, 5.12)		5.04 (4.93, 5.16)	
	>1.153 & <=1.810	4.76 (4.65, 4.87)	0.351	4.78 (4.68, 4.88)	0.506	5.01 (4.91, 5.11)	0.903	5.06 (4.94, 5.17)	0.863
	>1.810	4.81 (4.70, 4.91)	0.847	4.81 (4.71, 4.90)	0.942	5.02 (4.92, 5.12)	0.926	5.05 (4.95, 5.16)	0.868
MCPP	<=0.190	4.78 (4.68, 4.87)		4.78 (4.69, 4.88)		5.00 (4.91, 5.09)		5.04 (4.94, 5.14)	
	>0.190	4.82 (4.72, 4.92)	0.352	4.82 (4.72, 4.91)	0.340	5.03 (4.93, 5.12)	0.514	5.06 (4.96, 5.17)	0.688
MINP	<=2.363	4.78 (4.67, 4.88)		4.78 (4.68, 4.87)		4.99 (4.89, 5.09)		5.03 (4.92, 5.14)	
	>2.363 & <=5.491	4.84 (4.73, 4.95)	0.282	4.82 (4.71, 4.92)	0.335	5.02 (4.91, 5.12)	0.615	5.04 (4.93, 5.15)	0.850
	>5.491	4.79 (4.69, 4.90)	0.739	4.81 (4.71, 4.92)	0.393	5.05 (4.95, 5.15)	0.239	5.10 (4.99, 5.21)	0.238
MCiOP	<=0.130	4.76 (4.65, 4.87)		4.77 (4.68, 4.87)		5.00 (4.90, 5.10)		5.04 (4.93, 5.16)	
	>0.130	4.82 (4.72, 4.91)	0.258	4.82 (4.72, 4.91)	0.282	5.03 (4.94, 5.12)	0.525	5.06 (4.97, 5.16)	0.753
MiDP	<=0.720	4.80 (4.72, 4.89)		4.80 (4.72, 4.89)		5.02 (4.93, 5.10)		5.05 (4.96, 5.14)	
	>0.720	4.71 (4.57, 4.85)	0.133	4.73 (4.60, 4.86)	0.142	4.96 (4.84, 5.09)	0.339	5.01 (4.87, 5.16)	0.579
∑MBP(i+n)	<=2.860	4.85 (4.74, 4.96)		4.83 (4.73, 4.94)		5.03 (4.93, 5.14)		5.06 (4.94, 5.17)	
	>2.860 & <=5.986	4.78 (4.67, 4.89)	0.226	4.79 (4.69, 4.89)	0.335	5.02 (4.92, 5.11)	0.739	5.06 (4.95, 5.17)	0.976
	>5.986	4.77 (4.67, 4.88)	0.200	4.78 (4.68, 4.88)	0.267	5.01 (4.91, 5.10)	0.591	5.05 (4.94, 5.15)	0.874
∑DEHP	<=7.700	4.79 (4.68, 4.90)		4.79 (4.69, 4.89)		5.00 (4.90, 5.10)		5.03 (4.92, 5.14)	
	>7.700 & <=11.320	4.77 (4.66, 4.87)	0.624	4.77 (4.67, 4.87)	0.701	4.99 (4.90, 5.09)	0.900	5.03 (4.93, 5.14)	0.978
	>11.320	4.84 (4.73, 4.95)	0.388	4.85 (4.74, 4.95)	0.194	5.06 (4.96, 5.17)	0.172	5.10 (4.99, 5.22)	0.229
∑DiNP	<=3.700	4.76 (4.66, 4.87)		4.77 (4.67, 4.87)		4.99 (4.89, 5.09)		5.03 (4.93, 5.14)	
	>3.700 & <=8.292	4.85 (4.74, 4.96)	0.134	4.83 (4.72, 4.93)	0.207	5.02 (4.92, 5.12)	0.577	5.04 (4.93, 5.15)	0.913
	>8.292	4.79 (4.68, 4.90)	0.611	4.81 (4.71, 4.91)	0.373	5.04 (4.94, 5.15)	0.296	5.09 (4.98, 5.21)	0.325
∑DEHPDiNP	<=9.529	4.77 (4.67, 4.88)		4.77 (4.67, 4.87)		4.98 (4.88, 5.08)		5.03 (4.92, 5.13)	*
	>9.529 & <=12.850	4.85 (4.74, 4.96)	0.188	4.81 (4.71, 4.91)	0.393	4.98 (4.88, 5.08)	0.993	5.00 (4.89, 5.11)	0.658
	>12.850	4.79 (4.68, 4.89)	0.763	4.82 (4.73, 4.92)	0.222	5.07 (4.98, 5.17)	0.058	5.14 (5.03, 5.26)	**0.049**
∑low MWphth	<=5.550	4.81 (4.70, 4.92)		4.81 (4.70, 4.91)		5.02 (4.91, 5.12)		5.05 (4.93, 5.17)	
	>5.550 & <=12.300	4.79 (4.69, 4.90)	0.747	4.78 (4.68, 4.88)	0.521	4.98 (4.88, 5.08)	0.428	5.00 (4.89, 5.11)	0.450
	>12.300	4.78 (4.68, 4.88)	0.590	4.81 (4.71, 4.90)	0.966	5.04 (4.95, 5.14)	0.580	5.09 (4.99, 5.20)	0.432
∑highMWphth	<=9.770	4.78 (4.67, 4.88)		4.78 (4.68, 4.88)		5.00 (4.90, 5.10)		5.04 (4.94, 5.15)	
	>9.770 & <=13.420	4.84 (4.73, 4.95)	0.263	4.80 (4.70, 4.91)	0.622	4.98 (4.88, 5.08)	0.677	4.99 (4.88, 5.11)	0.403
	>13.420	4.79 (4.68, 4.89)	0.844	4.82 (4.72, 4.92)	0.406	5.06 (4.96, 5.16)	0.192	5.13 (5.01, 5.24)	0.172
∑allphth	<=19.780	4.79 (4.68, 4.90)		4.80 (4.70, 4.91)		5.03 (4.92, 5.14)		5.08 (4.95, 5.20)	
	>19.780 & <=30.900	4.81 (4.70, 4.91)	0.199	4.78 (4.68, 4.89)	0.273	4.98 (4.88, 5.08)	0.676	5.00 (4.89, 5.11)	0.775
	>30.900	4.79 (4.69, 4.89)	0.909	4.81 (4.71, 4.90)	0.920	5.04 (4.94, 5.13)	0.964	5.08 (4.98, 5.19)	0.989

Models were adjusted for age at measurement, BMI z-score at measurement, maternal education and family income at birth, and length of breastfeeding. * Indicates a significant overall effect of the maternal serum phthalate levels on the outcome. Diabetics (participants with glucose greater than 7), and participants who were not fasting for their blood test were removed.

Table S16. Adjusted Associations Between Categorized Phthalate Metabolite Levels and Homeostatic Model Assessment (HOMA - fasting insulin (microunits per millilitre) × fasting glucose (millimoles per litre)/22.5)

		14 year followup		17 year followup		20 year followup		22 year followup	
		Marginal Geometric Mean (95%CI)	p	Marginal Geometric Mean (95%CI)	p	Marginal Geometric Mean (95%CI)	p	Marginal Geometric Mean (95%CI)	p
MEP	<=1.750	1.78 (1.49, 2.11)		1.16 (0.97, 1.38)		0.67 (0.57, 0.80)		1.10 (0.90, 1.36)	
	>1.750 & <=5.714	1.97 (1.61, 2.40)	0.268	1.17 (0.98, 1.41)	0.877	0.62 (0.52, 0.74)	0.277	0.95 (0.79, 1.14)	0.140
	>5.714	1.82 (1.54, 2.15)	0.775	1.13 (0.95, 1.36)	0.745	0.63 (0.52, 0.76)	0.430	1.00 (0.80, 1.25)	0.376
MiBP	<=0.792	1.98 (1.66, 2.37)		1.20 (1.01, 1.44)		0.65 (0.54, 0.78)		1.02 (0.81, 1.28)	
	>0.792 & <=1.900	1.73 (1.41, 2.13)	0.155	1.13 (0.93, 1.37)	0.372	0.65 (0.54, 0.79)	0.958	1.08 (0.87, 1.33)	0.647
	>1.900	1.85 (1.57, 2.18)	0.407	1.13 (0.95, 1.35)	0.319	0.62 (0.52, 0.73)	0.497	0.97 (0.80, 1.17)	0.639
MnBP	<=1.999	1.77 (1.44, 2.19)		1.11 (0.92, 1.34)		0.62 (0.52, 0.74)		0.99 (0.79, 1.24)	
	>1.999 & <=4.099	1.86 (1.58, 2.19)	0.609	1.17 (0.99, 1.39)	0.453	0.65 (0.55, 0.78)	0.529	1.04 (0.86, 1.27)	0.634
	>4.099	1.91 (1.61, 2.26)	0.467	1.17 (0.98, 1.40)	0.511	0.64 (0.53, 0.76)	0.783	1.00 (0.81, 1.23)	0.947
MHBP	<=0.220	1.84 (1.58, 2.16)		1.16 (0.98, 1.37)		0.65 (0.55, 0.76)		1.04 (0.88, 1.23)	
	>0.220	1.82 (1.50, 2.21)	0.866	1.10 (0.90, 1.33)	0.375	0.59 (0.48, 0.72)	0.220	0.91 (0.71, 1.17)	0.227
MBzP	<=0.260	1.84 (1.55, 2.17)		1.16 (0.98, 1.37)		0.65 (0.55, 0.76)		1.04 (0.88, 1.24)	
	>0.260	1.85 (1.56, 2.21)	0.899	1.12 (0.93, 1.35)	0.613	0.60 (0.50, 0.73)	0.354	0.94 (0.74, 1.19)	0.325
MEHP	<=2.960	1.95 (1.60, 2.37)		1.13 (0.95, 1.34)		0.58 (0.49, 0.69)		0.88 (0.73, 1.06)	
	>2.960 & <=5.250	1.89 (1.58, 2.26)	0.739	1.21 (1.01, 1.45)	0.324	0.69 (0.57, 0.83)	**0.040**	1.13 (0.91, 1.41)	**0.026**
	>5.250	1.71 (1.46, 2.00)	0.138	1.10 (0.93, 1.31)	0.758	0.64 (0.54, 0.75)	0.249	1.05 (0.85, 1.30)	0.108
MECPP	<=0.785	1.83 (1.51, 2.22)	*	1.16 (0.97, 1.40)		0.65 (0.55, 0.77)		1.04 (0.84, 1.29)	
	>0.785 & <=1.241	2.04 (1.68, 2.47)	0.270	1.22 (1.01, 1.48)	0.511	0.64 (0.53, 0.78)	0.903	0.99 (0.81, 1.22)	0.648
	>1.241	1.69 (1.43, 2.00)	0.344	1.09 (0.92, 1.29)	0.300	0.61 (0.51, 0.73)	0.502	0.99 (0.81, 1.22)	0.666
MCMHP	<=1.153	1.94 (1.59, 2.37)		1.18 (0.97, 1.44)		0.63 (0.52, 0.77)		0.98 (0.79, 1.23)	
	>1.153 & <=1.810	1.89 (1.58, 2.26)	0.791	1.18 (0.99, 1.40)	0.982	0.65 (0.55, 0.77)	0.799	1.02 (0.85, 1.24)	0.729
	>1.810	1.73 (1.46, 2.04)	0.178	1.11 (0.93, 1.32)	0.369	0.63 (0.53, 0.75)	0.933	1.02 (0.82, 1.26)	0.793
MCPP	<=0.190	1.88 (1.60, 2.19)		1.16 (0.98, 1.37)		0.64 (0.54, 0.75)		1.01 (0.84, 1.20)	
	>0.190	1.81 (1.51, 2.17)	0.618	1.14 (0.95, 1.36)	0.732	0.64 (0.54, 0.76)	0.989	1.02 (0.84, 1.24)	0.898
MINP	<=2.363	1.88 (1.54, 2.28)		1.14 (0.95, 1.38)		0.62 (0.51, 0.74)		0.97 (0.79, 1.18)	
	>2.363 & <=5.491	1.86 (1.56, 2.21)	0.908	1.17 (0.98, 1.39)	0.744	0.65 (0.55, 0.77)	0.470	1.05 (0.86, 1.28)	0.440
	>5.491	1.82 (1.53, 2.17)	0.756	1.15 (0.96, 1.37)	0.949	0.64 (0.53, 0.77)	0.677	1.03 (0.82, 1.29)	0.608
MCiOP	<=0.130	1.95 (1.59, 2.40)		1.18 (0.98, 1.44)		0.64 (0.53, 0.77)		1.00 (0.81, 1.23)	
	>0.130	1.80 (1.54, 2.09)	0.344	1.13 (0.96, 1.33)	0.463	0.63 (0.54, 0.74)	0.845	1.01 (0.84, 1.20)	0.922
MiDP	<=0.720	1.85 (1.59, 2.15)		1.15 (0.98, 1.36)		0.64 (0.55, 0.74)		1.01 (0.85, 1.20)	
	>0.720	1.76 (1.36, 2.29)	0.670	1.10 (0.87, 1.38)	0.539	0.60 (0.48, 0.77)	0.574	0.96 (0.73, 1.26)	0.654
∑MBP(i+n)	<=2.860	1.83 (1.52, 2.19)		1.15 (0.96, 1.38)		0.64 (0.54, 0.77)		1.03 (0.83, 1.28)	
	>2.860 & <=5.986	1.83 (1.50, 2.23)	0.998	1.15 (0.96, 1.39)	0.941	0.65 (0.54, 0.78)	0.906	1.04 (0.85, 1.28)	0.905
	>5.986	1.89 (1.59, 2.23)	0.717	1.15 (0.96, 1.37)	0.970	0.62 (0.52, 0.74)	0.662	0.97 (0.79, 1.19)	0.581
∑DEHP	<=7.700	1.99 (1.59, 2.49)		1.20 (0.98, 1.46)		0.64 (0.53, 0.78)		1.00 (0.82, 1.23)	
	>7.700 & <=11.320	1.81 (1.54, 2.13)	0.321	1.11 (0.93, 1.31)	0.264	0.60 (0.51, 0.72)	0.450	0.95 (0.77, 1.17)	0.628
	>11.320	1.82 (1.53, 2.17)	0.351	1.18 (0.98, 1.42)	0.841	0.68 (0.57, 0.82)	0.448	1.13 (0.91, 1.40)	0.289
∑DiNP	<=3.700	1.87 (1.53, 2.28)		1.14 (0.94, 1.38)		0.62 (0.51, 0.74)		0.97 (0.80, 1.18)	
	>3.700 & <=8.292	1.85 (1.57, 2.19)	0.938	1.17 (0.98, 1.39)	0.719	0.66 (0.55, 0.78)	0.465	1.06 (0.86, 1.30)	0.437
	>8.292	1.84 (1.54, 2.19)	0.867	1.15 (0.96, 1.37)	0.926	0.64 (0.53, 0.76)	0.737	1.02 (0.82, 1.27)	0.692
∑DEHPDiNP	<=9.529	1.92 (1.56, 2.36)		1.16 (0.96, 1.40)		0.62 (0.51, 0.74)		0.97 (0.79, 1.19)	
	>9.529 & <=12.850	1.96 (1.63, 2.34)	0.844	1.19 (0.99, 1.42)	0.763	0.63 (0.53, 0.76)	0.767	1.00 (0.81, 1.22)	0.807
	>12.850	1.72 (1.46, 2.01)	0.228	1.13 (0.95, 1.34)	0.680	0.65 (0.55, 0.78)	0.522	1.09 (0.87, 1.36)	0.320
∑low MWphth	<=5.550	1.88 (1.54, 2.29)		1.17 (0.97, 1.41)		0.64 (0.53, 0.77)		1.01 (0.81, 1.26)	
	>5.550 & <=12.300	1.73 (1.42, 2.09)	0.379	1.10 (0.92, 1.32)	0.419	0.62 (0.52, 0.74)	0.724	1.00 (0.82, 1.22)	0.929
	>12.300	1.93 (1.64, 2.26)	0.776	1.19 (1.00, 1.42)	0.770	0.65 (0.54, 0.78)	0.864	1.02 (0.83, 1.26)	0.922
∑highMWphth	<=9.770	1.93 (1.57, 2.37)		1.16 (0.96, 1.40)		0.61 (0.51, 0.74)		0.96 (0.78, 1.17)	
	>9.770 & <=13.420	1.90 (1.59, 2.28)	0.897	1.18 (0.97, 1.42)	0.876	0.64 (0.53, 0.77)	0.673	1.01 (0.81, 1.26)	0.630
	>13.420	1.73 (1.47, 2.04)	0.241	1.13 (0.95, 1.34)	0.685	0.65 (0.55, 0.77)	0.518	1.07 (0.87, 1.33)	0.315
∑allphth	<=19.780	1.90 (1.52, 2.37)		1.19 (0.97, 1.46)		0.66 (0.54, 0.80)		1.05 (0.83, 1.32)	
	>19.780 & <=30.900	1.76 (1.48, 2.09)	0.425	1.10 (0.93, 1.31)	0.271	0.61 (0.51, 0.73)	0.351	0.97 (0.79, 1.18)	0.476
	>30.900	1.90 (1.61, 2.23)	0.997	1.18 (0.99, 1.41)	0.906	0.65 (0.54, 0.77)	0.846	1.02 (0.83, 1.26)	0.839

Models were adjusted for age at measurement, BMI z-score at measurement, maternal education and family income at birth, and length of breastfeeding. * Indicates a significant overall effect of the maternal serum phthalate levels on the outcome. Diabetics (participants with glucose greater than 7), and participants who were not fasting for their blood test were removed. Values of insulin below the limit of detection (2.0) were set as LOD/ √2

Table S17. Adjusted Associations Between Categorized Phthalate Metabolite Levels and Creatinine (umol/L)

		14 year followup		20 year followup		22 year followup	
		Marginal Mean (95%CI)	p	Marginal Mean (95%CI)	p	Marginal Mean (95%CI)	p
MEP	<=1.750	66.35 (63.12,69.57)		83.18 (80.03,86.33)		84.08 (80.69,87.47)	
	>1.750 & <=5.714	63.70 (60.69,66.70)	0.077	81.56 (78.63,84.48)	0.247	82.82 (79.65,85.99)	0.456
	>5.714	64.46 (61.55,67.36)	0.220	83.86 (80.97,86.74)	0.640	85.68 (82.51,88.84)	0.356
MiBP	<=0.792	63.87 (60.71,67.02)		81.67 (78.59,84.75)		82.84 (79.48,86.19)	
	>0.792 & <=1.900	65.31 (62.22,68.40)	0.352	82.96 (79.89,86.02)	0.386	84.07 (80.65,87.48)	0.494
	>1.900	64.55 (61.67,67.44)	0.642	83.19 (80.36,86.03)	0.271	84.66 (81.64,87.68)	0.271
MnBP	<=1.999	64.41 (61.33,67.48)		82.45 (79.41,85.50)		83.78 (80.39,87.18)	
	>1.999 & <=4.099	66.80 (63.69,69.91)	0.114	84.38 (81.39,87.36)	0.177	85.54 (82.32,88.75)	0.311
	>4.099	63.53 (60.64,66.42)	0.563	81.99 (79.11,84.87)	0.747	83.47 (80.36,86.58)	0.857
MHBP	<=0.220	64.71 (62.13,67.29)		82.96 (80.40,85.52)		84.35 (81.66,87.04)	
	>0.220	64.19 (60.80,67.58)	0.717	81.68 (78.37,84.98)	0.336	82.79 (79.20,86.39)	0.330
MBzP	<=0.260	65.16 (62.51,67.81)		83.01 (80.41,85.61)		84.26 (81.50,87.02)	
	>0.260	63.60 (60.54,66.66)	0.238	82.07 (78.99,85.15)	0.450	83.55 (80.24,86.85)	0.633
MEHP	<=2.960	64.94 (61.91,67.98)		82.59 (79.61,85.56)		83.74 (80.57,86.92)	
	>2.960 & <=5.250	64.96 (61.98,67.93)	0.993	83.69 (80.73,86.65)	0.437	85.24 (82.00,88.47)	0.379
	>5.250	63.91 (60.79,67.03)	0.492	81.71 (78.67,84.75)	0.535	82.92 (79.59,86.26)	0.630
MECPP	<=0.785	63.88 (60.84,66.92)		81.20 (78.22,84.19)		82.19 (78.88,85.51)	
	>0.785 & <=1.241	64.11 (61.03,67.20)	0.880	82.61 (79.59,85.63)	0.332	84.02 (80.76,87.29)	0.297
	>1.241	65.86 (62.9,68.81)	0.180	84.13 (81.25,87.00)	0.033	85.46 (82.38,88.54)	0.050
MCMHP	<=1.153	63.23 (60.10,66.35)		80.07 (76.94,83.20)	*	80.89 (77.50,84.29)	*
	>1.153 & <=1.810	65.90 (62.88,68.93)	0.081	82.86 (79.99,85.73)	0.050	83.72 (80.60,86.85)	0.098
	>1.810	64.28 (61.33,67.24)	0.480	84.17 (81.29,87.05)	0.003	86.09 (83.02,89.17)	0.002
MCPP	<=0.190	64.10 (61.33,66.87)		82.38 (79.61,85.15)		83.77 (80.86,86.68)	
	>0.190	65.27 (62.44,68.10)	0.349	83.00 (80.24,85.77)	0.594	84.20 (81.20,87.20)	0.761
MINP	<=2.363	64.32 (61.43,67.21)		82.12 (79.19,85.06)		83.39 (80.20,86.57)	
	>2.363 & <=5.491	63.31 (60.10,66.53)	0.510	82.04 (78.95,85.13)	0.953	83.63 (80.30,86.96)	0.886
	>5.491	66.10 (63.06,69.14)	0.230	83.71 (80.74,86.68)	0.267	84.90 (81.66,88.15)	0.377
MCiOP	<=0.130	63.21 (60.21,66.22)		81.81 (78.88,84.75)		83.29 (80.04,86.53)	
	>0.130	65.54 (62.85,68.22)	0.083	83.40 (80.72,86.08)	0.209	84.61 (81.80,87.41)	0.386
MiDP	<=0.720	64.40 (61.87,66.93)		82.82 (80.28,85.36)		84.31 (81.65,86.97)	
	>0.720	66.79 (62.83,70.75)	0.156	82.79 (78.97,86.61)	0.987	83.41 (79.23,87.60)	0.636
∑MBP(i+n)	<=2.860	65.51 (62.32,68.69)		82.82 (79.71,85.93)		83.83 (80.41,87.24)	
	>2.860 & <=5.986	64.34 (61.31,67.37)	0.437	82.38 (79.42,85.35)	0.759	83.65 (80.43,86.88)	0.920
	>5.986	64.34 (61.41,67.26)	0.449	83.08 (80.18,85.98)	0.858	84.60 (81.48,87.72)	0.656
∑DEHP	<=7.700	65.32 (62.17,68.47)		81.43 (78.33,84.53)		82.11 (78.79,85.44)	
	>7.700 & <=11.320	63.92 (61.00,66.84)	0.356	83.88 (81.03,86.72)	0.083	85.94 (82.84,89.04)	0.024
	>11.320	64.67 (61.59,67.75)	0.667	82.45 (79.42,85.48)	0.476	83.74 (80.43,87.04)	0.346
∑DiNP	<=3.700	64.10 (61.18,67.01)		82.49 (79.56,85.41)		83.92 (80.76,87.09)	
	>3.700 & <=8.292	64.15 (61.00,67.30)	0.973	82.10 (79.04,85.17)	0.790	83.38 (80.06,86.71)	0.755
	>8.292	65.86 (62.78,68.93)	0.243	83.69 (80.70,86.68)	0.401	84.93 (81.66,88.20)	0.559
∑DEHPDiNP	<=9.529	63.56 (60.54,66.58)		82.24 (79.21,85.26)		83.73 (80.47,86.99)	
	>9.529 & <=12.850	65.45 (62.31,68.59)	0.223	83.43 (80.43,86.44)	0.410	84.68 (81.43,87.92)	0.587
	>12.850	65.07 (62.08,68.06)	0.313	82.68 (79.73,85.63)	0.758	83.79 (80.53,87.04)	0.974
∑low MWphth	<=5.550	65.95 (62.72,69.18)		83.17 (79.97,86.36)		84.17 (80.71,87.64)	
	>5.550 & <=12.300	64.40 (61.33,67.46)	0.312	82.00 (79.02,84.99)	0.424	83.15 (79.88,86.42)	0.560
	>12.300	64.06 (61.19,66.94)	0.210	83.20 (80.37,86.02)	0.983	84.89 (81.85,87.94)	0.674
∑highMWphth	<=9.770	63.87 (60.88,66.86)		82.15 (79.16,85.14)		83.52 (80.30,86.74)	
	>9.770 & <=13.420	64.80 (61.68,67.92)	0.548	83.34 (80.31,86.37)	0.414	84.80 (81.52,88.09)	0.463
	>13.420	65.39 (62.35,68.42)	0.317	82.83 (79.85,85.80)	0.637	83.89 (80.63,87.16)	0.827
∑allphth	<=19.780	65.12 (61.85,68.38)		83.14 (79.89,86.40)		84.40 (80.86,87.94)	
	>19.780 & <=30.900	64.98 (61.94,68.02)	0.926	81.88 (78.93,84.83)	0.382	82.74 (79.52,85.95)	0.340
	>30.900	64.00 (61.11,66.88)	0.469	83.36 (80.53,86.18)	0.884	85.09 (82.03,88.16)	0.695

Models were adjusted for age at measurement, BMI z-score at measurement, maternal education and family income at birth, and length of breastfeeding. * Indicates a significant overall effect of the maternal serum phthalate levels on the outcome.

Table S18. Adjusted Associations Between Categorized Phthalate Metabolite Levels and Triglycerides (TG, mmol/L)

		14 year followup		17 year followup		20 year followup		22 year followup	
		Geometric Marginal Mean (95%CI)	p	Geometric Marginal Mean (95%CI)	p	Geometric Marginal Mean (95%CI)	p	Geometric Marginal Mean (95%CI)	p
MEP	<=1.750	0.85 (0.74,0.97)		0.90 (0.80,1.03)		0.99 (0.87,1.13)		1.08 (0.93,1.24)	
	>1.750 & <=5.714	0.83 (0.73,0.95)	0.770	0.87 (0.77,0.98)	0.422	0.92 (0.81,1.04)	0.241	0.98 (0.86,1.13)	0.211
	>5.714	0.91 (0.80,1.03)	0.273	0.94 (0.84,1.05)	0.498	0.99 (0.88,1.12)	0.949	1.05 (0.92,1.20)	0.779
MiBP	<=0.792	0.82 (0.72,0.94)		0.86 (0.76,0.98)		0.93 (0.82,1.06)		1.00 (0.87,1.16)	
	>0.792 & <=1.900	0.89 (0.78,1.01)	0.240	0.93 (0.82,1.05)	0.219	0.99 (0.87,1.13)	0.330	1.06 (0.92,1.22)	0.463
	>1.900	0.88 (0.78,1.00)	0.273	0.92 (0.82,1.03)	0.275	0.98 (0.87,1.10)	0.405	1.05 (0.92,1.19)	0.546
MnBP	<=1.999	0.82 (0.72,0.94)		0.85 (0.75,0.96)		0.89 (0.79,1.01)	*	0.95 (0.82,1.09)	*
	>1.999 & <=4.099	0.89 (0.78,1.01)	0.244	0.95 (0.84,1.08)	**0.037**	1.04 (0.92,1.18)	**0.011**	1.14 (0.99,1.30)	**0.011**
	>4.099	0.89 (0.79,1.01)	0.226	0.92 (0.82,1.04)	0.131	0.98 (0.86,1.10)	0.142	1.04 (0.91,1.18)	0.198
MHBP	<=0.220	0.87 (0.78,0.97)		0.91 (0.82,1.01)		0.98 (0.88,1.09)		1.05 (0.94,1.17)	
	>0.220	0.84 (0.73,0.97)	0.578	0.88 (0.77,1.01)	0.506	0.94 (0.82,1.08)	0.530	1.01 (0.87,1.17)	0.588
MBzP	<=0.260	0.85 (0.76,0.95)		0.89 (0.80,0.99)		0.96 (0.86,1.07)		1.03 (0.92,1.15)	
	>0.260	0.91 (0.80,1.04)	0.186	0.95 (0.84,1.07)	0.204	1.00 (0.88,1.14)	0.360	1.07 (0.93,1.23)	0.533
MEHP	<=2.960	0.92 (0.81,1.04)	*	0.95 (0.84,1.06)	*	0.99 (0.88,1.12)	*	1.06 (0.93,1.20)	
	>2.960 & <=5.250	0.88 (0.78,1.00)	0.522	0.94 (0.84,1.06)	0.917	1.02 (0.91,1.16)	0.610	1.12 (0.98,1.27)	0.426
	>5.250	0.78 (0.69,0.89)	**0.013**	0.82 (0.73,0.93)	**0.008**	0.88 (0.77,0.99)	**0.032**	0.94 (0.82,1.08)	0.102
MECPP	<=0.785	0.84 (0.74,0.96)		0.89 (0.79,1.01)		0.97 (0.86,1.10)		1.06 (0.92,1.21)	
	>0.785 & <=1.241	0.93 (0.82,1.06)	0.106	0.96 (0.85,1.09)	0.183	1.01 (0.89,1.15)	0.493	1.08 (0.94,1.24)	0.790
	>1.241	0.83 (0.73,0.94)	0.875	0.87 (0.77,0.98)	0.646	0.93 (0.83,1.05)	0.500	1.00 (0.88,1.14)	0.472
MCMHP	<=1.153	0.89 (0.78,1.01)		0.92 (0.82,1.05)		0.98 (0.86,1.12)		1.06 (0.91,1.22)	
	>1.153 & <=1.810	0.90 (0.79,1.02)	0.824	0.93 (0.83,1.05)	0.857	0.99 (0.88,1.12)	0.928	1.06 (0.93,1.21)	0.974
	>1.810	0.83 (0.73,0.94)	0.257	0.87 (0.78,0.98)	0.280	0.94 (0.83,1.06)	0.443	1.02 (0.89,1.16)	0.605
MCPP	<=0.190	0.87 (0.77,0.98)		0.91 (0.82,1.02)		0.98 (0.88,1.10)		1.06 (0.94,1.19)	
	>0.190	0.86 (0.77,0.98)	0.911	0.90 (0.80,1.01)	0.716	0.95 (0.85,1.07)	0.581	1.02 (0.90,1.16)	0.551
MINP	<=2.363	0.88 (0.78,1.00)		0.93 (0.83,1.05)	*	1.01 (0.89,1.14)		1.09 (0.96,1.24)	
	>2.363 & <=5.491	0.79 (0.69,0.90)	0.080	0.82 (0.73,0.94)	**0.027**	0.88 (0.78,1.00)	**0.029**	0.95 (0.83,1.09)	0.053
	>5.491	0.90 (0.79,1.02)	0.816	0.93 (0.82,1.05)	0.944	0.98 (0.87,1.11)	0.703	1.05 (0.92,1.20)	0.610
MCiOP	<=0.130	0.90 (0.80,1.02)		0.94 (0.84,1.06)		1.00 (0.89,1.13)		1.08 (0.94,1.23)	
	>0.130	0.84 (0.75,0.94)	0.218	0.88 (0.79,0.98)	0.177	0.94 (0.84,1.06)	0.253	1.01 (0.90,1.14)	0.363
MiDP	<=0.720	0.86 (0.77,0.95)		0.90 (0.81,1.00)		0.97 (0.88,1.08)		1.05 (0.94,1.18)	
	>0.720	0.98 (0.83,1.16)	0.058	0.98 (0.84,1.15)	0.169	1.01 (0.86,1.18)	0.615	1.05 (0.88,1.26)	0.979
∑MBP(i+n)	<=2.860	0.81 (0.71,0.92)		0.84 (0.74,0.95)		0.89 (0.78,1.01)	*	0.95 (0.82,1.09)	
	>2.860 & <=5.986	0.90 (0.79,1.02)	0.094	0.95 (0.85,1.07)	**0.017**	1.03 (0.91,1.17)	**0.011**	1.12 (0.98,1.28)	**0.018**
	>5.986	0.88 (0.78,1.00)	0.176	0.91 (0.81,1.03)	0.119	0.97 (0.86,1.09)	0.157	1.03 (0.91,1.18)	0.235
∑DEHP	<=7.700	0.91 (0.80,1.04)	*	0.96 (0.85,1.08)		1.02 (0.90,1.16)		1.10 (0.96,1.26)	
	>7.700 & <=11.320	0.90 (0.80,1.02)	0.836	0.92 (0.82,1.03)	0.450	0.95 (0.85,1.08)	0.240	1.01 (0.88,1.15)	0.200
	>11.320	0.79 (0.69,0.90)	**0.022**	0.85 (0.75,0.96)	**0.030**	0.93 (0.82,1.06)	0.129	1.02 (0.89,1.17)	0.314
∑DiNP	<=3.700	0.89 (0.78,1.01)		0.93 (0.83,1.05)		1.00 (0.89,1.13)		1.08 (0.95,1.23)	
	>3.700 & <=8.292	0.80 (0.70,0.91)	0.098	0.84 (0.74,0.95)	**0.049**	0.90 (0.79,1.02)	0.065	0.97 (0.84,1.11)	0.114
	>8.292	0.90 (0.79,1.02)	0.886	0.93 (0.83,1.05)	0.981	0.99 (0.87,1.12)	0.842	1.06 (0.93,1.21)	0.783
∑DEHPDiNP	<=9.529	0.90 (0.79,1.02)		0.93 (0.83,1.05)	*	0.99 (0.87,1.12)	*	1.05 (0.92,1.21)	
	>9.529 & <=12.850	0.90 (0.79,1.03)	0.986	0.95 (0.84,1.08)	0.713	1.03 (0.91,1.17)	0.480	1.12 (0.98,1.28)	0.413
	>12.850	0.80 (0.71,0.91)	0.068	0.84 (0.74,0.94)	**0.044**	0.89 (0.79,1.01)	0.082	0.96 (0.84,1.09)	0.160
∑low MWphth	<=5.550	0.83 (0.73,0.96)		0.87 (0.77,0.99)		0.94 (0.82,1.07)		1.01 (0.87,1.16)	
	>5.550 & <=12.300	0.85 (0.75,0.97)	0.711	0.90 (0.80,1.02)	0.578	0.97 (0.86,1.10)	0.536	1.05 (0.92,1.21)	0.556
	>12.300	0.89 (0.79,1.01)	0.297	0.92 (0.82,1.04)	0.311	0.98 (0.87,1.10)	0.459	1.05 (0.92,1.19)	0.605
∑highMWphth	<=9.770	0.89 (0.79,1.01)		0.93 (0.83,1.04)		0.99 (0.87,1.11)		1.05 (0.92,1.20)	
	>9.770 & <=13.420	0.89 (0.78,1.02)	0.959	0.95 (0.84,1.07)	0.757	1.02 (0.90,1.16)	0.527	1.11 (0.97,1.27)	0.457
	>13.420	0.81 (0.71,0.92)	0.104	0.84 (0.75,0.95)	0.070	0.90 (0.79,1.02)	0.113	0.96 (0.84,1.10)	0.197
∑allphth	<=19.780	0.86 (0.75,0.99)		0.90 (0.79,1.03)		0.97 (0.85,1.11)		1.05 (0.9,1.21)	
	>19.780 & <=30.900	0.86 (0.76,0.98)	0.993	0.91 (0.81,1.03)	0.837	0.99 (0.88,1.12)	0.713	1.08 (0.94,1.23)	0.677
	>30.900	0.88 (0.78,1.00)	0.681	0.91 (0.81,1.01)	0.962	0.95 (0.84,1.07)	0.722	1.01 (0.88,1.14)	0.589

Models were adjusted for age at measurement, BMI z-score at measurement, maternal education and family income at birth, and length of breastfeeding. * Indicates a significant overall effect of the maternal serum phthalate levels on the outcome. Participants who were not fasting for their blood test were removed.

Table S19. Adjusted Associations Between Categorized Phthalate Metabolite Levels and Fat Mass (g)

		27 year follow-up		Change from 20-27	
		Marginal Geometric Mean (95%CI)	p	Marginal Geometric Mean (95%CI)	p
MEP	<=1.750	17416 (14601, 20772)		3442 (2989, 3957)	
	>1.750 & <=5.714	18643 (15773, 22034)	0.412	4470 (3836, 5208)	0.497
	>5.714	18237 (15577, 21352)	0.591	4277 (3701, 4942)	0.561
MiBP	<=0.792	17110 (14424, 20296)		3510 (3013, 4087)	
	>0.792 & <=1.900	18226 (15464, 21481)	0.454	3458 (3040, 3928)	0.818
	>1.900	18783 (16010, 22036)	0.257	4933 (4264, 5706)	0.338
MnBP	<=1.999	17574 (14860, 20784)		3778 (3277, 4353)	
	>1.999 & <=4.099	19540 (16541, 23082)	0.200	4029 (3455, 4697)	0.893
	>4.099	17506 (14949, 20500)	0.962	4341 (3785, 4977)	0.590
MHBP	<=0.220	18325 (15924, 21087)		3906 (3424, 4456)	
	>0.220	17376 (14537, 20768)	0.494	4342 (3684, 5118)	0.529
MBzP	<=0.260	18210 (15820, 20962)		3862 (3382, 4409)	
	>0.260	17752 (14930, 21108)	0.729	4508 (3887, 5223)	0.464
MEHP	<=2.960	19125 (16284, 22461)		4744 (4131, 5444)	
	>2.960 & <=5.250	17803 (15091, 21002)	0.392	4497 (3834, 5275)	0.941
	>5.250	17400 (14701, 20596)	0.242	2959 (2572, 3401)	0.219
MECPP	<=0.785	18217 (15402, 21547)		2850 (2487, 3263)	
	>0.785 & <=1.241	18838 (15846, 22394)	0.703	5494 (4613, 6544)	0.039
	>1.241	17790 (15184, 20842)	0.772	3957 (3443, 4546)	0.295
MCMHP	<=1.153	17393 (14618, 20694)		2985 (2577, 3454)	
	>1.153 & <=1.810	19743 (16850, 23132)	0.135	5674 (4906, 6560)	0.068
	>1.810	17122 (14582, 20105)	0.848	3507 (3085, 3982)	0.610
MCPP	<=0.190	18650 (16042, 21682)		3544 (3079, 4079)	
	>0.190	17646 (15173, 20521)	0.415	4621 (4014, 5319)	0.161
MINP	<=2.363	18057 (15249, 21383)		3967 (3358, 4686)	*
	>2.363 & <=5.491	18032 (15283, 21276)	0.987	2545 (2278, 2833)	0.262
	>5.491	18474 (15719, 21713)	0.787	5324 (4587, 6178)	0.266
MCiOP	<=0.130	18455 (15675, 21729)		4360 (3798, 5002)	
	>0.130	17916 (15466, 20755)	0.692	3901 (3391, 4488)	0.740
MiDP	<=0.720	18035 (15747, 20656)		4265 (3752, 4848)	
	>0.720	20920 (16969, 25792)	0.094	2795 (2527, 3044)	0.153
∑MBP(i+n)	<=2.860	17285 (14597, 20467)		3357 (2928, 3844)	
	>2.860 & <=5.986	19699 (16664, 23287)	0.117	4792 (4071, 5640)	0.452
	>5.986	17587 (15023, 20590)	0.832	4217 (3701, 4800)	0.458
∑DEHP	<=7.700	19726 (16650, 23370)		4467 (3870, 5152)	
	>7.700 &	17926 (15363, 20916)	0.243	4570 (3964, 5269)	0.649
	>11.320	17089 (14423, 20248)	0.087	3134 (2712, 3619)	0.522
∑DiNP	<=3.700	17889 (15136, 21142)		4068 (3478, 4759)	
	>3.700 & <=8.292	18503 (15690, 21821)	0.692	2992 (2639, 3386)	0.340
	>8.292	18125 (15388, 21348)	0.878	4848 (4163, 5645)	0.519
∑DEHPDiNP	<=9.529	18597 (15758, 21948)		4114 (3544, 4773)	
	>9.529 &	18312 (15465, 21682)	0.857	4178 (3606, 4839)	0.917
	>12.850	17462 (14858, 20523)	0.445	3860 (3344, 4454)	0.998
∑low MWphth	<=5.550	17117 (14323, 20457)		3268 (2815, 3791)	
	>5.550 &	19381 (16478, 22797)	0.137	5488 (4730, 6366)	0.134
	>12.300	17659 (15106, 20645)	0.709	3373 (2970, 3826)	0.999
∑highMWphth	<=9.770	18792 (15962, 22123)		4621 (3994, 5344)	
	>9.770 &	18290 (15411, 21706)	0.754	3810 (3281, 4422)	0.576
	>13.420	17283 (14681, 20345)	0.306	3758 (3247, 4347)	0.641
∑allphth	<=19.780	18107 (15100, 21711)		3667 (3150, 4265)	
	>19.780 &	18579 (15727, 21948)	0.766	4519 (3842, 5315)	0.530
	>30.900	17861 (15310, 20836)	0.872	3918 (3454, 4440)	0.799

Models were adjusted for age at measurement, height at measurement, maternal education and family income at birth, and length of breastfeeding. * Indicates a significant overall effect of the maternal serum phthalate levels on the outcome.

Table S20. Adjusted Associations Between Categorized Phthalate Metabolite Levels and Lean Mass (g)

		27 year followup		Change from 20-27	
		Marginal Mean (95%CI)	p	Marginal Mean (95%CI)	p
MEP	<=1.750	58668 (56361, 60974)		2834 (2832, 2836)	
	>1.750 & <=5.714	59633 (57464, 61802)	0.374	3078 (3038, 3117)	0.757
	>5.714	59391 (57347, 61435)	0.519	1344 (1286, 1403)	0.065
MiBP	<=0.792	59352 (57116, 61589)		2538 (2501, 2575)	
	>0.792 & <=1.900	59428 (57268, 61587)	0.946	2130 (2108, 2151)	0.632
	>1.900	59597 (57498, 61696)	0.821	2591 (2541, 2642)	0.947
MnBP	<=1.999	59496 (57289, 61704)		2681 (2636, 2725)	
	>1.999 & <=4.099	60869 (58698, 63040)	0.207	2745 (2680, 2811)	0.939
	>4.099	58489 (56409, 60569)	0.357	2114 (2074, 2154)	0.480
MHBP	<=0.220	59629 (57788, 61470)		2706 (2679, 2734)	
	>0.220	59254 (56902, 61606)	0.714	1811 (1731, 1890)	0.243
MBzP	<=0.260	59717 (57877, 61557)		2467 (2435, 2500)	
	>0.260	58945 (56664, 61226)	0.428	2537 (2502, 2572)	0.927
MEHP	<=2.960	59838 (57742, 61934)		1075 (1036, 1114)	*
	>2.960 & <=5.250	59439 (57299, 61578)	0.714	2731 (2650, 2812)	0.040
	>5.250	58844 (56648, 61039)	0.348	3462 (3423, 3501)	0.002
MECPP	<=0.785	57982 (55812, 60152)	*	2257 (2217, 2298)	
	>0.785 & <=1.241	59450 (57236, 61663)	0.197	2359 (2258, 2460)	0.907
	>1.241	60832 (58778, 62887)	0.007	2752 (2718, 2786)	0.535
MCMHP	<=1.153	58873 (56620, 61126)	*	2258 (2225, 2290)	
	>1.153 & <=1.810	58424 (56368, 60480)	0.685	1993 (1951, 2035)	0.753
	>1.810	61031 (58931, 63131)	0.043	2966 (2961, 2970)	0.383
MCPP	<=0.190	59710 (57732, 61689)		2602 (2566, 2638)	
	>0.190	59339 (57350, 61329)	0.679	2341 (2301, 2381)	0.697
MINP	<=2.363	58489 (56298, 60680)		1439 (1336, 1543)	
	>2.363 & <=5.491	59572 (57395, 61748)	0.331	3066 (3050, 3083)	0.067
	>5.491	60187 (58068, 62306)	0.126	2561 (2515, 2606)	0.192
MCiOP	<=0.130	58065 (55935, 60194)		2103 (2064, 2143)	
	>0.130	60419 (58515, 62324)	0.016	2617 (2582, 2651)	0.481
MiDP	<=0.720	59384 (57600, 61168)		2393 (2365, 2420)	
	>0.720	61180 (58416, 63944)	0.129	2803 (2821, 2784)	0.648
∑MBP(i+n)	<=2.860	59745 (57523, 61966)		2755 (2725, 2785)	
	>2.860 & <=5.986	60734 (58554, 62914)	0.368	2903 (2808, 2997)	0.862
	>5.986	58397 (56314, 60480)	0.215	1963 (1935, 1991)	0.313
∑DEHP	<=7.700	58845 (56611, 61080)		1757 (1718, 1796)	*
	>7.700 & <=11.320	59605 (57569, 61642)	0.483	1924 (1864, 1984)	0.838
	>11.320	59754 (57519, 61989)	0.412	3677 (3641, 3713)	0.022
∑DiNP	<=3.700	58359 (56184, 60534)		1319 (1237, 1400)	
	>3.700 & <=8.292	59874 (57709, 62039)	0.173	3299 (3272, 3327)	0.022
	>8.292	60184 (58037, 62331)	0.102	2491 (2442, 2539)	0.161
∑DEHPDiNP	<=9.529	58458 (56298, 60618)		1279 (1218, 1339)	*
	>9.529 & <=12.850	59744 (57533, 61956)	0.255	2510 (2462, 2558)	0.152
	>12.850	60201 (58081, 62322)	0.106	3257 (3220, 3293)	0.013
∑low MWphth	<=5.550	59454 (57113, 61796)		3540 (3494, 3587)	
	>5.550 & <=12.300	59439 (57311, 61566)	0.989	1743 (1690, 1796)	0.027
	>12.300	59399 (57344, 61453)	0.960	2211 (2170, 2253)	0.099
∑highMWphth	<=9.770	58363 (56233, 60492)		1271 (1217, 1325)	
	>9.770 & <=13.420	60237 (57998, 62475)	0.099	2656 (2598, 2714)	0.112
	>13.420	59964 (57828, 62101)	0.136	3146 (3111, 3181)	0.018
∑allphth	<=19.780	59139 (56776, 61501)		3372 (3338, 3407)	
	>19.780 & <=30.900	59692 (57524, 61860)	0.624	1794 (1715, 1872)	0.059
	>30.900	59502 (57487, 61516)	0.746	2552 (2529, 2575)	0.327

Models were adjusted for age at measurement, height at measurement, maternal education and family income at birth, and length of breastfeeding. * Indicates a significant overall effect of the maternal serum phthalate levels on the outcome.

Table S21. Adjusted Associations Between Categorized Phthalate Metabolite Levels and Non-Alcoholic Fatty Liver Disease (NAFLD)

		Non-Alcoholic Fatty Liver Disease	
		OR (95% CI)	p
MEP	<=1.750		
	>1.750 &	1.23 (0.24, 6.39)	0.803
	>5.714	2.27 (0.50, 10.31)	0.288
MiBP	<=0.792		
	>0.792 &	0.61 (0.10, 3.73)	0.593
	>1.900	1.58 (0.34, 7.28)	0.561
MnBP	<=1.999		
	>1.999 &	6.66 (1.11, 40.11)	0.038
	>4.099	4.08 (0.60, 27.93)	0.151
MHBP	<=0.220		
	>0.220	3.03 (0.77, 11.88)	0.112
MBzP	<=0.260		
	>0.260	2.00 (0.45, 8.82)	0.361
MEHP	<=2.960		
	>2.960 &	2.34 (0.46, 11.90)	0.306
	>5.250	1.83 (0.35, 9.68)	0.476
MECPP	<=0.785		
	>0.785 &	0.53 (0.09, 2.97)	0.469
	>1.241	0.84 (0.20, 3.52)	0.812
MCMHP	<=1.153		
	>1.153 &	1.10 (0.22, 5.65)	0.907
	>1.810	0.82 (0.19, 3.61)	0.790
MCPP	<=0.190		
	>0.190	1.77 (0.46, 6.86)	0.410
MINP	<=2.363		
	>2.363 &	1.22 (0.27, 5.59)	0.801
	>5.491	0.86 (0.17, 4.27)	0.853
MCiOP	<=0.130		
	>0.130	1.11 (0.28, 4.47)	0.881
MiDP	<=0.720		
	>0.720	1.45 (0.30, 7.08)	0.643
∑MBP(i+n)	<=2.860		
	>2.860 &	1.61 (0.33, 7.90)	0.558
	>5.986	2.52 (0.48, 13.18)	0.275
∑DEHP	<=7.700		
	>7.700 &	0.65 (0.11, 3.73)	0.626
	>11.320	2.43 (0.50, 11.87)	0.271
∑DiNP	<=3.700		
	>3.700 &	3.58 (0.64, 20.02)	0.147
	>8.292	1.64 (0.26, 10.36)	0.596
∑DEHPDiNP	<=9.529		
	>9.529 &	2.28 (0.34, 15.16)	0.394
	>12.850	3.10 (0.58, 16.58)	0.186
∑low MWphth	<=5.550		
	>5.550 &	2.38 (0.42, 13.50)	0.327
	>12.300	3.54 (0.68, 18.35)	0.132
∑highMWphth	<=9.770		
	>9.770 &	2.56 (0.38, 17.28)	0.336
	>13.420	3.09 (0.58, 16.62)	0.188
∑allphth	<=19.780		
	>19.780 &	0.51 (0.09, 2.92)	0.450
	>30.900	3.07 (0.60, 15.76)	0.178

Models were adjusted for age at measurement, BMI z-score at measurement, maternal education and family income at birth, and length of breastfeeding.

Table S22. Age Adjusted Associations Between Categorized Phthalate Metabolite Levels and Systolic Blood Pressure (mm/Hg)

		3 year followup		5 year followup		8 year followup		10 year followup		14 year followup		17 year followup		20 year followup		22 year followup	
		Marginal Mean (95%CI)	p	Marginal Mean (95%CI)	p	Marginal Mean (95%CI)	p	Marginal Mean (95%CI)	p	Marginal Mean (95%CI)	p	Marginal Mean (95%CI)	p	Marginal Mean (95%CI)	p	Marginal Mean (95%CI)	p
MEP	<=1.750	100.07 (98.56, 101.58)		101.97 (100.76, 103.18)		104.37 (103.18, 105.56)		107.90 (106.70, 109.11)		113.59 (112.22, 114.96)		118.40 (116.81, 119.98)		122.79 (120.99, 124.59)		125.26 (123.10, 127.41)	
	>1.750 & <=5.714	101.11 (99.62, 102.60)	0.276	102.58 (101.38, 103.78)	0.470	104.65 (103.47, 105.84)	0.724	107.81 (106.61, 109.00)	0.905	112.95 (111.62, 114.28)	0.488	117.31 (115.78, 118.84)	0.307	121.24 (119.51, 122.96)	0.215	123.37 (121.32, 125.43)	0.179
	>5.714	101.69 (100.15, 103.23)	0.095	103.23 (102.01, 104.45)	0.143	105.35 (104.16, 106.54)	0.229	108.56 (107.37, 109.76)	0.423	113.80 (112.44, 115.15)	0.823	118.22 (116.66, 119.79)	0.874	122.23 (120.45, 124.01)	0.659	124.42 (122.30, 126.54)	0.557
MiBP	<=0.792	100.92 (99.42, 102.42)		102.61 (101.41, 103.81)		104.85 (103.66, 106.03)		108.19 (106.99, 109.39)		113.58 (112.22, 114.95)		118.14 (116.56, 119.72)		122.26 (120.45, 124.06)		124.52 (122.36, 126.67)	
	>0.792 & <=1.900	101.53 (100.02, 103.04)	0.526	103.03 (101.82, 104.24)	0.628	105.11 (103.92, 106.31)	0.746	108.28 (107.07, 109.49)	0.916	113.42 (112.05, 114.80)	0.864	117.77 (116.17, 119.37)	0.737	121.68 (119.84, 123.51)	0.653	123.79 (121.59, 125.99)	0.613
	>1.900	100.35 (98.81, 101.88)	0.553	102.11 (100.90, 103.33)	0.561	104.41 (103.23, 105.59)	0.588	107.81 (106.63, 109.00)	0.647	113.30 (111.97, 114.63)	0.759	117.93 (116.41, 119.46)	0.848	122.13 (120.43, 123.84)	0.922	124.45 (122.44, 126.47)	0.963
MnBP	<=1.999	101.58 (100.08, 103.07)		103.20 (101.99, 104.41)		105.38 (104.19, 106.57)		108.66 (107.45, 109.87)		113.97 (112.60, 115.35)		118.46 (116.86, 120.06)		122.52 (120.68, 124.36)		124.75 (122.54, 126.95)	
	>1.999 & <=4.099	101.16 (99.64, 102.68)	0.663	102.82 (101.62, 104.02)	0.655	105.03 (103.86, 106.20)	0.665	108.34 (107.16, 109.53)	0.699	113.71 (112.37, 115.04)	0.773	118.24 (116.69, 119.78)	0.834	122.34 (120.59, 124.08)	0.885	124.59 (122.51, 126.68)	0.914
	>4.099	100.06 (98.54, 101.58)	0.117	101.73 (100.52, 102.95)	0.087	103.95 (102.77, 105.14)	0.080	107.28 (106.09, 108.46)	0.092	112.65 (111.31, 113.99)	0.154	117.19 (115.65, 118.74)	0.240	121.31 (119.57, 123.04)	0.340	123.57 (121.50, 125.63)	0.409
MHBP	<=0.220	101.13 (99.98, 102.29)		102.79 (101.95, 103.62)		105.00 (104.16, 105.85)		108.32 (107.47, 109.17)		113.69 (112.73, 114.66)		118.24 (117.12, 119.35)		122.36 (121.14, 123.57)		124.63 (123.07, 126.18)	
	>0.220	100.46 (98.83, 102.09)	0.450	102.04 (100.70, 103.38)	0.343	104.19 (102.88, 105.50)	0.282	107.44 (106.12, 108.77)	0.246	112.72 (111.22, 114.22)	0.252	117.18 (115.44, 118.91)	0.284	121.21 (119.24, 123.19)	0.326	123.42 (121.10, 125.74)	0.355
MBzP	<=0.260	101.21 (100.03, 102.38)		102.82 (101.95, 103.70)		105.00 (104.12, 105.89)		108.28 (107.38, 109.17)		113.59 (112.58, 114.60)		118.08 (116.92, 119.23)		122.14 (120.87, 123.41)		124.37 (122.76, 125.97)	
	>0.260	100.42 (98.90, 101.94)	0.347	102.12 (100.92, 103.33)	0.347	104.37 (103.20, 105.54)	0.372	107.73 (106.55, 108.91)	0.440	113.16 (111.81, 114.51)	0.588	117.74 (116.17, 119.32)	0.717	121.90 (120.11, 123.69)	0.828	124.20 (122.08, 126.32)	0.891
MEHP	<=2.960	101.79 (100.27, 103.30)		103.30 (102.09, 104.52)		105.41 (104.21, 106.60)		108.59 (107.39, 109.80)		113.79 (112.43, 115.15)		118.18 (116.61, 119.75)		122.16 (120.39, 123.93)		124.33 (122.23, 126.42)	
	>2.960 & <=5.250	99.76 (98.27, 101.25)	0.034	101.56 (100.37, 102.74)	0.040	103.87 (102.71, 105.04)	0.059	107.31 (106.13, 108.50)	0.117	112.86 (111.51, 114.21)	0.313	117.55 (115.99, 119.11)	0.554	121.83 (120.06, 123.59)	0.793	124.21 (122.11, 126.32)	0.935
	>5.250	101.36 (99.84, 102.87)	0.655	102.95 (101.75, 104.15)	0.679	105.11 (103.93, 106.29)	0.717	108.37 (107.18, 109.56)	0.784	113.66 (112.31, 115.01)	0.890	118.14 (116.57, 119.70)	0.966	122.19 (120.40, 123.98)	0.976	124.42 (122.27, 126.58)	0.945
MECPP	<=0.785	100.53 (98.98, 102.08)		102.21 (100.99, 103.43)		104.43 (103.24, 105.62)		107.74 (106.54, 108.95)		113.11 (111.75, 114.47)		117.64 (116.06, 119.23)		121.76 (119.95, 123.57)		124.03 (121.86, 126.21)	
	>0.785 & <=1.241	101.32 (99.81, 102.84)	0.415	102.79 (101.58, 104.00)	0.502	104.84 (103.65, 106.03)	0.613	107.97 (106.76, 109.17)	0.789	113.06 (111.68, 114.44)	0.957	117.37 (115.76, 118.97)	0.800	121.25 (119.42, 123.08)	0.693	123.36 (121.19, 125.52)	0.642
	>1.241	100.97 (99.48, 102.45)	0.651	102.77 (101.57, 103.97)	0.512	105.09 (103.90, 106.27)	0.419	108.52 (107.33, 109.70)	0.345	114.04 (112.72, 115.36)	0.309	118.71 (117.21, 120.20)	0.315	122.96 (121.28, 124.63)	0.335	125.33 (123.32, 127.33)	0.352
MCMHP	<=1.153	101.15 (99.61, 102.69)		102.82 (101.60, 104.05)		105.04 (103.85, 106.24)		108.37 (107.16, 109.57)		113.74 (112.38, 115.11)		118.29 (116.71, 119.87)		122.41 (120.62, 124.21)		124.68 (122.54, 126.83)	
	>1.153 & <=1.810	100.55 (99.05, 102.04)	0.536	102.03 (100.83, 103.24)	0.360	104.11 (102.92, 105.30)	0.255	107.27 (106.06, 108.47)	0.183	112.41 (111.03, 113.78)	0.152	116.76 (115.16, 118.35)	0.159	120.68 (118.85, 122.5)	0.177	122.81 (120.64, 124.97)	0.192
	>1.810	101.14 (99.63, 102.65)	0.991	102.90 (101.70, 104.10)	0.930	105.19 (104.01, 106.36)	0.861	108.59 (107.41, 109.77)	0.787	114.07 (112.75, 115.39)	0.718	118.71 (117.2, 120.22)	0.690	122.93 (121.22, 124.63)	0.679	125.27 (123.23, 127.3)	0.676

MCPP	<=0.190	101.06 (99.81, 102.30)		102.68 (101.74, 103.61)		104.86 (103.93, 105.80)		108.15 (107.20, 109.09)		113.47 (112.41, 114.54)		117.98 (116.75, 119.21)		122.05 (120.69, 123.42)		124.29 (122.59, 125.99)	
	>0.190	100.80 (99.43, 102.18)	0.749	102.47 (101.39, 103.54)	0.764	104.68 (103.62, 105.75)	0.788	108.00 (106.93, 109.08)	0.833	113.38 (112.16, 114.6)	0.906	117.93 (116.52, 119.34)	0.959	122.05 (120.47, 123.63)	1.000	124.32 (122.41, 126.23)	0.978
MINP	<=2.363	100.36 (98.80, 101.93)		102.18 (100.95, 103.41)		104.51 (103.32, 105.70)		107.96 (106.77, 109.15)		113.51 (112.15, 114.87)		118.20 (116.62, 119.77)		122.45 (120.68, 124.23)		124.82 (122.72, 126.91)	
	>2.363 & <=5.491	101.20 (99.70, 102.70)	0.391	102.64 (101.43, 103.85)	0.595	104.68 (103.48, 105.88)	0.836	107.79 (106.58, 109.00)	0.839	112.87 (111.5, 114.24)	0.488	117.16 (115.58, 118.74)	0.334	121.01 (119.21, 122.80)	0.254	123.08 (120.94, 125.22)	0.221
	>5.491	101.18 (99.70, 102.67)	0.400	102.89 (101.70, 104.09)	0.408	105.14 (103.96, 106.32)	0.438	108.50 (107.31, 109.68)	0.511	113.92 (112.59, 115.24)	0.658	118.49 (116.97, 120.01)	0.782	122.63 (120.90, 124.36)	0.885	124.92 (122.83, 127.00)	0.944
MCiOP	<=0.130	100.67 (99.13, 102.21)		102.07 (100.86, 103.28)		104.09 (102.91, 105.27)		107.18 (105.99, 108.38)		112.24 (110.89, 113.59)		116.51 (114.95, 118.08)		120.34 (118.55, 122.13)		122.40 (120.25, 124.55)	
	>0.130	101.10 (99.93, 102.27)	0.606	102.85 (101.98, 103.72)	0.296	105.13 (104.26, 106.01)	0.140	108.54 (107.65, 109.42)	0.057	114.03 (113.02, 115.03)	**0.025**	118.66 (117.51, 119.81)	**0.021**	122.86 (121.61, 124.11)	**0.021**	125.18 (123.60, 126.75)	**0.023**
MiDP	<=0.720	100.68 (99.58, 101.78)		102.40 (101.61, 103.18)		104.65 (103.85, 105.45)		108.02 (107.20, 108.83)		113.46 (112.53, 114.38)		118.06 (117.00, 119.11)		122.24 (121.09, 123.38)		124.56 (123.08, 126.04)	
	>0.720	102.31 (100.28, 104.33)	0.125	103.56 (101.86, 105.25)	0.216	105.46 (103.82, 107.09)	0.366	108.41 (106.76, 110.06)	0.662	113.27 (111.39, 115.15)	0.855	117.38 (115.20, 119.57)	0.571	121.07 (118.54, 123.6)	0.403	123.03 (120.12, 125.95)	0.331
∑MBP(i+n)	<=2.860	101.38 (99.88, 102.89)		102.92 (101.71, 104.12)		105.04 (103.85, 106.23)		108.25 (107.05, 109.46)		113.48 (112.11, 114.84)		117.89 (116.30, 119.47)		121.86 (120.04, 123.68)		124.02 (121.83, 126.20)	
	>2.860 & <=5.986	101.54 (100.02, 103.06)	0.871	103.22 (102.02, 104.43)	0.721	105.46 (104.28, 106.64)	0.606	108.81 (107.62, 110.00)	0.500	114.22 (112.87, 115.57)	0.425	118.78 (117.22, 120.35)	0.407	122.92 (121.14, 124.69)	0.408	125.19 (123.07, 127.30)	0.414
	>5.986	99.92 (98.41, 101.43)	0.128	101.61 (100.41, 102.82)	0.126	103.86 (102.68, 105.03)	0.145	107.21 (106.03, 108.39)	0.203	112.64 (111.31, 113.97)	0.361	117.22 (115.68, 118.75)	0.530	121.36 (119.64, 123.09)	0.692	123.64 (121.59, 125.69)	0.789
∑DEHP	<=7.700	100.76 (99.23, 102.30)		102.40 (101.17, 103.63)		104.59 (103.38, 105.80)		107.88 (106.66, 109.09)		113.21 (111.83, 114.59)		117.72 (116.12, 119.32)		121.81 (120.00, 123.63)		124.08 (121.93, 126.22)	
	>7.700 & <=11.320	101.07 (99.58, 102.56)	0.749	102.56 (101.37, 103.75)	0.850	104.64 (103.47, 105.81)	0.951	107.80 (106.61, 108.98)	0.921	112.94 (111.59, 114.29)	0.773	117.29 (115.73, 118.85)	0.695	121.23 (119.47, 122.99)	0.647	123.38 (121.29, 125.48)	0.625
	>11.320	100.98 (99.46, 102.50)	0.827	102.79 (101.58, 104.00)	0.655	105.11 (103.93, 106.30)	0.526	108.55 (107.37, 109.74)	0.412	114.1 (112.77, 115.43)	0.334	118.79 (117.26, 120.32)	0.316	123.08 (121.33, 124.82)	0.318	125.47 (123.37, 127.58)	0.324
∑DiNP	<=3.700	100.41 (98.85, 101.98)		102.08 (100.85, 103.31)		104.29 (103.10, 105.48)		107.60 (106.40, 108.79)		112.95 (111.59, 114.32)		117.48 (115.89, 119.07)		121.58 (119.79, 123.37)		123.84 (121.74, 125.94)	
	>3.700 & <=8.292	101.25 (99.75, 102.74)	0.393	102.77 (101.57, 103.98)	0.420	104.88 (103.69, 106.07)	0.472	108.06 (106.86, 109.27)	0.572	113.25 (111.89, 114.61)	0.752	117.63 (116.06, 119.19)	0.891	121.58 (119.79, 123.37)	1.000	123.73 (121.59, 125.88)	0.941
	>8.292	101.12 (99.63, 102.60)	0.470	102.89 (101.69, 104.08)	0.345	105.18 (104.00, 106.36)	0.272	108.59 (107.40, 109.78)	0.224	114.08 (112.75, 115.41)	0.217	118.72 (117.19, 120.25)	0.242	122.94 (121.20, 124.68)	0.277	125.29 (123.19, 127.38)	0.303
∑DEHPDiNP	<=9.529	100.78 (99.25, 102.31)		102.32 (101.10, 103.54)		104.44 (103.25, 105.63)		107.63 (106.42, 108.83)		112.82 (111.44, 114.19)		117.21 (115.61, 118.81)		121.20 (119.39, 123.01)		123.39 (121.26, 125.51)	
	>9.529 & <=12.850	101.07 (99.56, 102.59)	0.761	102.68 (101.47, 103.89)	0.683	104.83 (103.64, 106.02)	0.629	108.07 (106.87, 109.28)	0.588	113.34 (111.97, 114.70)	0.578	117.79 (116.21, 119.38)	0.595	121.84 (120.03, 123.64)	0.618	124.07 (121.91, 126.23)	0.634
	>12.850	100.98 (99.49, 102.48)	0.833	102.79 (101.59, 103.99)	0.586	105.11 (103.92, 106.29)	0.412	108.53 (107.35, 109.72)	0.269	114.05 (112.73, 115.38)	0.178	118.73 (117.22, 120.24)	0.155	122.99 (121.29, 124.70)	0.151	125.39 (123.32, 127.45)	0.153
∑low MWphth	<=5.550	100.19 (98.68, 101.70)		101.91 (100.71, 103.12)		104.18 (102.99, 105.36)		107.55 (106.35, 108.76)		113.01 (111.63, 114.38)		117.61 (116.01, 119.21)		121.80 (119.97, 123.64)		124.13 (121.93, 126.32)	
	>5.550 & <=12.300	101.93 (100.42, 103.45)	0.071	103.50 (102.29, 104.71)	0.064	105.64 (104.45, 106.83)	0.074	108.87 (107.66, 110.07)	0.111	114.11 (112.76, 115.47)	0.235	118.55 (116.99, 120.11)	0.389	122.56 (120.79, 124.34)	0.554	124.76 (122.64, 126.88)	0.659
	>12.300	100.72 (99.20, 102.23)	0.582	102.36 (101.15, 103.57)	0.602	104.56 (103.38, 105.74)	0.641	107.86 (106.67, 109.04)	0.712	113.20 (111.87, 114.53)	0.834	117.72 (116.18, 119.25)	0.924	121.81 (120.09, 123.54)	0.994	124.07 (122.02, 126.12)	0.968
∑highMWphth	<=9.770	100.96 (99.43, 102.50)		102.51 (101.29, 103.73)		104.63 (103.44, 105.82)		107.82 (106.62, 109.02)		113.01 (111.63, 114.39)		117.40 (115.80, 119.00)		121.39 (119.58, 123.19)		123.58 (121.46, 125.7)	
	>9.770 & <=13.420	100.97 (99.46, 102.47)	0.996	102.51 (101.30, 103.72)	0.997	104.62 (103.43, 105.81)	0.991	107.80 (106.60, 109.01)	0.985	112.98 (111.61, 114.35)	0.978	117.37 (115.78, 118.96)	0.975	121.34 (119.53, 123.16)	0.974	123.53 (121.37, 125.69)	0.974

	>13.420	100.91<br>(99.41, 102.40)	0.955	102.77<br>(101.58, 103.97)	0.762	105.13<br>(103.95, 106.31)	0.539	108.60<br>(107.42, 109.79)	0.340	114.19<br>(112.87, 115.51)	0.199	118.91<br>(117.41, 120.41)	0.155	123.23<br>(121.54, 124.93)	0.139	125.67<br>(123.62, 127.72)	0.134
∑allphth	<=19.780	100.96<br>(99.42, 102.50)		102.52<br>(101.30, 103.74)		104.65<br>(103.46, 105.84)		107.87<br>(106.66, 109.08)		113.10<br>(111.71, 114.49)		117.51<br>(115.89, 119.14)		121.50<br>(119.63, 123.38)		123.68<br>(121.45, 125.91)	
	>19.780 &<br><=30.900	101.32<br>(99.84, 102.80)	0.712	102.97<br>(101.77, 104.17)	0.600	105.17<br>(103.98, 106.36)	0.523	108.47<br>(107.28, 109.67)	0.464	113.82<br>(112.48, 115.16)	0.439	118.33<br>(116.80, 119.87)	0.451	122.42<br>(120.69, 124.15)	0.474	124.67<br>(122.60, 126.74)	0.491
	>30.900	100.54<br>(99.01, 102.07)	0.666	102.27<br>(101.06, 103.49)	0.774	104.54<br>(103.36, 105.72)	0.889	107.91<br>(106.73, 109.1)	0.959	113.36<br>(112.02, 114.70)	0.778	117.96<br>(116.42, 119.50)	0.681	122.14<br>(120.41, 123.87)	0.620	124.45<br>(122.39, 126.52)	0.593

Models were adjusted for age at measurement.

Table S23. Age Adjusted Associations Between Categorized Phthalate Metabolite Levels and Diastolic Blood Pressure (mm/Hg)

		3 year followup		5 year followup		8 year followup		10 year followup		14 year followup		17 year followup		20 year followup		22 year followup	
		Marginal Mean (95%CI)	p	Marginal Mean (95%CI)	p	Marginal Mean (95%CI)	p	Marginal Mean (95%CI)	p	Marginal Mean (95%CI)	p	Marginal Mean (95%CI)	p	Marginal Mean (95%CI)	p	Marginal Mean (95%CI)	p
MEP	<=1.750	52.85 (51.52, 54.18)		54.61 (53.55, 55.67)		55.57 (54.58, 56.57)		56.57 (55.63, 57.51)		58.28 (57.31, 59.24)		60.55 (59.48, 61.62)		64.26 (63.08, 65.44)		68.02 (66.58, 69.46)	
	>1.750 & <=5.714	53.25 (51.93, 54.56)	0.644	54.78 (53.72, 55.83)	0.824	55.56 (54.57, 56.56)	0.987	56.35 (55.41, 57.28)	0.731	57.76 (56.81, 58.71)	0.426	59.80 (58.76, 60.83)	0.285	63.25 (62.12, 64.38)	0.217	66.84 (65.46, 68.21)	0.194
	>5.714	54.74 (53.39, 56.09)	0.030	56.03 (54.97, 57.10)	0.060	56.64 (55.64, 57.63)	0.123	57.20 (56.27, 58.14)	0.320	58.32 (57.36, 59.28)	0.944	60.10 (59.04, 61.16)	0.530	63.30 (62.14, 64.46)	0.248	66.70 (65.28, 68.12)	0.155
MiBP	<=0.792	53.70 (52.37, 55.03)		55.18 (54.12, 56.24)		55.93 (54.93, 56.92)		56.66 (55.72, 57.60)		58.00 (57.03, 58.96)		59.95 (58.89, 61.02)		63.33 (62.15, 64.51)		66.85 (65.41, 68.29)	
	>0.792 & <=1.900	53.27 (51.94, 54.61)	0.626	54.83 (53.76, 55.90)	0.643	55.63 (54.63, 56.64)	0.671	56.43 (55.49, 57.38)	0.727	57.87 (56.89, 58.84)	0.843	59.90 (58.82, 60.99)	0.945	63.36 (62.15, 64.57)	0.970	66.94 (65.46, 68.42)	0.925
	>1.900	53.80 (52.44, 55.15)	0.913	55.37 (54.30, 56.44)	0.801	56.19 (55.20, 57.18)	0.702	57.01 (56.08, 57.93)	0.584	58.46 (57.52, 59.41)	0.462	60.52 (59.50, 61.55)	0.415	64.00 (62.90, 65.10)	0.405	67.60 (66.26, 68.93)	0.409
MnBP	<=1.999	52.83 (51.51, 54.16)		54.41 (53.35, 55.47)		55.23 (54.23, 56.23)		56.05 (55.10, 570)		57.52 (56.54, 58.50)		59.59 (58.51, 60.68)		63.09 (61.87, 64.30)		66.70 (65.21, 68.18)	
	>1.999 & <=4.099	53.93 (52.59, 55.27)	0.209	55.41 (54.36, 56.47)	0.183	56.16 (55.18, 57.15)	0.173	56.90 (55.98, 57.83)	0.182	58.26 (57.31, 59.21)	0.252	60.24 (59.20, 61.28)	0.366	63.64 (62.50, 64.77)	0.508	67.18 (65.79, 68.57)	0.605
	>4.099	54.04 (52.70, 55.39)	0.167	55.57 (54.50, 56.64)	0.126	56.35 (55.36, 57.35)	0.103	57.13 (56.20, 58.06)	0.093	58.54 (57.59, 59.49)	0.116	60.56 (59.52, 61.60)	0.176	64.00 (62.87, 65.13)	0.269	67.58 (66.20, 68.95)	0.344
MHBP	<=0.220	53.47 (52.47, 54.47)		55.08 (54.35, 55.81)		55.92 (55.21, 56.62)		56.75 (56.08, 57.42)		58.23 (57.53, 58.92)		60.31 (59.54, 61.07)		63.82 (63.02, 64.61)		67.44 (66.39, 68.50)	
	>0.220	53.87 (52.42, 55.31)	0.623	55.26 (54.09, 56.44)	0.791	55.94 (54.84, 57.04)	0.970	56.59 (55.55, 57.62)	0.782	57.80 (56.74, 58.86)	0.473	59.66 (58.49, 60.83)	0.323	62.94 (61.65, 64.23)	0.249	66.41 (64.87, 67.95)	0.222
MBzP	<=0.260	53.50 (52.47, 54.53)		55.03 (54.27, 55.80)		55.82 (55.08, 56.56)		56.60 (55.9, 57.30)		58.01 (57.29, 58.73)		60.03 (59.24, 60.82)		63.47 (62.64, 64.30)		67.04 (65.95, 68.13)	
	>0.260	53.76 (52.42, 55.11)	0.727	55.31 (54.26, 56.37)	0.670	56.11 (55.13, 57.10)	0.626	56.90 (55.98, 57.83)	0.583	58.33 (57.37, 59.29)	0.564	60.37 (59.29, 61.44)	0.585	63.82 (62.64, 65.00)	0.622	67.40 (65.98, 68.83)	0.650
MEHP	<=2.960	53.76 (52.43, 55.10)		55.24 (54.17, 56.31)		55.98 (54.98, 56.98)		56.71 (55.77, 57.65)		58.05 (57.08, 59.01)		60.01 (58.95, 61.07)		63.39 (62.24, 64.54)		66.92 (65.53, 68.31)	
	>2.960 & <=5.250	52.65 (51.33, 53.96)	0.197	54.36 (53.31, 55.41)	0.245	55.29 (54.30, 56.28)	0.315	56.24 (55.31, 57.17)	0.461	57.88 (56.92, 58.84)	0.796	60.10 (59.04, 61.15)	0.900	63.74 (62.58, 64.90)	0.668	67.45 (66.05, 68.86)	0.557
	>5.250	54.40 (53.06, 55.74)	0.466	55.81 (54.75, 56.87)	0.450	56.50 (55.51, 57.50)	0.448	57.17 (56.24, 58.11)	0.468	58.43 (57.47, 59.39)	0.551	60.32 (59.27, 61.38)	0.655	63.64 (62.46, 64.81)	0.763	67.12 (65.67, 68.56)	0.828
MECPP	<=0.785	54.24 (52.88, 55.60)		55.81 (54.74, 56.88)		56.62 (55.62, 57.62)		57.42 (56.49, 58.36)		58.86 (57.90, 59.83)		60.91 (59.84, 61.99)		64.39 (63.20, 65.58)		68.00 (66.54, 69.46)	
	>0.785 & <=1.241	53.31 (51.98, 54.64)	0.292	54.77 (53.71, 55.83)	0.170	55.49 (54.49, 56.48)	0.101	56.19 (55.25, 57.13)	0.055	57.48 (56.50, 58.46)	0.034	59.41 (58.32, 60.50)	0.038	62.76 (61.56, 63.96)	0.053	66.27 (64.82, 67.72)	0.069
	>1.241	53.23 (51.92, 54.54)	0.249	54.83 (53.78, 55.89)	0.197	55.67 (54.68, 56.66)	0.166	56.50 (55.57, 57.43)	0.146	57.98 (57.04, 58.91)	0.164	60.06 (59.05, 61.07)	0.220	63.57 (62.48, 64.66)	0.307	67.19 (65.86, 68.53)	0.374
MCMHP	<=1.153	53.58 (52.22, 54.94)		55.22 (54.15, 56.30)		56.09 (55.09, 57.10)		56.96 (56.02, 57.9)		58.49 (57.52, 59.46)		60.61 (59.54, 61.68)		64.16 (62.98, 65.34)		67.82 (66.38, 69.26)	
	>1.153 & <=1.810	53.40 (52.08, 54.73)	0.844	54.86 (53.80, 55.92)	0.633	55.58 (54.58, 56.58)	0.462	56.28 (55.34, 57.22)	0.293	57.57 (56.60, 58.55)	0.160	59.50 (58.41, 60.58)	0.122	62.84 (61.64, 64.04)	0.115	66.35 (64.90, 67.80)	0.119
	>1.810	53.76 (52.42, 55.09)	0.839	55.30 (54.24, 56.35)	0.922	56.09 (55.10, 57.07)	0.994	56.86 (55.94, 57.78)	0.878	58.26 (57.32, 59.20)	0.720	60.28 (59.25, 61.30)	0.630	63.71 (62.60, 64.82)	0.579	67.29 (65.93, 68.65)	0.559
MCPP	<=0.190	53.37 (52.28, 54.45)		54.95 (54.14, 55.77)		55.78 (55.00, 56.56)		56.60 (55.86, 57.34)		58.06 (57.30, 58.83)		60.13 (59.29, 60.97)		63.62 (62.72, 64.52)		67.23 (66.08, 68.38)	

	>0.190	53.88 (52.67, 55.09)	0.478	55.37 (54.42, 56.31)	0.509	56.12 (55.22, 57.01)	0.553	56.85 (56.01, 57.69)	0.638	58.19 (57.32, 59.06)	0.814	60.15 (59.19, 61.11)	0.970	63.54 (62.50, 64.57)	0.901	67.07 (65.79, 68.35)	0.833
MINP	<=2.363	53.97 (52.60, 55.34)		55.46 (54.39, 56.54)		56.22 (55.23, 57.22)		56.97 (56.03, 57.90)		58.33 (57.36, 59.29)		60.31 (59.24, 61.38)		63.72 (62.55, 64.89)		67.27 (65.86, 68.67)	
	>2.363 & <=5.491	52.95 (51.63, 54.28)	0.248	54.52 (53.45, 55.58)	0.213	55.32 (54.32, 56.33)	0.194	56.13 (55.18, 57.07)	0.193	57.57 (56.60, 58.54)	0.245	59.62 (58.56, 60.69)	0.338	63.10 (61.93, 64.28)	0.459	66.70 (65.26, 68.14)	0.544
	>5.491	53.86 (52.55, 55.18)	0.905	55.41 (54.36, 56.46)	0.942	56.20 (55.22, 57.19)	0.980	56.99 (56.06, 57.92)	0.966	58.42 (57.47, 59.36)	0.889	60.45 (59.42, 61.48)	0.841	63.91 (62.77, 65.05)	0.812	67.50 (66.09, 68.9)	0.800
MCiOP	<=0.130	54.07 (52.71, 55.42)		55.51 (54.45, 56.58)		56.24 (55.24, 57.23)		56.94 (56.00, 57.88)		58.24 (57.28, 59.21)		60.17 (59.10, 61.24)		63.52 (62.33, 64.71)		67.02 (65.57, 68.48)	
	>0.130	53.36 (52.34, 54.38)	0.355	54.94 (54.18, 55.70)	0.384	55.76 (55.03, 56.50)	0.430	56.59 (55.89, 57.28)	0.524	58.05 (57.33, 58.78)	0.736	60.12 (59.33, 60.92)	0.934	63.61 (62.78, 64.44)	0.901	67.21 (66.14, 68.29)	0.814
MiDP	<=0.720	53.56 (52.60, 54.51)		55.13 (54.45, 55.82)		55.95 (55.28, 56.62)		56.76 (56.12, 57.40)		58.21 (57.54, 58.87)		60.26 (59.53, 60.99)		63.75 (63.00, 64.50)		67.35 (66.34, 68.37)	
	>0.720	53.75 (51.94, 55.55)	0.841	55.13 (53.64, 56.62)	0.993	55.79 (54.42, 57.16)	0.831	56.42 (55.13, 57.71)	0.629	57.62 (56.30, 58.94)	0.406	59.46 (58.00, 60.93)	0.309	62.73 (61.08, 64.38)	0.265	66.19 (64.26, 68.11)	0.253
∑MBP(i+n)	<=2.860	53.04 (51.71, 54.37)		54.53 (53.47, 55.59)		55.29 (54.29, 56.29)		56.04 (55.09, 56.98)		57.40 (56.43, 58.37)		59.38 (58.31, 60.45)		62.78 (61.58, 63.97)		66.31 (64.84, 67.78)	
	>2.860 & <=5.986	53.96 (52.61, 55.30)	0.294	55.52 (54.45, 56.58)	0.192	56.33 (55.34, 57.33)	0.131	57.14 (56.21, 58.07)	0.086	58.59 (57.63, 59.55)	0.067	60.64 (59.59, 61.70)	0.078	64.11 (62.96, 65.27)	0.108	67.70 (66.29, 69.11)	0.136
	>5.986	53.79 (52.45, 55.13)	0.390	55.33 (54.27, 56.39)	0.292	56.12 (55.13, 57.12)	0.225	56.91 (55.99, 57.84)	0.171	58.34 (57.39, 59.28)	0.145	60.37 (59.33, 61.40)	0.164	63.81 (62.69, 64.93)	0.205	67.38 (66.02, 68.74)	0.241
∑DEHP	<=7.700	53.88 (52.53, 55.23)		55.34 (54.26, 56.42)		56.07 (55.06, 57.08)		56.77 (55.82, 57.72)		58.07 (57.09, 59.05)		60.00 (58.92, 61.09)		63.36 (62.17, 64.55)		66.89 (65.45, 68.32)	
	>7.700 & <=11.320	52.89 (51.58, 54.20)	0.255	54.53 (53.49, 55.58)	0.283	55.39 (54.41, 56.38)	0.328	56.26 (55.33, 57.19)	0.424	57.78 (56.82, 58.74)	0.657	59.90 (58.85, 60.96)	0.889	63.46 (62.30, 64.61)	0.910	67.12 (65.72, 68.52)	0.803
	>11.320	54.03 (52.69, 55.37)	0.868	55.55 (54.49, 56.62)	0.781	56.33 (55.34, 57.32)	0.705	57.09 (56.16, 58.02)	0.618	58.47 (57.53, 59.42)	0.533	60.48 (59.44, 61.51)	0.508	63.91 (62.76, 65.05)	0.511	67.48 (66.06, 68.89)	0.521
∑DiNP	<=3.700	53.98 (52.61, 55.36)		55.38 (54.30, 56.46)		56.06 (55.06, 57.05)		56.70 (55.77, 57.64)		57.92 (56.95, 58.90)		59.80 (58.72, 60.88)		63.11 (61.93, 64.28)		66.60 (65.20, 68.01)	
	>3.700 & <=8.292	52.88 (51.56, 54.20)	0.209	54.58 (53.52, 55.64)	0.294	55.49 (54.49, 56.49)	0.415	56.41 (55.47, 57.36)	0.654	58.02 (57.06, 58.98)	0.882	60.22 (59.16, 61.27)	0.559	63.86 (62.69, 65.03)	0.367	67.59 (66.15, 69.03)	0.290
	>8.292	53.96 (52.64, 55.28)	0.977	55.47 (54.42, 56.52)	0.907	56.23 (55.24, 57.23)	0.797	56.98 (56.05, 57.91)	0.662	58.35 (57.40, 59.29)	0.509	60.34 (59.31, 61.38)	0.441	63.78 (62.64, 64.92)	0.414	67.36 (65.96, 68.77)	0.409
∑DEHPDiNP	<=9.529	53.71 (52.36, 55.06)		55.14 (54.07, 56.21)		55.85 (54.85, 56.84)		56.52 (55.58, 57.46)		57.78 (56.80, 58.76)		59.68 (58.60, 60.77)		63.02 (61.83, 64.20)		66.52 (65.11, 67.94)	
	>9.529 & <=12.850	53.09 (51.75, 54.43)	0.479	54.81 (53.75, 55.88)	0.665	55.74 (54.74, 56.74)	0.880	56.68 (55.74, 57.62)	0.807	58.31 (57.34, 59.28)	0.421	60.52 (59.44, 61.59)	0.249	64.17 (62.98, 65.35)	0.171	67.90 (66.45, 69.34)	0.144
	>12.850	53.96 (52.64, 55.29)	0.773	55.45 (54.39, 56.5)	0.687	56.19 (55.20, 57.18)	0.616	56.91 (55.98, 57.84)	0.540	58.24 (57.30, 59.18)	0.475	60.20 (59.18, 61.21)	0.467	63.59 (62.47, 64.70)	0.484	67.13 (65.75, 68.52)	0.503
∑low MWphth	<=5.550	53.18 (51.85, 54.51)		54.78 (53.72, 55.84)		55.61 (54.62, 56.61)		56.44 (55.50, 57.38)		57.92 (56.95, 58.89)		60.01 (58.93, 61.08)		63.52 (62.32, 64.72)		67.16 (65.69, 68.62)	
	>5.550 & <=12.300	53.26 (51.92, 54.60)	0.930	54.95 (53.89, 56.02)	0.816	55.86 (54.86, 56.87)	0.714	56.78 (55.84, 57.73)	0.595	58.39 (57.43, 59.35)	0.472	60.58 (59.53, 61.63)	0.426	64.20 (63.05, 65.36)	0.414	67.91 (66.51, 69.32)	0.417
	>12.300	54.33 (52.99, 55.67)	0.186	55.67 (54.61, 56.73)	0.239	56.30 (55.31, 57.29)	0.316	56.89 (55.97, 57.82)	0.478	58.04 (57.10, 58.99)	0.848	59.85 (58.82, 60.89)	0.830	63.09 (61.97, 64.21)	0.595	66.52 (65.16, 67.88)	0.488
∑highMWphth	<=9.770	53.51 (52.16, 54.87)		55.02 (53.94, 56.09)		55.77 (54.77, 56.77)		56.51 (55.57, 57.45)		57.86 (56.88, 58.84)		59.83 (58.74, 60.92)		63.23 (62.04, 64.42)		66.79 (65.36, 68.21)	
	>9.770 & <=13.420	53.29 (51.95, 54.63)	0.797	54.92 (53.85, 55.99)	0.899	55.77 (54.77, 56.77)	0.997	56.63 (55.69, 57.57)	0.853	58.14 (57.17, 59.11)	0.666	60.25 (59.17, 61.33)	0.563	63.79 (62.60, 64.99)	0.507	67.44 (65.99, 68.90)	0.485
	>13.420	53.95 (52.63, 55.28)	0.616	55.46 (54.41, 56.52)	0.555	56.23 (55.23, 57.22)	0.509	56.97 (56.04, 57.90)	0.469	58.33 (57.40, 59.27)	0.459	60.31 (59.3, 61.33)	0.491	63.73 (62.62, 64.83)	0.543	67.29 (65.91, 68.66)	0.581

∑allphth	<=19.780	53.66 (52.31, 55.01)		55.17 (54.10, 56.24)		55.94 (54.94, 56.94)		56.70 (55.76, 57.65)		58.09 (57.11, 59.08)		60.10 (59.00, 61.21)		63.54 (62.31, 64.77)		67.11 (65.61, 68.60)	
	>19.780 & <=30.900	52.92 (51.61, 54.23)	0.399	54.63 (53.57, 55.68)	0.474	55.55 (54.55, 56.55)	0.569	56.49 (55.55, 57.43)	0.734	58.12 (57.17, 59.07)	0.969	60.33 (59.30, 61.37)	0.750	63.98 (62.85, 65.10)	0.599	67.69 (66.32, 69.07)	0.529
	>30.900	54.27 (52.92, 55.61)	0.487	55.63 (54.56, 56.69)	0.547	56.28 (55.29, 57.28)	0.621	56.91 (55.98, 57.84)	0.749	58.11 (57.16, 59.06)	0.982	59.96 (58.92, 61.00)	0.841	63.23 (62.10, 64.36)	0.712	66.68 (65.31, 68.06)	0.651

Models were adjusted for age at measurement.

Table S24. Age Adjusted Associations Between Categorized Phthalate Metabolite Levels and Heartrate (Beats per Minute- BPM)

		5 year followup		8 year followup		10 year followup		14 year followup		17 year followup		20 year followup		22 year followup	
		Marginal Mean (95%CI)	p	Marginal Mean (95%CI)	p	Marginal Mean (95%CI)	p	Marginal Mean (95%CI)	p	Marginal Mean (95%CI)	p	Marginal Mean (95%CI)	p	Marginal Mean (95%CI)	p
MEP	<=1.750	88.90 (86.97, 90.84)		85.28 (83.75, 86.80)		80.88 (79.38, 82.38)		75.52 (74.01, 77.03)		72.81 (71.07, 74.54)		72.77 (70.79, 74.74)		74.98 (72.60, 77.37)	
	>1.750 & <=5.714	87.78 (85.82, 89.73)	0.353	84.06 (82.55, 85.57)	0.259	79.56 (78.09, 81.03)	0.179	74.05 (72.58, 75.52)	0.141	71.22 (69.55, 72.89)	0.161	71.05 (69.17, 72.93)	0.207	73.18 (70.89, 75.46)	0.244
	>5.714	86.61 (84.58, 88.64)	0.062	82.97 (81.43, 84.50)	0.034	78.55 (77.08, 80.02)	0.019	73.15 (71.66, 74.64)	0.019	70.41 (68.70, 72.13)	0.037	70.35 (68.40, 72.29)	0.080	72.54 (70.19, 74.9)	0.121
MiBP	<=0.792	89.53 (87.71, 91.35)	*	85.77 (84.30, 87.24)	*	81.16 (79.67, 82.64)	*	75.42 (73.92, 76.92)		72.39 (70.67, 74.10)		72.05 (70.09, 74.01)		74.09 (71.70, 76.48)	
	>0.792 & <=1.900	85.42 (83.41, 87.43)	0.001	82.12 (80.58, 83.66)	0.001	78.05 (76.56, 79.54)	0.002	73.07 (71.55, 74.58)	0.020	70.67 (68.92, 72.42)	0.140	70.98 (68.97, 72.99)	0.446	73.49 (71.03, 75.94)	0.705
	>1.900	87.89 (85.85, 89.93)	0.171	84.23 (82.71, 85.76)	0.149	79.74 (78.30, 81.18)	0.146	74.18 (72.73, 75.63)	0.208	71.29 (69.62, 72.95)	0.327	71.09 (69.25, 72.94)	0.477	73.24 (71.04, 75.45)	0.578
MnBP	<=1.999	88.40 (86.52, 90.28)		84.69 (83.18, 86.21)		80.21 (78.68, 81.73)		74.72 (73.18, 76.25)		71.90 (70.15, 73.65)		71.74 (69.72, 73.76)		73.87 (71.41, 76.33)	
	>1.999 & <=4.099	86.95 (84.96, 88.93)	0.230	83.30 (81.77, 84.82)	0.196	78.88 (77.40, 80.35)	0.179	73.49 (72.01, 74.97)	0.223	70.75 (69.06, 72.44)	0.319	70.68 (68.78, 72.57)	0.440	72.86 (70.56, 75.16)	0.523
	>4.099	87.94 (85.87, 90.02)	0.710	84.30 (82.75, 85.85)	0.718	79.88 (78.42, 81.34)	0.746	74.50 (73.03, 75.97)	0.828	71.77 (70.08, 73.46)	0.908	71.70 (69.81, 73.59)	0.975	73.89 (71.61, 76.16)	0.990
MHBP	<=0.220	87.39 (85.84, 88.94)		83.77 (82.72, 84.83)		79.40 (78.32, 80.48)		74.08 (72.99, 75.16)		71.40 (70.15, 72.64)		71.39 (70.05, 72.73)		73.62 (71.89, 75.35)	
	>0.220	88.77 (86.70, 90.84)	0.212	84.94 (83.27, 86.61)	0.238	80.31 (78.68, 81.95)	0.315	74.63 (72.97, 76.28)	0.550	71.65 (69.76, 73.54)	0.810	71.33 (69.18, 73.49)	0.965	73.35 (70.79, 75.91)	0.849
MBzP	<=0.260	87.93 (86.41, 89.45)		84.24 (83.15, 85.33)		79.78 (78.63, 80.93)		74.34 (73.21, 75.47)		71.57 (70.30, 72.85)		71.47 (70.08, 72.86)		73.63 (71.84, 75.43)	
	>0.260	87.48 (85.39, 89.57)	0.680	83.82 (82.26, 85.37)	0.658	79.39 (77.92, 80.85)	0.646	73.98 (72.49, 75.48)	0.680	71.25 (69.51, 72.99)	0.741	71.18 (69.20, 73.15)	0.805	73.36 (71.01, 75.71)	0.842
MEHP	<=2.960	86.75 (84.68, 88.82)		83.17 (81.59, 84.75)		78.83 (77.33, 80.33)		73.55 (72.05, 75.05)		70.91 (69.18, 72.63)		70.92 (68.99, 72.85)		73.16 (70.86, 75.46)	
	>2.960 & <=5.250	88.88 (86.95, 90.81)	0.084	85.16 (83.66, 86.66)	0.069	80.66 (79.18, 82.13)	0.066	75.15 (73.66, 76.64)	0.110	72.31 (70.61, 74.02)	0.215	72.13 (70.20, 74.06)	0.372	74.23 (71.90, 76.56)	0.489
	>5.250	87.57 (85.65, 89.49)	0.507	83.88 (82.38, 85.38)	0.516	79.42 (77.94, 80.9)	0.557	73.96 (72.47, 75.45)	0.685	71.16 (69.46, 72.87)	0.820	71.03 (69.07, 72.98)	0.938	73.16 (70.75, 75.57)	0.999
MECPP	<=0.785	88.78 (86.82, 90.75)		85.17 (83.64, 86.71)		80.82 (79.32, 82.31)		75.53 (74.02, 77.03)		72.89 (71.16, 74.62)		72.93 (70.94, 74.91)		75.19 (72.77, 77.62)	
	>0.785 & <=1.241	86.67 (84.67, 88.67)	0.086	83.15 (81.62, 84.68)	0.064	78.90 (77.41, 80.38)	0.054	73.76 (72.24, 75.27)	0.082	71.24 (69.48, 73.01)	0.158	71.41 (69.40, 73.42)	0.280	73.76 (71.36, 76.17)	0.374
	>1.241	87.88 (85.92, 89.85)	0.461	83.98 (82.46, 85.49)	0.269	79.27 (77.80, 80.74)	0.116	73.49 (72.04, 74.94)	0.040	70.45 (68.82, 72.08)	0.030	70.07 (68.25, 71.88)	0.033	72.04 (69.83, 74.24)	0.039
MCMHP	<=1.153	88.19 (86.24, 90.14)		84.65 (83.11, 86.18)		80.37 (78.86, 81.87)		75.18 (73.67, 76.69)		72.63 (70.91, 74.35)		72.75 (70.80, 74.71)		75.07 (72.71, 77.44)	

	>1.153 & <=1.810	86.67 (84.65, 88.69)	0.218	83.18 (81.63, 84.72)	0.180	78.96 (77.47, 80.45)	0.160	73.86 (72.35, 75.38)	0.195	71.39 (69.64, 73.13)	0.283	71.58 (69.59, 73.57)	0.401	73.96 (71.56, 76.36)	0.482
	>1.810	88.44 (86.49, 90.38)	0.841	84.44 (82.94, 85.94)	0.847	79.63 (78.17, 81.09)	0.453	73.70 (72.25, 75.15)	0.136	70.53 (68.88, 72.18)	0.061	70.02 (68.18, 71.86)	**0.041**	71.89 (69.65, 74.12)	**0.036**
MCPP	<=0.190	88.14 (86.46, 89.82)		84.53 (83.34, 85.71)		80.18 (79.00, 81.36)		74.92 (73.74, 76.10)		72.31 (70.95, 73.67)		72.37 (70.88, 73.86)		74.63 (72.76, 76.51)	
	>0.190	87.36 (85.60, 89.11)	0.438	83.51 (82.16, 84.85)	0.255	78.89 (77.54, 80.24)	0.112	73.24 (71.89, 74.59)	**0.041**	70.31 (68.77, 71.85)	**0.032**	70.03 (68.32, 71.74)	**0.037**	72.06 (69.95, 74.16)	**0.043**
MINP	<=2.363	88.00 (85.80, 90.19)		84.25 (82.63, 85.86)		79.75 (78.26, 81.23)		74.29 (72.79, 75.80)		71.52 (69.78, 73.27)		71.41 (69.45, 73.37)		73.56 (71.23, 75.88)	
	>2.363 & <=5.491	88.17 (86.25, 90.08)	0.891	84.31 (82.79, 85.83)	0.953	79.69 (78.18, 81.20)	0.953	74.06 (72.54, 75.58)	0.817	71.14 (69.42, 72.86)	0.740	70.88 (68.92, 72.83)	0.699	72.91 (70.53, 75.30)	0.685
	>5.491	87.37 (85.50, 89.24)	0.618	83.78 (82.29, 85.26)	0.673	79.47 (77.98, 80.95)	0.777	74.27 (72.79, 75.74)	0.980	71.71 (70.05, 73.38)	0.867	71.82 (69.93, 73.72)	0.762	74.12 (71.80, 76.45)	0.714
MCiOP	<=0.130	88.38 (86.35, 90.41)		84.77 (83.22, 86.32)		80.42 (78.93, 81.91)		75.16 (73.66, 76.66)		72.54 (70.82, 74.26)		72.60 (70.63, 74.57)		74.87 (72.46, 77.28)	
	>0.130	87.52 (85.98, 89.07)	0.426	83.77 (82.68, 84.86)	0.293	79.26 (78.13, 80.38)	0.176	73.75 (72.63, 74.88)	0.107	70.94 (69.65, 72.23)	0.107	70.80 (69.41, 72.18)	0.131	72.93 (71.18, 74.67)	0.154
MiDP	<=0.720	87.94 (86.46, 89.42)		84.21 (83.23, 85.20)		79.73 (78.69, 80.77)		74.28 (73.24, 75.31)		71.50 (70.31, 72.69)		71.38 (70.12, 72.64)		73.52 (71.88, 75.17)	
	>0.720	87.14 (84.55, 89.72)	0.547	83.53 (81.38, 85.67)	0.563	79.18 (77.15, 81.21)	0.613	73.92 (71.86, 75.98)	0.747	71.31 (68.94, 73.67)	0.876	71.35 (68.60, 74.11)	0.983	73.61 (70.39, 76.82)	0.961
∑MBP(i+n)	<=2.860	88.30 (86.43, 90.17)		84.63 (83.12, 86.14)		80.20 (78.68, 81.71)		74.79 (73.27, 76.31)		72.05 (70.32, 73.79)		71.98 (69.99, 73.97)		74.16 (71.73, 76.60)	
	>2.860 & <=5.986	87.05 (85.05, 89.04)	0.302	83.31 (81.78, 84.85)	0.223	78.80 (77.32, 80.29)	0.159	73.29 (71.80, 74.79)	0.138	70.46 (68.75, 72.18)	0.168	70.30 (68.37, 72.23)	0.224	72.42 (70.08, 74.75)	0.268
	>5.986	87.98 (85.90, 90.06)	0.792	84.34 (82.79, 85.88)	0.785	79.93 (78.48, 81.39)	0.789	74.57 (73.11, 76.04)	0.825	71.87 (70.19, 73.55)	0.872	71.83 (69.95, 73.71)	0.915	74.05 (71.78, 76.31)	0.939
∑DEHP	<=7.700	87.64 (85.55, 89.74)		84.08 (82.47, 85.68)		79.80 (78.27, 81.32)		74.62 (73.09, 76.15)		72.06 (70.30, 73.83)		72.15 (70.17, 74.14)		74.43 (72.05, 76.80)	
	>7.700 & <=11.320	87.97 (86.10, 89.84)	0.791	84.25 (82.78, 85.73)	0.873	79.79 (78.32, 81.26)	0.997	74.36 (72.87, 75.85)	0.801	71.60 (69.90, 73.3)	0.686	71.48 (69.56, 73.39)	0.621	73.60 (71.29, 75.91)	0.596
	>11.320	87.74 (85.75, 89.73)	0.937	83.92 (82.40, 85.45)	0.888	79.34 (77.87, 80.81)	0.651	73.74 (72.28, 75.21)	0.387	70.84 (69.17, 72.51)	0.285	70.57 (68.67, 72.47)	0.249	72.59 (70.24, 74.94)	0.241
∑DiNP	<=3.700	87.82 (85.63, 90.01)		84.03 (82.41, 85.65)		79.47 (77.97, 80.96)		73.91 (72.4, 75.42)		71.06 (69.3, 72.81)		70.87 (68.91, 72.84)		72.98 (70.66, 75.3)	
	>3.700 & <=8.292	88.22 (86.30, 90.15)	0.751	84.42 (82.90, 85.93)	0.727	79.84 (78.34, 81.34)	0.709	74.27 (72.76, 75.77)	0.723	71.40 (69.69, 73.11)	0.765	71.20 (69.26, 73.15)	0.812	73.30 (70.91, 75.68)	0.840
	>8.292	87.47 (85.61, 89.34)	0.781	83.90 (82.42, 85.39)	0.912	79.61 (78.12, 81.09)	0.887	74.42 (72.94, 75.90)	0.608	71.88 (70.21, 73.55)	0.468	72.02 (70.12, 73.92)	0.401	74.35 (72.02, 76.68)	0.378
∑DEHPDiNP	<=9.529	88.30 (86.17, 90.44)		84.62 (83.02, 86.22)		80.23 (78.73, 81.72)		74.93 (73.41, 76.45)		72.28 (70.52, 74.05)		72.27 (70.28, 74.26)		74.45 (72.10, 76.81)	
	>9.529 & <=12.850	87.92 (86.00, 89.84)	0.758	84.17 (82.67, 85.68)	0.686	79.71 (78.22, 81.20)	0.605	74.31 (72.80, 75.82)	0.542	71.58 (69.85, 73.31)	0.544	71.48 (69.51, 73.46)	0.572	73.60 (71.21, 76)	0.594
	>12.850	87.30 (85.41, 89.20)	0.421	83.51 (82.01, 85.00)	0.311	78.98 (77.50, 80.46)	0.210	73.49 (72.03, 74.95)	0.151	70.69 (69.05, 72.33)	0.159	70.52 (68.66, 72.37)	0.196	72.58 (70.29, 74.88)	0.227

∑low MWphth	<=5.550	88.58 (86.68, 90.48)		84.80 (83.28, 86.32)		80.23 (78.71, 81.74)		74.60 (73.07, 76.12)		71.66 (69.91, 73.42)		71.39 (69.38, 73.40)		73.44 (71.01, 75.88)	
	>5.550 & <=12.300	87.02 (85.02, 89.03)	0.204	83.47 (81.93, 85.02)	0.222	79.16 (77.67, 80.66)	0.285	73.91 (72.41, 75.40)	0.495	71.28 (69.58, 72.99)	0.742	71.33 (69.40, 73.25)	0.964	73.60 (71.26, 75.95)	0.918
	>12.300	87.64 (85.61, 89.67)	0.442	84.00 (82.47, 85.53)	0.460	79.58 (78.13, 81.04)	0.516	74.18 (72.71, 75.64)	0.676	71.43 (69.75, 73.11)	0.839	71.35 (69.48, 73.23)	0.976	73.54 (71.28, 75.80)	0.951
∑highMWphth	<=9.770	88.14 (86.01, 90.28)		84.45 (82.85, 86.04)		80.03 (78.53, 81.52)		74.69 (73.17, 76.21)		72.02 (70.25, 73.79)		71.98 (69.99, 73.97)		74.14 (71.79, 76.50)	
	>9.770 & <=13.420	87.81 (85.87, 89.74)	0.788	84.15 (82.64, 85.66)	0.793	79.79 (78.30, 81.28)	0.813	74.53 (73.03, 76.04)	0.873	71.92 (70.18, 73.66)	0.932	71.94 (69.96, 73.93)	0.980	74.15 (71.74, 76.56)	0.994
	>13.420	87.54 (85.64, 89.43)	0.625	83.69 (82.20, 85.18)	0.492	79.10 (77.62, 80.58)	0.351	73.52 (72.06, 74.98)	0.240	70.64 (69.01, 72.27)	0.223	70.39 (68.55, 72.23)	0.241	72.41 (70.13, 74.69)	0.261
∑allphth	<=19.780	88.46 (86.51, 90.42)		84.76 (83.22, 86.30)		80.29 (78.78, 81.81)		74.85 (73.31, 76.39)		72.08 (70.29, 73.87)		71.96 (69.9, 74.02)		74.10 (71.62, 76.58)	
	>19.780 & <=30.900	87.45 (85.47, 89.43)	0.409	83.72 (82.19, 85.25)	0.340	79.22 (77.74, 80.71)	0.281	73.73 (72.26, 75.21)	0.270	70.92 (69.25, 72.59)	0.319	70.77 (68.89, 72.64)	0.391	72.88 (70.59, 75.18)	0.442
	>30.900	87.45 (85.46, 89.45)	0.409	83.82 (82.31, 85.34)	0.390	79.45 (77.99, 80.91)	0.394	74.13 (72.66, 75.60)	0.476	71.46 (69.78, 73.14)	0.595	71.45 (69.57, 73.34)	0.716	73.67 (71.40, 75.95)	0.787

Models were adjusted for age at measurement. * Indicates a significant overall effect of the maternal serum phthalate levels on the outcome.

Table S25. Age Adjusted Associations Between Categorized Phthalate Metabolite Levels and Waist Circumference (cm)

		14 year followup		17 year followup		20 year followup		22 year followup	
		Marginal Geometric Mean (95%CI)	p	Marginal Geometric Mean (95%CI)	p	Marginal Geometric Mean (95%CI)	p	Marginal Geometric Mean (95%CI)	p
MEP	<=1.750	75.44 (73.31,77.62)		79.24 (77.24,81.30)		83.36 (81.19,85.58)		86.40 (83.92,88.95)	
	>1.750 & <=5.714	76.49 (74.42,78.62)	0.493	80.05 (78.11,82.04)	0.576	83.88 (81.81,86.01)	0.735	86.71 (84.35,89.13)	0.862
	>5.714	76.77 (74.62,78.97)	0.395	79.75 (77.75,81.80)	0.731	82.93 (80.80,85.12)	0.788	85.27 (82.86,87.74)	0.526
MiBP	<=0.792	74.85 (72.73,77.04)		78.48 (76.49,80.51)		82.38 (80.24,84.58)		85.27 (82.82,87.78)	
	>0.792 & <=1.900	77.96 (75.79,80.20)	**0.048**	81.18 (79.15,83.26)	0.066	84.62 (82.42,86.87)	0.158	87.14 (84.62,89.74)	0.302
	>1.900	75.93 (73.91,78.01)	0.477	79.43 (77.52,81.38)	0.505	83.18 (81.15,85.26)	0.600	85.95 (83.67,88.29)	0.693
MnBP	<=1.999	75.90 (73.71,78.16)		79.26 (77.23,81.35)		82.87 (80.67,85.12)		85.52 (83.00,88.12)	
	>1.999 & <=4.099	77.21 (75.10,79.38)	0.409	80.69 (78.72,82.71)	0.329	84.44 (82.33,86.59)	0.319	87.20 (84.81,89.64)	0.352
	>4.099	75.61 (73.56,77.72)	0.852	79.09 (77.15,81.07)	0.904	82.82 (80.76,84.94)	0.978	85.58 (83.25,87.98)	0.974
MHBP	<=0.220	76.45 (75.02,77.90)		80.06 (78.72,81.43)		83.96 (82.52,85.43)		86.84 (85.20,88.51)	
	>0.220	75.72 (73.32,78.20)	0.616	78.70 (76.50,80.97)	0.309	81.89 (79.57,84.27)	0.144	84.22 (81.62,86.92)	0.104
MBzP	<=0.260	76.22 (74.71,77.75)		79.81 (78.40,81.24)		83.68 (82.17,85.21)		86.53 (84.82,88.27)	
	>0.260	76.30 (74.18,78.48)	0.951	79.44 (77.46,81.47)	0.771	82.81 (80.69,84.99)	0.519	85.28 (82.87,87.76)	0.414
MEHP	<=2.960	77.93 (75.80,80.13)		81.23 (79.23,83.29)		84.77 (82.63,86.96)		87.37 (84.96,89.85)	
	>2.960 & <=5.250	74.50 (72.45,76.61)	**0.025**	78.22 (76.31,80.18)	**0.035**	82.24 (80.17,84.36)	0.099	85.21 (82.84,87.65)	0.216
	>5.250	76.33 (74.20,78.51)	0.303	79.66 (77.67,81.71)	0.282	83.24 (81.10,85.45)	0.330	85.88 (83.44,88.39)	0.401
MECPP	<=0.785	76.74 (74.59,78.96)		80.46 (78.44,82.53)		84.46 (82.27,86.72)		87.42 (84.90,90.02)	
	>0.785 & <=1.241	76.95 (74.72,79.26)	0.895	80.01 (77.93,82.15)	0.766	83.28 (81.07,85.55)	0.463	85.68 (83.18,88.25)	0.343
	>1.241	75.22 (73.24,77.25)	0.312	78.77 (76.92,80.67)	0.232	82.60 (80.62,84.62)	0.220	85.42 (83.19,87.71)	0.250
MCMHP	<=1.153	77.71 (75.54,79.94)		81.03 (79.02,83.10)		84.59 (82.43,86.82)		87.21 (84.73,89.76)	
	>1.153 & <=1.810	76.21 (74.03,78.47)	0.347	79.52 (77.48,81.62)	0.309	83.08 (80.89,85.32)	0.340	85.69 (83.21,88.24)	0.402
	>1.810	74.97 (72.98,77.01)	0.071	78.62 (76.74,80.54)	0.089	82.56 (80.55,84.62)	0.183	85.47 (83.19,87.81)	0.318
MCPP	<=0.190	76.88 (75.27,78.53)		80.49 (78.98,82.03)		84.37 (82.74,86.03)		87.23 (85.37,89.13)	
	>0.190	75.34 (73.46,77.27)	0.227	78.59 (76.84,80.38)	0.111	82.08 (80.22,83.97)	0.072	84.64 (82.55,86.79)	0.074
MINP	<=2.363	77.15 (75.01,79.36)		80.35 (78.34,82.40)		83.76 (81.63,85.96)		86.28 (83.86,88.77)	
	>2.363 & <=5.491	76.23 (74.04,78.48)	0.559	79.72 (77.67,81.82)	0.671	83.47 (81.28,85.71)	0.851	86.23 (83.76,88.78)	0.981
	>5.491	75.41 (73.38,77.49)	0.253	79.06 (77.15,81.01)	0.368	83.00 (80.94,85.11)	0.617	85.91 (83.56,88.32)	0.834
MCiOP	<=0.130	76.04 (73.90,78.24)		79.42 (77.42,81.47)		83.05 (80.90,85.26)		85.73 (83.27,88.26)	
	>0.130	76.34 (74.85,77.87)	0.819	79.82 (78.42,81.25)	0.749	83.56 (82.06,85.09)	0.707	86.32 (84.62,88.05)	0.703
MiDP	<=0.720	75.95 (74.62,77.32)		79.40 (78.14,80.67)		83.10 (81.76,84.46)		85.82 (84.30,87.37)	
	>0.720	77.71 (74.66,80.88)	0.307	81.19 (78.34,84.14)	0.262	84.93 (81.87,88.11)	0.286	87.69 (84.20,91.32)	0.342
∑MBP(i+n)	<=2.860	75.88 (73.70,78.12)		79.25 (77.23,81.33)		82.88 (80.70,85.11)		85.55 (83.06,88.11)	
	>2.860 & <=5.986	76.82 (74.69,79.01)	0.550	80.43 (78.43,82.47)	0.425	84.31 (82.17,86.50)	0.364	87.17 (84.74,89.67)	0.367
	>5.986	76.02 (73.97,78.13)	0.925	79.39 (77.46,81.36)	0.926	83.00 (80.94,85.11)	0.939	85.66 (83.34,88.04)	0.951
∑DEHP	<=7.700	78.26 (76.06,80.51)		81.45 (79.41,83.54)		84.86 (82.68,87.11)		87.37 (84.88,89.93)	
	>7.700 & <=11.320	74.75 (72.67,76.90)	**0.025**	78.29 (76.34,80.30)	**0.031**	82.10 (80.02,84.24)	0.077	84.92 (82.56,87.34)	0.167
	>11.320	75.81 (73.78,77.91)	0.114	79.40 (77.48,81.36)	0.156	83.26 (81.18,85.38)	0.302	86.11 (83.74,88.54)	0.477
∑DiNP	<=3.700	76.33 (74.19,78.53)		79.48 (77.48,81.53)		82.86 (80.72,85.04)		85.33 (82.93,87.81)	
	>3.700 & <=8.292	76.85 (74.68,79.09)	0.741	80.36 (78.33,82.45)	0.550	84.13 (81.96,86.36)	0.416	86.92 (84.45,89.45)	0.375
	>8.292	75.59 (73.55,77.69)	0.629	79.27 (77.35,81.24)	0.882	83.24 (81.17,85.36)	0.804	86.17 (83.81,88.59)	0.633
∑DEHPDiNP	<=9.529	76.67 (74.50,78.89)		79.90 (77.88,81.97)		83.37 (81.21,85.59)		85.92 (83.46,88.45)	
	>9.529 & <=12.850	76.43 (74.27,78.66)	0.882	80.06 (78.03,82.14)	0.917	83.96 (81.79,86.19)	0.709	86.84 (84.37,89.39)	0.608
	>12.850	75.69 (73.65,77.78)	0.524	79.16 (77.25,81.12)	0.608	82.90 (80.86,85.00)	0.762	85.66 (83.35,88.04)	0.884
∑low MWphth	<=5.550	74.80 (72.68,76.97)		78.50 (76.51,80.54)		82.50 (80.33,84.73)		85.45 (82.96,88.02)	
	>5.550 & <=12.300	77.53 (75.36,79.75)	0.081	80.84 (78.83,82.91)	0.109	84.40 (82.25,86.60)	0.228	87.01 (84.58,89.52)	0.387
	>12.300	76.38 (74.33,78.48)	0.298	79.72 (77.80,81.69)	0.393	83.30 (81.26,85.41)	0.600	85.94 (83.63,88.32)	0.781
∑highMWphth	<=9.770	76.84 (74.67,79.08)		80.06 (78.03,82.14)		83.50 (81.33,85.73)		86.03 (83.56,88.56)	
	>9.770 & <=13.420	76.37 (74.22,78.59)	0.767	79.94 (77.92,82.01)	0.936	83.77 (81.60,86.01)	0.862	86.60 (84.12,89.16)	0.750
	>13.420	75.58 (73.55,77.67)	0.413	79.14 (77.23,81.09)	0.522	82.96 (80.93,85.05)	0.728	85.79 (83.49,88.15)	0.890
∑allphth	<=19.780	76.44 (74.24,78.71)		79.67 (77.61,81.79)		83.13 (80.90,85.42)		85.68 (83.13,88.31)	
	>19.780 & <=30.900	75.66 (73.58,77.79)	0.616	79.68 (77.72,81.69)	0.997	84.03 (81.94,86.18)	0.569	87.27 (84.90,89.70)	0.379
	>30.900	76.63 (74.55,78.78)	0.904	79.68 (77.74,81.67)	0.994	82.94 (80.89,85.05)	0.904	85.34 (83.03,87.71)	0.848

Models were adjusted for age at measurement.

Table S26. Age Adjusted Associations Between Categorized Phthalate Metabolite Levels and Alanine Transaminase (ALT, U/L)

		14 year followup		17 year followup		20 year followup		22 year followup	
		Marginal Geometric Mean (95%CI)	p	Marginal Geometric Mean (95%CI)	p	Marginal Geometric Mean (95%CI)	p	Marginal Geometric Mean (95%CI)	p
MEP	<=1.750	18.09 (16.58,19.75)		23.08 (21.22,25.10)		31.71 (28.73,35.00)		33.96 (30.12,38.29)	
	>1.750 &	16.65 (15.32,18.09)	0.163	22.02 (20.30,23.90)	0.377	31.41 (28.54,34.56)	0.884	34.54 (30.75,38.80)	0.839
	>5.714	16.92 (15.53,18.43)	0.267	21.40 (19.66,23.29)	0.162	29.14 (26.40,32.16)	0.210	31.02 (27.45,35.04)	0.284
MiBP	<=0.792	17.56 (16.12,19.14)		22.36 (20.61,24.26)		30.52 (27.67,33.66)		32.70 (28.98,36.90)	
	>0.792 &	17.05 (15.59,18.65)	0.631	22.45 (20.59,24.47)	0.628	31.70 (28.59,35.15)	0.584	34.79 (30.52,39.67)	0.480
	>1.900	16.95 (15.62,18.39)	0.543	21.81 (20.08,23.68)	0.835	30.08 (27.41,33.00)	0.820	32.47 (29.11,36.21)	0.930
MnBP	<=1.999	18.10 (16.57,19.77)		22.89 (21.06,24.88)		31.08 (28.07,34.40)		33.07 (29.10,37.59)	
	>1.999 &	17.07 (15.68,18.57)	0.331	22.30 (20.52,24.23)	0.621	31.29 (28.44,34.43)	0.918	34.10 (30.35,38.31)	0.721
	>4.099	16.53 (15.22,17.97)	0.132	21.38 (19.68,23.24)	0.202	29.71 (26.99,32.70)	0.503	32.13 (28.64,36.05)	0.735
MHBP	<=0.220	17.17 (16.19,18.21)		22.13 (20.76,23.59)		30.61 (28.56,32.81)		33.02 (30.38,35.88)	
	>0.220	17.21 (15.61,18.98)	0.968	22.31 (20.37,24.43)	0.873	31.04 (27.79,34.66)	0.824	33.61 (29.38,38.45)	0.818
MBzP	<=0.260	17.05 (16.03,18.14)		21.95 (20.59,23.39)		30.28 (28.20,32.52)		32.57 (29.84,35.55)	
	>0.260	17.47 (16.00,19.07)	0.649	22.74 (20.84,24.80)	0.453	31.73 (28.68,35.11)	0.422	34.41 (30.53,38.78)	0.451
MEHP	<=2.960	16.57 (15.22,18.05)		22.32 (20.55,24.25)		32.27 (29.27,35.58)		35.43 (31.60,39.73)	*
	>2.960 &	16.86 (15.48,18.36)	0.772	22.41 (20.62,24.35)	0.944	31.95 (28.99,35.20)	0.878	34.73 (30.86,39.10)	0.808
	>5.250	18.14 (16.66,19.76)	0.131	21.92 (20.17,23.83)	0.734	28.35 (25.75,31.21)	0.050	28.76 (25.45,32.50)	0.012
MECPP	<=0.785	16.28 (14.91,17.77)		22.25 (20.43,24.24)		32.68 (29.56,36.12)		37.04 (32.64,42.02)	
	>0.785 &	18.15 (16.64,19.81)	0.077	22.67 (20.81,24.69)	0.738	30.32 (27.43,33.52)	0.276	32.17 (28.50,36.31)	0.103
	>1.241	17.15 (15.81,18.60)	0.378	21.71 (20.05,23.50)	0.635	29.44 (26.84,32.30)	0.114	31.55 (28.23,35.26)	0.053
MCMHP	<=1.153	17.08 (15.67,18.63)		22.68 (20.86,24.66)		32.42 (29.34,35.82)		35.78 (31.64,40.46)	
	>1.153 &	17.13 (15.67,18.71)	0.967	21.81 (19.99,23.79)	0.471	29.85 (27.03,32.97)	0.228	31.96 (28.24,36.16)	0.190
	>1.810	17.31 (15.95,18.79)	0.821	22.02 (20.33,23.85)	0.568	30.10 (27.40,33.06)	0.257	32.19 (28.77,36.01)	0.199
MCPP	<=0.190	15.77 (14.20, 17.51)		20.43 (18.37, 22.71)		28.90 (25.93, 32.23)		31.11 (27.66, 34.99)	
	>0.190	16.19 (14.54, 18.04)	0.568	20.35 (18.28, 22.66)	0.922	27.91 (24.97, 31.19)	0.472	29.38 (25.94, 33.27)	0.355
MINP	<=2.363	16.22 (14.90,17.66)	*	21.27 (19.51,23.18)		30.12 (27.18,33.38)		32.95 (29.18,37.20)	
	>2.363 &	19.05 (17.46,20.79)	0.008	23.18 (21.33,25.20)	0.113	30.42 (27.61,33.50)	0.885	31.52 (27.98,35.50)	0.600
	>5.491	16.59 (15.27,18.02)	0.706	22.08 (20.37,23.93)	0.482	31.76 (28.89,34.92)	0.428	35.14 (31.22,39.54)	0.445
MCiOP	<=0.130	16.04 (14.66,17.54)		21.28 (19.54,23.16)		30.24 (27.35,33.43)		33.29 (29.36,37.74)	
	>0.130	17.69 (16.65,18.79)	0.064	22.62 (21.21,24.12)	0.192	30.95 (28.80,33.27)	0.692	33.17 (30.47,36.12)	0.962
MiDP	<=0.720	17.21 (16.27,18.20)		22.20 (20.90,23.58)		30.72 (28.75,32.83)		33.16 (30.63,35.90)	
	>0.720	17.07 (15.11,19.28)	0.904	22.09 (19.69,24.79)	0.939	30.69 (26.76,35.19)	0.988	33.20 (28.12,39.21)	0.988
∑MBP(i+n)	<=2.860	18.03 (16.53,19.68)		22.74 (20.94,24.71)		30.77 (27.85,34.00)		32.69 (28.85,37.04)	
	>2.860 &	16.93 (15.55,18.42)	0.291	22.34 (20.57,24.27)	0.737	31.68 (28.76,34.88)	0.667	34.78 (30.89,39.16)	0.465
	>5.986	16.68 (15.34,18.15)	0.194	21.47 (19.74,23.35)	0.279	29.66 (26.94,32.65)	0.579	31.97 (28.51,35.85)	0.791
∑DEHP	<=7.700	14.65 (13.01, 16.49)	*	20.06 (17.86, 22.53)		30.00 (26.52, 33.94)		33.31 (29.05, 38.19)	
	>7.700 &	16.06 (14.38, 17.94)	0.100	20.42 (18.30, 22.78)	0.715	28.28 (25.18, 31.75)	0.317	29.73 (26.10, 33.87)	0.128
	>11.320	16.98 (15.11, 19.07)	0.008	20.68 (18.44, 23.20)	0.528	27.42 (24.30, 30.93)	0.133	27.95 (24.37, 32.05)	0.021
∑DiNP	<=3.700	15.90 (14.60,17.33)	*	20.97 (19.22,22.87)		29.91 (26.98,33.16)		32.80 (29.06,37.04)	
	>3.700 &	18.69 (17.15,20.37)	0.007	22.93 (21.13,24.89)	0.097	30.40 (27.61,33.47)	0.812	31.64 (28.07,35.67)	0.671
	>8.292	17.13 (15.77,18.60)	0.209	22.47 (20.73,24.36)	0.194	31.91 (29.03,35.09)	0.336	34.88 (30.97,39.28)	0.468
∑DEHPDiNP	<=9.529	16.46 (15.09,17.96)		21.38 (19.60,23.32)		29.94 (27.01,33.19)		32.29 (28.60,36.46)	
	>9.529 &	17.25 (15.81,18.82)	0.446	22.98 (21.15,24.98)	0.184	33.05 (29.96,36.47)	0.144	36.33 (32.24,40.95)	0.165
	>12.850	17.73 (16.34,19.25)	0.210	22.08 (20.38,23.93)	0.543	29.62 (27.01,32.49)	0.874	30.99 (27.55,34.87)	0.624
∑low MWphth	<=5.550	17.70 (16.21,19.32)		22.49 (20.67,24.47)		30.56 (27.60,33.84)		32.68 (28.88,36.99)	
	>5.550 &	16.38 (15.01,17.87)	0.204	21.83 (20.09,23.72)	0.584	31.17 (28.31,34.32)	0.769	34.52 (30.62,38.92)	0.520
	>12.300	17.45 (16.08,18.95)	0.814	22.24 (20.47,24.16)	0.835	30.31 (27.56,33.33)	0.902	32.48 (28.97,36.42)	0.942
∑highMWphth	<=9.770	16.41 (15.04,17.90)		21.69 (19.89,23.64)		30.91 (27.90,34.25)		33.79 (29.95,38.13)	
	>9.770 &	17.78 (16.32,19.37)	0.185	22.98 (21.13,24.98)	0.287	32.01 (28.98,35.36)	0.608	34.43 (30.49,38.88)	0.827
	>13.420	17.36 (15.98,18.86)	0.344	21.87 (20.19,23.69)	0.874	29.68 (27.06,32.54)	0.539	31.33 (27.86,35.22)	0.364
∑allphth	<=19.780	17.32 (15.85,18.92)		22.51 (20.64,24.54)		31.38 (28.23,34.87)		34.05 (29.96,38.70)	
	>19.780	16.81 (15.46,18.29)	0.625	22.10 (20.38,23.96)	0.733	31.16 (28.40,34.18)	0.918	34.09 (30.38,38.24)	0.990
	>30.900	17.44 (16.03,18.97)	0.907	21.99 (20.23,23.91)	0.672	29.73 (27.01,32.72)	0.430	31.56 (28.10,35.45)	0.377

Models were adjusted for age at measurement. * Indicates a significant overall effect of the maternal serum phthalate levels on the outcome.

Table S27. Age Adjusted Associations Between Categorized Phthalate Metabolite Levels and Gamma-Glutamyl Transferase (GGT, U/L)

		14 year followup		17 year followup		20 year followup		22 year followup	
		Marginal Geometric Mean (95%CI)	p	Marginal Geometric Mean (95%CI)	p	Marginal Geometric Mean (95%CI)	p	Marginal Geometric Mean (95%CI)	p
MEP	<=1.750	12.60 (11.69,13.60)		15.13 (14.06,16.28)		18.25 (16.68,19.97)		20.84 (18.69,23.24)	
	>1.750 & <=5.714	11.68 (10.86,12.56)	0.153	14.67 (13.67,15.73)	0.552	18.54 (17.00,20.21)	0.804	21.88 (19.71,24.30)	0.527
	>5.714	11.95 (11.09,12.88)	0.327	14.49 (13.47,15.59)	0.419	17.67 (16.14,19.34)	0.622	20.34 (18.22,22.69)	0.756
MiBP	<=0.792	12.35 (11.47,13.31)		15.27 (14.22,16.40)		18.98 (17.39,20.73)		22.15 (19.90,24.66)	
	>0.792 & <=1.900	12.12 (11.21,13.10)	0.727	14.71 (13.65,15.87)	0.484	17.96 (16.34,19.75)	0.401	20.69 (18.41,23.25)	0.398
	>1.900	11.74 (10.93,12.62)	0.338	14.32 (13.36,15.35)	0.207	17.56 (16.14,19.10)	0.209	20.29 (18.34,22.45)	0.242
MnBP	<=1.999	12.67 (11.74,13.67)		15.60 (14.52,16.77)		19.32 (17.65,21.16)		22.48 (20.10,25.15)	
	>1.999 & <=4.099	11.73 (10.90,12.63)	0.155	14.41 (13.42,15.47)	0.124	17.79 (16.31,19.41)	0.199	20.66 (18.59,22.96)	0.282
	>4.099	11.81 (10.98,12.71)	0.193	14.33 (13.34,15.38)	0.098	17.46 (16.01,19.04)	0.114	20.10 (18.10,22.32)	0.152
MHBP	<=0.220	11.97 (11.39,12.58)		14.81 (14.12,15.55)		18.44 (17.38,19.57)		21.53 (20.04,23.14)	
	>0.220	12.36 (11.35,13.45)	0.527	14.62 (13.48,15.86)	0.785	17.38 (15.73,19.20)	0.316	19.64 (17.39,22.18)	0.201
MBzP	<=0.260	12.11 (11.50,12.75)		14.71 (13.99,15.47)		17.96 (16.88,19.11)		20.69 (19.18,22.32)	
	>0.260	11.96 (11.08,12.92)	0.796	14.86 (13.80,16.01)	0.816	18.58 (16.97,20.35)	0.544	21.77 (19.50,24.30)	0.458
MEHP	<=2.960	12.36 (11.48,13.31)		15.03 (13.99,16.15)		18.37 (16.83,20.04)	*	21.18 (19.07,23.52)	*
	>2.960 & <=5.250	11.91 (11.06,12.83)	0.489	15.28 (14.23,16.41)	0.748	19.74 (18.09,21.53)	0.254	23.66 (21.28,26.30)	0.145
	>5.250	11.94 (11.08,12.87)	0.523	14.01 (13.05,15.04)	0.175	16.51 (15.13,18.02)	0.090	18.55 (16.67,20.63)	0.083
MECPP	<=0.785	11.59 (10.74,12.50)	*	14.68 (13.65,15.79)		18.73 (17.10,20.51)		22.26 (19.91,24.88)	
	>0.785 & <=1.241	13.15 (12.19,14.18)	0.021	15.80 (14.68,17.00)	0.164	19.08 (17.44,20.88)	0.774	21.82 (19.55,24.34)	0.803
	>1.241	11.58 (10.80,12.41)	0.987	14.00 (13.08,14.98)	0.344	17.01 (15.65,18.48)	0.125	19.53 (17.66,21.60)	0.088
MCMHP	<=1.153	12.45 (11.55,13.42)		15.12 (14.07,16.26)		18.46 (16.87,20.21)		21.27 (19.04,23.76)	
	>1.153 & <=1.810	12.02 (11.12,12.99)	0.520	14.80 (13.74,15.95)	0.686	18.33 (16.73,20.09)	0.915	21.34 (19.09,23.85)	0.968
	>1.810	11.76 (10.95,12.62)	0.275	14.39 (13.43,15.42)	0.333	17.72 (16.28,19.28)	0.514	20.53 (18.53,22.74)	0.646
MCPP	<=0.190	12.03 (11.38,12.73)		14.93 (14.14,15.77)		18.64 (17.42,19.95)		21.81 (20.09,23.69)	
	>0.190	12.11 (11.33,12.95)	0.883	14.54 (13.65,15.50)	0.539	17.55 (16.24,18.97)	0.253	20.05 (18.25,22.04)	0.188
MINP	<=2.363	11.59 (10.75,12.49)		14.52 (13.49,15.63)		18.31 (16.72,20.06)		21.59 (19.32,24.12)	
	>2.363 & <=5.491	12.18 (11.28,13.14)	0.363	14.64 (13.62,15.74)	0.873	17.70 (16.21,19.32)	0.598	20.25 (18.20,22.52)	0.414
	>5.491	12.43 (11.57,13.36)	0.185	15.11 (14.10,16.21)	0.439	18.48 (16.95,20.14)	0.888	21.31 (19.19,23.66)	0.868
MCiOP	<=0.130	12.06 (11.15,13.04)		14.75 (13.69,15.89)		18.14 (16.55,19.89)		21.01 (18.78,23.51)	
	>0.130	12.06 (11.46,12.70)	0.995	14.76 (14.04,15.52)	0.987	18.16 (17.08,19.32)	0.983	21.04 (19.52,22.68)	0.983
MiDP	<=0.720	11.87 (11.33,12.44)		14.63 (13.98,15.31)		18.12 (17.14,19.17)		21.10 (19.71,22.58)	
	>0.720	13.08 (11.75,14.56)	0.105	15.47 (13.94,17.18)	0.334	18.39 (16.18,20.91)	0.837	20.79 (17.80,24.28)	0.864
∑MBP(i+n)	<=2.860	12.74 (11.82,13.74)		15.49 (14.41,16.64)		18.92 (17.30,20.70)		21.81 (19.53,24.35)	
	>2.860 & <=5.986	11.62 (10.80,12.51)	0.087	14.53 (13.54,15.61)	0.220	18.29 (16.75,19.97)	0.596	21.52 (19.34,23.96)	0.866
	>5.986	11.87 (11.03,12.78)	0.187	14.31 (13.32,15.36)	0.125	17.33 (15.88,18.90)	0.167	19.85 (17.87,22.05)	0.225
∑DEHP	<=7.700	12.26 (11.37,13.23)		15.33 (14.25,16.50)		19.29 (17.62,21.11)		22.70 (20.33,25.34)	
	>7.700 & <=11.320	12.26 (11.37,13.21)	0.994	14.92 (13.88,16.02)	0.599	18.25 (16.72,19.91)	0.387	21.06 (18.94,23.40)	0.334
	>11.320	11.71 (10.89,12.59)	0.389	14.11 (13.16,15.14)	0.110	17.10 (15.68,18.65)	0.060	19.60 (17.63,21.78)	0.059
∑DiNP	<=3.700	11.34 (10.52,12.24)		14.15 (13.13,15.24)		17.76 (16.20,19.47)		20.86 (18.65,23.33)	
	>3.700 & <=8.292	12.27 (11.39,13.22)	0.146	14.90 (13.88,15.99)	0.326	18.18 (16.66,19.83)	0.717	20.94 (18.83,23.28)	0.964
	>8.292	12.55 (11.68,13.48)	0.058	15.19 (14.17,16.29)	0.173	18.48 (16.95,20.16)	0.535	21.24 (19.12,23.60)	0.817
∑DEHPDiNP	<=9.529	11.65 (10.80,12.58)		14.47 (13.43,15.59)		18.08 (16.49,19.83)		21.17 (18.93,23.68)	
	>9.529 & <=12.850	12.28 (11.39,13.24)	0.338	15.10 (14.05,16.22)	0.424	18.67 (17.09,20.38)	0.625	21.69 (19.49,24.15)	0.759
	>12.850	12.23 (11.38,13.14)	0.365	14.70 (13.71,15.76)	0.763	17.76 (16.31,19.34)	0.780	20.31 (18.31,22.53)	0.592
∑low MWphth	<=5.550	12.25 (11.35,13.22)		14.87 (13.82,16.01)		18.15 (16.56,19.89)		20.91 (18.69,23.38)	
	>5.550 & <=12.300	12.31 (11.42,13.27)	0.930	15.25 (14.19,16.39)	0.632	19.01 (17.41,20.75)	0.477	22.22 (19.96,24.72)	0.441
	>12.300	11.67 (10.86,12.54)	0.362	14.20 (13.24,15.23)	0.371	17.37 (15.94,18.93)	0.493	20.04 (18.06,22.24)	0.588
∑highMWphth	<=9.770	11.73 (10.87,12.66)		14.63 (13.58,15.76)		18.35 (16.75,20.12)		21.55 (19.28,24.09)	
	>9.770 & <=13.420	12.20 (11.32,13.15)	0.474	14.92 (13.88,16.04)	0.712	18.34 (16.77,20.06)	0.993	21.24 (19.04,23.69)	0.852
	>13.420	12.24 (11.38,13.16)	0.433	14.73 (13.74,15.80)	0.892	17.83 (16.38,19.41)	0.650	20.41 (18.41,22.62)	0.481
∑allphth	<=19.780	12.52 (11.59,13.53)		15.28 (14.17,16.47)		18.75 (17.06,20.60)		21.68 (19.31,24.33)	
	>19.780 & <=30.900	11.46 (10.66,12.32)	0.101	14.41 (13.44,15.45)	0.263	18.24 (16.76,19.85)	0.669	21.55 (19.45,23.88)	0.941
	>30.900	12.29 (11.42,13.22)	0.730	14.65 (13.64,15.73)	0.425	17.55 (16.09,19.14)	0.312	19.95 (17.95,22.16)	0.296

Models were adjusted for age at measurement. * Indicates a significant overall effect of the maternal serum phthalate levels on the outcome.

Table S28. Age Adjusted Associations Between Categorized Phthalate Metabolite Levels and Aspartate Transaminase (AST, U/L)

		17 year followup		20 year followup		22 year followup	
		Marginal Geometric Mean (95%CI)	p	Marginal Geometric Mean (95%CI)	p	Marginal Geometric Mean (95%CI)	p
MEP	<=1.750	26.12 (24.45,27.90)		27.80 (26.20,29.51)		29.06 (26.71,31.63)	
	>1.750 & <=5.714	26.49 (24.82,28.27)	0.765	27.63 (26.08,29.26)	0.879	28.46 (26.21,30.91)	0.727
	>5.714	25.92 (24.19,27.77)	0.877	27.22 (25.62,28.91)	0.622	28.17 (25.84,30.72)	0.614
MiBP	<=0.792	25.74 (24.14,27.43)		27.24 (25.67,28.90)		28.36 (26.00,30.94)	
	>0.792 & <=1.900	27.65 (25.77,29.66)	0.139	28.48 (26.71,30.37)	0.318	29.09 (26.47,31.96)	0.699
	>1.900	25.42 (23.81,27.12)	0.787	27.08 (25.63,28.61)	0.884	28.32 (26.26,30.55)	0.982
MnBP	<=1.999	25.99 (24.29,27.80)		26.82 (25.19,28.55)		27.42 (24.99,30.10)	
	>1.999 & <=4.099	27.44 (25.69,29.30)	0.259	28.35 (26.77,30.02)	0.201	29.01 (26.74,31.46)	0.373
	>4.099	25.19 (23.59,26.89)	0.513	27.36 (25.84,28.97)	0.645	29.01 (26.77,31.45)	0.370
MHBP	<=0.220	25.99 (24.82,27.20)		27.48 (26.41,28.59)		28.59 (27.02,30.26)	
	>0.220	26.66 (24.82,28.63)	0.556	27.73 (25.94,29.66)	0.817	28.53 (25.91,31.40)	0.967
MBzP	<=0.260	26.27 (25.09,27.49)		27.36 (26.25,28.52)		28.16 (26.53,29.90)	
	>0.260	25.98 (24.19,27.92)	0.804	27.92 (26.27,29.66)	0.591	29.37 (27.01,31.95)	0.424
MEHP	<=2.960	27.31 (25.56,29.17)		28.10 (26.52,29.78)		28.68 (26.46,31.08)	
	>2.960 & <=5.250	26.26 (24.59,28.04)	0.411	28.18 (26.56,29.89)	0.948	29.62 (27.22,32.23)	0.587
	>5.250	25.01 (23.38,26.75)	0.068	26.45 (24.91,28.09)	0.156	27.53 (25.20,30.07)	0.503
MECPP	<=0.785	25.46 (23.69,27.35)		28.50 (26.79,30.32)		30.87 (28.20,33.80)	
	>0.785 & <=1.241	26.52 (24.77,28.39)	0.419	27.28 (25.69,28.98)	0.323	27.84 (25.58,30.31)	0.103
	>1.241	26.34 (24.77,28.02)	0.478	27.11 (25.66,28.64)	0.237	27.66 (25.61,29.88)	0.071
MCMHP	<=1.153	26.24 (24.52,28.07)		28.22 (26.56,29.99)		29.72 (27.23,32.44)	
	>1.153 & <=1.810	26.68 (24.82,28.68)	0.739	27.74 (26.09,29.49)	0.694	28.51 (26.13,31.11)	0.511
	>1.810	25.75 (24.21,27.39)	0.692	26.87 (25.42,28.41)	0.244	27.70 (25.61,29.96)	0.240
MCPP	<=0.190	26.52 (25.21,27.89)		28.14 (26.90,29.44)		29.35 (27.52,31.30)	
	>0.190	25.75 (24.27,27.31)	0.457	26.80 (25.46,28.22)	0.165	27.58 (25.60,29.71)	0.215
MINP	<=2.363	27.83 (25.94,29.85)		28.08 (26.40,29.86)		28.26 (25.95,30.78)	
	>2.363 & <=5.491	25.74 (24.10,27.50)	0.114	27.27 (25.72,28.91)	0.498	28.40 (26.11,30.89)	0.935
	>5.491	25.29 (23.73,26.94)	0.048	27.50 (25.96,29.13)	0.626	29.18 (26.85,31.72)	0.598
MCiOP	<=0.130	25.73 (24.03,27.55)		27.82 (26.15,29.59)		29.40 (26.88,32.16)	
	>0.130	26.38 (25.19,27.64)	0.550	27.45 (26.35,28.60)	0.728	28.24 (26.64,29.93)	0.459
MiDP	<=0.720	26.14 (25.08,27.26)		27.54 (26.53,28.59)		28.57 (27.08,30.15)	
	>0.720	26.40 (23.86,29.20)	0.862	27.65 (25.39,30.10)	0.934	28.57 (25.41,32.12)	0.998
∑MBP(i+n)	<=2.860	26.15 (24.48,27.95)		27.08 (25.47,28.79)		27.76 (25.36,30.38)	
	>2.860 & <=5.986	26.82 (25.11,28.66)	0.597	28.13 (26.53,29.82)	0.381	29.09 (26.76,31.62)	0.456
	>5.986	25.57 (23.91,27.35)	0.638	27.37 (25.84,28.99)	0.806	28.72 (26.51,31.11)	0.580
∑DEHP	<=7.700	27.07 (25.27,29.00)		28.51 (26.84,30.29)		29.58 (27.18,32.20)	
	>7.700 & <=11.320	25.93 (24.26,27.71)	0.380	27.81 (26.24,29.47)	0.560	29.23 (26.92,31.73)	0.840
	>11.320	25.69 (24.09,27.40)	0.277	26.42 (24.92,28.01)	0.076	26.96 (24.76,29.36)	0.129
∑DiNP	<=3.700	27.24 (25.35,29.27)		28.16 (26.47,29.95)		28.82 (26.49,31.36)	
	>3.700 & <=8.292	25.85 (24.23,27.59)	0.291	27.08 (25.54,28.72)	0.371	27.99 (25.71,30.48)	0.632
	>8.292	25.69 (24.10,27.38)	0.232	27.57 (26.03,29.21)	0.627	28.99 (26.67,31.52)	0.923
∑DEHPDiNP	<=9.529	27.51 (25.61,29.54)		27.83 (26.16,29.60)		28.05 (25.77,30.53)	
	>9.529 & <=12.850	25.88 (24.21,27.67)	0.221	28.36 (26.72,30.10)	0.664	30.26 (27.79,32.95)	0.216
	>12.850	25.47 (23.93,27.11)	0.111	26.73 (25.26,28.29)	0.348	27.66 (25.47,30.05)	0.817
∑low MWphth	<=5.550	26.59 (24.87,28.43)		27.81 (26.15,29.58)		28.71 (26.29,31.36)	
	>5.550 & <=12.300	26.37 (24.66,28.19)	0.862	27.72 (26.14,29.40)	0.941	28.73 (26.37,31.29)	0.995
	>12.300	25.60 (23.97,27.35)	0.431	27.15 (25.64,28.74)	0.571	28.29 (26.11,30.66)	0.809
∑highMWphth	<=9.770	27.55 (25.66,29.58)		28.32 (26.63,30.11)		28.87 (26.53,31.42)	
	>9.770 & <=13.420	25.77 (24.09,27.56)	0.180	27.56 (25.94,29.28)	0.538	28.91 (26.52,31.51)	0.985
	>13.420	25.53 (23.99,27.18)	0.114	27.00 (25.52,28.57)	0.264	28.10 (25.88,30.51)	0.652
∑allphth	<=19.780	27.02 (25.14,29.05)		27.98 (26.27,29.80)		28.67 (26.28,31.29)	
	>19.780 & <=30.900	26.52 (24.83,28.32)	0.705	27.79 (26.29,29.37)	0.872	28.72 (26.57,31.04)	0.979
	>30.900	25.11 (23.42,26.92)	0.150	26.95 (25.46,28.53)	0.389	28.34 (26.21,30.66)	0.846

Models were adjusted for age at measurement.

Table S29. Age Adjusted Associations Between Categorized Phthalate Metabolite Levels and High-Density Lipoprotein Cholesterol (HDL-C, mmol/L)

		14 year followup		17 year followup		20 year followup		22 year followup	
		Marginal Geometric Mean (95%CI)	p	Marginal Geometric Mean (95%CI)	p	Marginal Geometric Mean (95%CI)	p	Marginal Geometric Mean (95%CI)	p
MEP	<=1.750	1.35 (1.29,1.42)		1.22 (1.16,1.27)		1.21 (1.16,1.27)		1.22 (1.16,1.28)	
	>1.750 & <=5.714	1.30 (1.24,1.36)	0.228	1.19 (1.14,1.24)	0.443	1.21 (1.16,1.26)	0.907	1.23 (1.17,1.29)	0.776
	>5.714	1.32 (1.26,1.39)	0.499	1.20 (1.15,1.26)	0.739	1.22 (1.17,1.27)	0.905	1.23 (1.17,1.29)	0.704
MiBP	<=0.792	1.30 (1.24,1.36)		1.19 (1.14,1.24)		1.21 (1.16,1.26)		1.22 (1.16,1.28)	
	>0.792 & <=1.900	1.33 (1.27,1.40)	0.502	1.20 (1.14,1.25)	0.807	1.20 (1.14,1.25)	0.774	1.20 (1.13,1.26)	0.570
	>1.900	1.34 (1.28,1.40)	0.335	1.22 (1.17,1.27)	0.307	1.24 (1.19,1.29)	0.369	1.25 (1.20,1.31)	0.460
MnBP	<=1.999	1.30 (1.24,1.36)		1.18 (1.14,1.24)		1.20 (1.15,1.26)		1.22 (1.16,1.28)	
	>1.999 & <=4.099	1.32 (1.26,1.39)	0.559	1.20 (1.15,1.25)	0.734	1.20 (1.15,1.25)	0.995	1.21 (1.15,1.27)	0.834
	>4.099	1.35 (1.29,1.42)	0.208	1.23 (1.18,1.28)	0.219	1.24 (1.19,1.29)	0.339	1.25 (1.19,1.31)	0.484
MHBP	<=0.220	1.32 (1.28,1.36)		1.20 (1.16,1.23)		1.21 (1.17,1.25)		1.22 (1.18,1.26)	
	>0.220	1.35 (1.28,1.43)	0.387	1.22 (1.17,1.28)	0.427	1.23 (1.17,1.29)	0.575	1.24 (1.17,1.30)	0.715
MBzP	<=0.260	1.32 (1.28,1.37)		1.20 (1.16,1.24)		1.21 (1.17,1.25)		1.22 (1.18,1.26)	
	>0.260	1.33 (1.27,1.40)	0.783	1.21 (1.16,1.26)	0.726	1.22 (1.17,1.28)	0.711	1.23 (1.17,1.29)	0.729
MEHP	<=2.960	1.30 (1.24,1.36)		1.17 (1.12,1.22)		1.16 (1.12,1.22)	*	1.17 (1.12,1.22)	*
	>2.960 & <=5.250	1.32 (1.26,1.38)	0.663	1.20 (1.15,1.25)	0.296	1.22 (1.17,1.27)	0.120	1.24 (1.18,1.30)	0.087
	>5.250	1.36 (1.30,1.42)	0.162	1.24 (1.19,1.29)	0.033	1.26 (1.20,1.31)	0.010	1.27 (1.21,1.34)	0.010
MECPP	<=0.785	1.31 (1.25,1.38)		1.19 (1.14,1.25)		1.21 (1.16,1.26)		1.22 (1.16,1.29)	
	>0.785 & <=1.241	1.31 (1.25,1.37)	0.936	1.19 (1.14,1.24)	0.942	1.21 (1.16,1.26)	0.961	1.22 (1.16,1.28)	0.975
	>1.241	1.35 (1.29,1.41)	0.399	1.22 (1.17,1.27)	0.484	1.22 (1.17,1.27)	0.689	1.23 (1.18,1.29)	0.855
MCMHP	<=1.153	1.30 (1.24,1.36)		1.19 (1.14,1.24)		1.22 (1.17,1.27)		1.24 (1.18,1.31)	
	>1.153 & <=1.810	1.30 (1.24,1.37)	0.918	1.18 (1.13,1.23)	0.785	1.20 (1.14,1.25)	0.519	1.21 (1.15,1.27)	0.422
	>1.810	1.37 (1.31,1.43)	0.084	1.23 (1.18,1.28)	0.272	1.23 (1.18,1.28)	0.852	1.23 (1.17,1.28)	0.707
MCPP	<=0.190	1.34 (1.29,1.39)		1.22 (1.18,1.26)		1.24 (1.20,1.28)		1.25 (1.20,1.30)	
	>0.190	1.31 (1.25,1.36)	0.378	1.18 (1.14,1.23)	0.165	1.19 (1.14,1.23)	0.088	1.19 (1.14,1.24)	0.083
MINP	<=2.363	1.35 (1.28,1.41)		1.22 (1.17,1.27)		1.23 (1.18,1.28)		1.24 (1.18,1.30)	
	>2.363 & <=5.491	1.34 (1.28,1.41)	0.925	1.21 (1.16,1.26)	0.703	1.21 (1.16,1.26)	0.524	1.21 (1.15,1.27)	0.465
	>5.491	1.29 (1.24,1.35)	0.206	1.18 (1.14,1.23)	0.311	1.21 (1.16,1.26)	0.586	1.23 (1.17,1.29)	0.830
MCiOP	<=0.130	1.33 (1.27,1.40)		1.20 (1.15,1.26)		1.21 (1.16,1.27)		1.22 (1.16,1.28)	
	>0.130	1.32 (1.28,1.37)	0.802	1.20 (1.17,1.24)	0.948	1.22 (1.18,1.25)	0.874	1.23 (1.19,1.27)	0.781
MiDP	<=0.720	1.33 (1.29,1.37)		1.20 (1.17,1.24)		1.21 (1.18,1.25)		1.22 (1.18,1.26)	
	>0.720	1.32 (1.24,1.41)	0.936	1.21 (1.14,1.28)	0.807	1.23 (1.16,1.31)	0.567	1.25 (1.17,1.34)	0.473
∑MBP(i+n)	<=2.860	1.30 (1.24,1.36)		1.19 (1.14,1.24)		1.20 (1.15,1.26)		1.22 (1.16,1.28)	
	>2.860 & <=5.986	1.32 (1.26,1.38)	0.721	1.19 (1.14,1.24)	0.939	1.19 (1.14,1.25)	0.798	1.2 (1.14,1.26)	0.668
	>5.986	1.36 (1.30,1.42)	0.183	1.23 (1.18,1.29)	0.177	1.24 (1.19,1.30)	0.267	1.25 (1.20,1.31)	0.390
∑DEHP	<=7.700	1.30 (1.24,1.36)		1.17 (1.13,1.23)		1.19 (1.13,1.24)		1.20 (1.14,1.26)	
	>7.700 & <=11.320	1.31 (1.25,1.37)	0.791	1.19 (1.14,1.24)	0.757	1.20 (1.15,1.25)	0.763	1.21 (1.15,1.27)	0.789
	>11.320	1.37 (1.31,1.43)	0.082	1.25 (1.20,1.30)	0.041	1.26 (1.21,1.31)	0.047	1.27 (1.21,1.33)	0.079
∑DiNP	<=3.700	1.36 (1.30,1.43)		1.23 (1.18,1.28)		1.23 (1.18,1.29)		1.24 (1.18,1.30)	
	>3.700 & <=8.292	1.33 (1.27,1.39)	0.461	1.20 (1.15,1.25)	0.399	1.20 (1.15,1.25)	0.420	1.21 (1.15,1.27)	0.480
	>8.292	1.29 (1.24,1.35)	0.126	1.19 (1.14,1.24)	0.224	1.21 (1.16,1.26)	0.525	1.23 (1.17,1.29)	0.818
∑DEHPDiNP	<=9.529	1.32 (1.26,1.38)		1.19 (1.14,1.24)		1.19 (1.14,1.24)		1.20 (1.14,1.25)	
	>9.529 & <=12.850	1.32 (1.26,1.38)	0.964	1.19 (1.14,1.24)	0.973	1.19 (1.14,1.24)	0.906	1.20 (1.15,1.26)	0.875
	>12.850	1.34 (1.28,1.40)	0.577	1.23 (1.18,1.28)	0.208	1.25 (1.20,1.31)	0.069	1.28 (1.22,1.34)	0.047
∑low MWphth	<=5.550	1.33 (1.27,1.39)		1.20 (1.15,1.26)		1.21 (1.16,1.27)		1.22 (1.16,1.28)	
	>5.550 & <=12.300	1.31 (1.25,1.38)	0.730	1.18 (1.14,1.23)	0.572	1.19 (1.14,1.24)	0.480	1.19 (1.14,1.25)	0.471
	>12.300	1.33 (1.28,1.40)	0.892	1.22 (1.17,1.27)	0.636	1.24 (1.19,1.29)	0.442	1.26 (1.20,1.32)	0.383
∑highMWphth	<=9.770	1.31 (1.25,1.37)		1.18 (1.13,1.23)		1.19 (1.14,1.24)		1.20 (1.14,1.26)	
	>9.770 & <=13.420	1.33 (1.27,1.39)	0.649	1.19 (1.15,1.25)	0.714	1.19 (1.14,1.25)	0.845	1.20 (1.14,1.26)	0.941
	>13.420	1.34 (1.28,1.40)	0.475	1.23 (1.18,1.28)	0.187	1.25 (1.20,1.30)	0.078	1.27 (1.22,1.34)	0.062
∑allphth	<=19.780	1.31 (1.25,1.38)		1.18 (1.13,1.23)		1.18 (1.13,1.23)		1.18 (1.12,1.24)	
	>19.780 & <=30.900	1.33 (1.27,1.39)	0.741	1.21 (1.16,1.26)	0.426	1.22 (1.17,1.27)	0.242	1.24 (1.18,1.29)	0.202
	>30.900	1.34 (1.28,1.40)	0.571	1.22 (1.17,1.27)	0.263	1.23 (1.18,1.29)	0.126	1.25 (1.19,1.31)	0.104

Models were adjusted for age at measurement. * Indicates a significant overall effect of the maternal serum phthalate levels on the outcome.

Table S30. Age Adjusted Associations Between Categorized Phthalate Metabolite Levels and Low-Density Lipoprotein Cholesterol (LDL-C, mmol/L)

		14 year followup		17 year followup		20 year followup		22 year followup	
		Marginal Mean (95%CI)	p	Marginal Mean (95%CI)	p	Marginal Mean (95%CI)	p	Marginal Mean (95%CI)	p
MEP	<=1.750	2.36 (2.22,2.50)		2.36 (2.23,2.49)		2.55 (2.41,2.70)		2.81 (2.64,2.98)	
	>1.750 & <=5.714	2.22 (2.09,2.36)	0.191	2.24 (2.11,2.36)	0.200	2.44 (2.30,2.58)	0.292	2.71 (2.54,2.87)	0.396
	>5.714	2.22 (2.09,2.36)	0.166	2.23 (2.09,2.36)	0.161	2.42 (2.28,2.57)	0.222	2.68 (2.51,2.85)	0.291
MiBP	<=0.792	2.28 (2.14,2.41)		2.25 (2.12,2.38)		2.42 (2.28,2.56)		2.65 (2.49,2.82)	
	>0.792 & <=1.900	2.20 (2.06,2.34)	0.444	2.21 (2.08,2.35)	0.665	2.42 (2.27,2.57)	0.986	2.68 (2.50,2.86)	0.832
	>1.900	2.32 (2.19,2.45)	0.640	2.35 (2.22,2.48)	0.299	2.56 (2.43,2.70)	0.158	2.83 (2.68,2.99)	0.127
MnBP	<=1.999	2.27 (2.13,2.41)		2.27 (2.14,2.40)		2.45 (2.31,2.60)		2.70 (2.53,2.88)	
	>1.999 & <=4.099	2.22 (2.08,2.36)	0.570	2.24 (2.11,2.37)	0.758	2.45 (2.31,2.59)	0.996	2.72 (2.56,2.88)	0.879
	>4.099	2.31 (2.17,2.45)	0.696	2.31 (2.18,2.45)	0.602	2.51 (2.37,2.65)	0.578	2.76 (2.60,2.92)	0.597
MHBP	<=0.220	2.25 (2.16,2.35)		2.27 (2.18,2.35)		2.47 (2.38,2.56)		2.73 (2.63,2.84)	
	>0.220	2.31 (2.13,2.48)	0.591	2.30 (2.13,2.46)	0.747	2.48 (2.29,2.66)	0.945	2.72 (2.52,2.93)	0.936
MBzP	<=0.260	2.26 (2.16,2.35)		2.27 (2.18,2.35)		2.47 (2.37,2.56)		2.73 (2.61,2.85)	
	>0.260	2.29 (2.13,2.45)	0.678	2.29 (2.14,2.44)	0.756	2.48 (2.32,2.64)	0.875	2.73 (2.56,2.91)	0.953
MEHP	<=2.960	2.27 (2.12,2.42)		2.29 (2.16,2.42)		2.50 (2.36,2.64)		2.77 (2.61,2.92)	
	>2.960 & <=5.250	2.28 (2.14,2.41)	0.957	2.29 (2.16,2.42)	0.985	2.49 (2.35,2.64)	0.929	2.75 (2.58,2.92)	0.903
	>5.250	2.25 (2.12,2.39)	0.863	2.24 (2.11,2.38)	0.608	2.42 (2.28,2.57)	0.436	2.66 (2.50,2.83)	0.383
MECPP	<=0.785	2.24 (2.10,2.38)	*	2.26 (2.15,2.37)	*	2.47 (2.35,2.58)		2.74 (2.59,2.88)	
	>0.785 & <=1.241	2.42 (2.28,2.56)	0.071	2.42 (2.28,2.56)	0.068	2.61 (2.45,2.77)	0.145	2.87 (2.69,3.05)	0.259
	>1.241	2.16 (2.02,2.30)	0.410	2.16 (2.03,2.29)	0.251	2.35 (2.21,2.50)	0.210	2.61 (2.44,2.77)	0.232
MCMHP	<=1.153	2.27 (2.15,2.39)		2.26 (2.13,2.38)		2.43 (2.29,2.57)		2.67 (2.51,2.83)	
	>1.153 & <=1.810	2.27 (2.10,2.44)	1.000	2.33 (2.18,2.47)	0.465	2.57 (2.42,2.72)	0.176	2.86 (2.69,3.04)	0.110
	>1.810	2.26 (2.12,2.39)	0.846	2.24 (2.12,2.37)	0.868	2.42 (2.28,2.56)	0.911	2.67 (2.51,2.82)	0.940
MCPP	<=0.190	2.27 (2.17,2.38)		2.29 (2.19,2.38)		2.49 (2.39,2.59)		2.75 (2.63,2.87)	
	>0.190	2.26 (2.13,2.39)	0.856	2.26 (2.13,2.38)	0.699	2.45 (2.31,2.58)	0.598	2.70 (2.54,2.85)	0.569
MINP	<=2.363	2.33 (2.18,2.48)		2.34 (2.19,2.48)		2.53 (2.36,2.70)		2.79 (2.60,2.98)	
	>2.363 & <=5.491	2.19 (2.05,2.33)	0.145	2.18 (2.06,2.29)	0.087	2.36 (2.23,2.48)	0.096	2.60 (2.45,2.75)	0.129
	>5.491	2.28 (2.15,2.41)	0.577	2.31 (2.18,2.44)	0.764	2.53 (2.39,2.67)	0.990	2.80 (2.65,2.96)	0.885
MCiOP	<=0.130	2.31 (2.16,2.47)		2.33 (2.20,2.46)		2.54 (2.41,2.68)		2.81 (2.66,2.96)	
	>0.130	2.25 (2.15,2.34)	0.471	2.25 (2.15,2.34)	0.290	2.44 (2.34,2.54)	0.222	2.69 (2.57,2.82)	0.229
MiDP	<=0.720	2.26 (2.17,2.36)		2.27 (2.18,2.35)		2.47 (2.37,2.56)		2.72 (2.62,2.83)	
	>0.720	2.30 (2.14,2.45)	0.697	2.31 (2.13,2.48)	0.684	2.51 (2.30,2.72)	0.716	2.77 (2.52,3.01)	0.744
∑MBP(i+n)	<=2.860	2.24 (2.12,2.37)		2.23 (2.11,2.35)		2.41 (2.26,2.55)		2.65 (2.47,2.83)	
	>2.860 & <=5.986	2.23 (2.10,2.36)	0.873	2.25 (2.13,2.37)	0.840	2.46 (2.32,2.60)	0.617	2.72 (2.57,2.88)	0.535
	>5.986	2.33 (2.17,2.49)	0.406	2.34 (2.20,2.49)	0.247	2.55 (2.40,2.70)	0.197	2.81 (2.64,2.97)	0.202
∑DEHP	<=7.700	2.28 (2.13,2.43)		2.33 (2.19,2.46)		2.56 (2.41,2.71)		2.84 (2.67,3.00)	
	>7.700 & <=11.320	2.25 (2.12,2.39)	0.766	2.27 (2.15,2.39)	0.550	2.48 (2.33,2.62)	0.431	2.74 (2.57,2.91)	0.406
	>11.320	2.26 (2.13,2.40)	0.852	2.23 (2.10,2.36)	0.327	2.39 (2.25,2.53)	0.101	2.61 (2.46,2.77)	0.056
∑DiNP	<=3.700	2.29 (2.14,2.44)		2.30 (2.17,2.43)		2.50 (2.35,2.65)		2.76 (2.59,2.93)	
	>3.700 & <=8.292	2.23 (2.09,2.38)	0.577	2.23 (2.10,2.37)	0.495	2.43 (2.27,2.58)	0.493	2.68 (2.50,2.86)	0.524
	>8.292	2.28 (2.15,2.41)	0.938	2.29 (2.17,2.41)	0.934	2.49 (2.36,2.62)	0.941	2.75 (2.61,2.90)	0.949
∑DEHPDiNP	<=9.529	2.27 (2.13,2.41)		2.27 (2.14,2.40)		2.46 (2.31,2.61)		2.71 (2.54,2.89)	
	>9.529 & <=12.850	2.30 (2.15,2.46)	0.739	2.33 (2.20,2.47)	0.498	2.56 (2.41,2.70)	0.367	2.83 (2.66,3.00)	0.337
	>12.850	2.23 (2.11,2.35)	0.678	2.22 (2.10,2.35)	0.602	2.41 (2.27,2.54)	0.592	2.65 (2.50,2.80)	0.612
∑low MWphth	<=5.550	2.28 (2.14,2.43)		2.28 (2.15,2.41)		2.47 (2.32,2.62)		2.72 (2.55,2.89)	
	>5.550 & <=12.300	2.25 (2.10,2.40)	0.710	2.24 (2.11,2.37)	0.675	2.43 (2.29,2.57)	0.693	2.68 (2.52,2.84)	0.725
	>12.300	2.28 (2.15,2.41)	0.936	2.30 (2.17,2.43)	0.832	2.52 (2.37,2.66)	0.647	2.79 (2.62,2.95)	0.573
∑highMWphth	<=9.770	2.27 (2.13,2.42)		2.28 (2.15,2.41)		2.48 (2.33,2.63)		2.74 (2.56,2.91)	
	>9.770 & <=13.420	2.27 (2.12,2.42)	0.988	2.29 (2.14,2.43)	0.947	2.49 (2.34,2.65)	0.892	2.76 (2.58,2.94)	0.870
	>13.420	2.26 (2.13,2.38)	0.873	2.26 (2.14,2.37)	0.784	2.45 (2.32,2.57)	0.735	2.70 (2.55,2.84)	0.729
∑allphth	<=19.780	2.30 (2.15,2.46)		2.28 (2.14,2.41)		2.44 (2.29,2.60)		2.68 (2.50,2.86)	
	>19.780 & <=30.900	2.26 (2.12,2.39)	0.672	2.28 (2.16,2.41)	0.948	2.50 (2.37,2.63)	0.585	2.77 (2.62,2.92)	0.440
	>30.900	2.25 (2.12,2.38)	0.592	2.26 (2.13,2.39)	0.853	2.46 (2.32,2.60)	0.856	2.73 (2.56,2.89)	0.711

Models were adjusted for age at measurement. * Indicates a significant overall effect of the maternal serum phthalate levels on the outcome. Participants who were not fasting for their blood test or who had triglycerides greater than 4.5 were removed.

Table S31. Age Adjusted Associations Between Categorized Phthalate Metabolite Levels and Uric Acid (mmol/L)

		14 year followup		20 year followup		22 year followup	
		Marginal Mean (95%CI)	p	Marginal Mean (95%CI)	p	Marginal Mean (95%CI)	p
MEP	<=1.750	0.33 (0.32,0.35)		0.35 (0.34,0.37)		0.39 (0.37,0.41)	
	>1.750 & <=5.714	0.32 (0.31,0.33)	0.309	0.35 (0.34,0.36)	0.652	0.38 (0.37,0.40)	0.845
	>5.714	0.31 (0.29,0.32)	0.039	0.34 (0.33,0.36)	0.408	0.38 (0.36,0.40)	0.728
MiBP	<=0.792	0.32 (0.30,0.33)		0.35 (0.34,0.37)		0.39 (0.38,0.41)	
	>0.792 & <=1.900	0.32 (0.30,0.33)	0.924	0.34 (0.32,0.35)	0.088	0.37 (0.35,0.39)	0.059
	>1.900	0.32 (0.31,0.33)	0.900	0.35 (0.34,0.36)	0.576	0.39 (0.37,0.40)	0.501
MnBP	<=1.999	0.32 (0.31,0.34)	*	0.35 (0.34,0.37)		0.39 (0.37,0.41)	
	>1.999 & <=4.099	0.33 (0.32,0.34)	0.328	0.35 (0.34,0.36)	0.797	0.38 (0.37,0.40)	0.582
	>4.099	0.30 (0.29,0.32)	0.100	0.34 (0.33,0.35)	0.171	0.38 (0.36,0.39)	0.286
MHBP	<=0.220	0.32 (0.31,0.33)		0.35 (0.34,0.36)		0.39 (0.37,0.40)	
	>0.220	0.32 (0.30,0.33)	0.825	0.34 (0.32,0.36)	0.536	0.38 (0.36,0.40)	0.526
MBzP	<=0.260	0.32 (0.31,0.33)		0.35 (0.34,0.36)		0.38 (0.37,0.40)	
	>0.260	0.31 (0.30,0.33)	0.385	0.34 (0.33,0.36)	0.696	0.38 (0.36,0.40)	0.839
MEHP	<=2.960	0.32 (0.31,0.34)		0.35 (0.33,0.36)		0.38 (0.37,0.40)	
	>2.960 & <=5.250	0.32 (0.31,0.33)	0.722	0.35 (0.33,0.36)	0.860	0.38 (0.37,0.40)	0.923
	>5.250	0.32 (0.30,0.33)	0.463	0.35 (0.33,0.36)	0.909	0.39 (0.37,0.40)	0.925
MECPP	<=0.785	0.32 (0.30,0.33)		0.35 (0.34,0.37)		0.39 (0.37,0.41)	
	>0.785 & <=1.241	0.32 (0.31,0.34)	0.803	0.35 (0.34,0.37)	0.885	0.39 (0.37,0.41)	0.928
	>1.241	0.32 (0.30,0.33)	0.714	0.34 (0.33,0.35)	0.274	0.38 (0.36,0.39)	0.264
MCMHP	<=1.153	0.33 (0.32,0.34)		0.35 (0.34,0.37)		0.39 (0.37,0.41)	
	>1.153 & <=1.810	0.32 (0.31,0.33)	0.364	0.35 (0.33,0.36)	0.557	0.38 (0.37,0.40)	0.694
	>1.810	0.31 (0.29,0.32)	0.040	0.34 (0.33,0.36)	0.191	0.38 (0.36,0.40)	0.376
MCPP	<=0.190	0.32 (0.31,0.33)		0.35 (0.34,0.36)		0.39 (0.38,0.40)	
	>0.190	0.32 (0.30,0.33)	0.610	0.34 (0.33,0.36)	0.253	0.38 (0.36,0.39)	0.253
MINP	<=2.363	0.33 (0.31,0.34)		0.34 (0.33,0.36)		0.38 (0.36,0.40)	
	>2.363 & <=5.491	0.31 (0.29,0.32)	0.079	0.35 (0.33,0.36)	0.828	0.39 (0.37,0.41)	0.431
	>5.491	0.32 (0.31,0.33)	0.484	0.35 (0.34,0.36)	0.618	0.39 (0.37,0.40)	0.437
MCiOP	<=0.130	0.32 (0.30,0.34)		0.35 (0.33,0.36)		0.38 (0.37,0.40)	
	>0.130	0.32 (0.31,0.33)	0.985	0.35 (0.34,0.36)	0.863	0.38 (0.37,0.40)	0.843
MiDP	<=0.720	0.32 (0.31,0.33)		0.34 (0.33,0.35)		0.38 (0.37,0.39)	
	>0.720	0.33 (0.31,0.35)	0.173	0.36 (0.34,0.39)	0.091	0.40 (0.38,0.43)	0.122
∑MBP(i+n)	<=2.860	0.33 (0.31,0.34)		0.36 (0.34,0.37)		0.40 (0.38,0.42)	
	>2.860 & <=5.986	0.32 (0.31,0.33)	0.449	0.34 (0.33,0.36)	0.061	0.37 (0.35,0.39)	0.063
	>5.986	0.31 (0.30,0.33)	0.116	0.34 (0.33,0.36)	0.105	0.38 (0.37,0.40)	0.165
∑DEHP	<=7.700	0.33 (0.31,0.35)		0.36 (0.34,0.37)		0.39 (0.37,0.41)	
	>7.700 & <=11.320	0.31 (0.30,0.33)	0.073	0.34 (0.33,0.36)	0.225	0.38 (0.36,0.40)	0.388
	>11.320	0.32 (0.30,0.33)	0.192	0.35 (0.33,0.36)	0.346	0.38 (0.37,0.40)	0.514
∑DiNP	<=3.700	0.33 (0.31,0.34)		0.34 (0.33,0.36)		0.38 (0.36,0.40)	
	>3.700 & <=8.292	0.31 (0.30,0.32)	0.132	0.35 (0.33,0.36)	0.737	0.39 (0.37,0.41)	0.397
	>8.292	0.32 (0.31,0.34)	0.754	0.35 (0.34,0.36)	0.535	0.39 (0.37,0.40)	0.423
∑DEHPDiNP	<=9.529	0.32 (0.31,0.34)		0.34 (0.33,0.36)		0.38 (0.36,0.40)	
	>9.529 & <=12.850	0.32 (0.30,0.33)	0.452	0.34 (0.33,0.36)	0.945	0.38 (0.36,0.40)	0.753
	>12.850	0.32 (0.31,0.33)	0.532	0.35 (0.34,0.37)	0.435	0.39 (0.37,0.41)	0.287
∑low MWphth	<=5.550	0.33 (0.32,0.34)		0.35 (0.33,0.37)		0.38 (0.37,0.40)	
	>5.550 & <=12.300	0.32 (0.30,0.33)	0.265	0.35 (0.34,0.36)	0.955	0.39 (0.37,0.40)	0.799
	>12.300	0.31 (0.30,0.33)	0.069	0.34 (0.33,0.36)	0.488	0.38 (0.36,0.40)	0.803
∑highMWphth	<=9.770	0.32 (0.31,0.34)		0.34 (0.33,0.36)		0.38 (0.36,0.40)	
	>9.770 & <=13.420	0.32 (0.30,0.33)	0.426	0.34 (0.33,0.36)	0.924	0.38 (0.36,0.40)	0.893
	>13.420	0.32 (0.30,0.33)	0.422	0.35 (0.34,0.37)	0.298	0.39 (0.38,0.41)	0.157
∑allphth	<=19.780	0.33 (0.32,0.35)		0.35 (0.33,0.36)		0.38 (0.36,0.40)	
	>19.780 & <=30.900	0.32 (0.30,0.33)	0.146	0.35 (0.34,0.37)	0.607	0.39 (0.38,0.41)	0.320
	>30.900	0.31 (0.29,0.32)	0.025	0.34 (0.33,0.36)	0.645	0.38 (0.36,0.40)	0.919

Models were adjusted for age at measurement. * Indicates a significant overall effect of the maternal serum phthalate levels on the outcome.

Table S32. Age Adjusted Associations Between Categorized Phthalate Metabolite Levels and High Sensitivity C-Reactive Protein (mg/L)

		14 year followup		17 year followup		20 year followup		22 year followup	
		Marginal Geometric Mean (95%CI)	p	Marginal Geometric Mean (95%CI)	p	Marginal Geometric Mean (95%CI)	p	Marginal Geometric Mean (95%CI)	p
MEP	<=1.750	0.68 (0.50,0.93)		0.57 (0.45,0.73)		0.78 (0.62,0.98)		0.91 (0.70,1.17)	
	>1.750 & <=5.714	0.56 (0.43,0.74)	0.353	0.49 (0.39,0.62)	0.322	0.70 (0.57,0.85)	0.436	0.83 (0.67,1.04)	0.617
	>5.714	0.52 (0.41,0.66)	0.159	0.45 (0.37,0.55)	0.099	0.63 (0.52,0.77)	0.157	0.75 (0.59,0.96)	0.300
MiBP	<=0.792	0.71 (0.53,0.95)	*	0.56 (0.45,0.71)		0.72 (0.57,0.91)		0.80 (0.60,1.06)	
	>0.792 & <=1.900	0.62 (0.46,0.84)	0.528	0.54 (0.43,0.67)	0.805	0.76 (0.62,0.94)	0.698	0.91 (0.70,1.18)	0.513
	>1.900	0.46 (0.36,0.58)	0.019	0.42 (0.34,0.52)	0.043	0.63 (0.52,0.77)	0.361	0.78 (0.64,0.95)	0.892
MnBP	<=1.999	0.58 (0.43,0.77)		0.59 (0.47,0.73)		0.73 (0.58,0.93)		0.98 (0.73,1.32)	
	>1.999 & <=4.099	0.64 (0.48,0.84)	0.623	0.56 (0.46,0.69)	0.781	0.61 (0.51,0.73)	0.218	0.74 (0.61,0.91)	0.120
	>4.099	0.48 (0.37,0.62)	0.310	0.49 (0.39,0.60)	0.191	0.61 (0.50,0.75)	0.236	0.82 (0.65,1.04)	0.356
MHBP	<=0.220	0.56 (0.46,0.69)		0.49 (0.41,0.58)		0.69 (0.59,0.80)		0.81 (0.69,0.96)	
	>0.220	0.64 (0.49,0.84)	0.430	0.54 (0.43,0.68)	0.386	0.75 (0.61,0.92)	0.487	0.87 (0.68,1.12)	0.637
MBzP	<=0.260	0.61 (0.49,0.75)		0.52 (0.44,0.62)		0.73 (0.62,0.85)		0.86 (0.72,1.02)	
	>0.260	0.53 (0.41,0.69)	0.383	0.46 (0.36,0.59)	0.320	0.65 (0.52,0.81)	0.372	0.77 (0.62,0.97)	0.491
MEHP	<=2.960	0.59 (0.45,0.78)		0.52 (0.42,0.65)		0.74 (0.60,0.92)		0.88 (0.69,1.12)	
	>2.960 & <=5.250	0.60 (0.47,0.78)	0.934	0.53 (0.43,0.66)	0.888	0.76 (0.62,0.93)	0.865	0.90 (0.73,1.11)	0.877
	>5.250	0.55 (0.41,0.73)	0.709	0.46 (0.36,0.59)	0.381	0.62 (0.50,0.77)	0.210	0.70 (0.54,0.92)	0.219
MECPP	<=0.785	0.55 (0.41,0.74)		0.50 (0.39,0.63)		0.73 (0.58,0.92)		0.90 (0.67,1.20)	
	>0.785 & <=1.241	0.62 (0.48,0.80)	0.512	0.52 (0.43,0.64)	0.702	0.71 (0.59,0.87)	0.880	0.83 (0.66,1.05)	0.686
	>1.241	0.58 (0.44,0.76)	0.784	0.49 (0.39,0.61)	0.925	0.67 (0.54,0.83)	0.567	0.78 (0.63,0.97)	0.449
MCMHP	<=1.153	0.57 (0.43,0.75)		0.58 (0.47,0.71)		0.72 (0.58,0.90)		0.95 (0.71,1.29)	
	>1.153 & <=1.810	0.66 (0.49,0.88)	0.471	0.60 (0.49,0.74)	0.795	0.67 (0.56,0.81)	0.633	0.83 (0.67,1.02)	0.426
	>1.810	0.48 (0.37,0.62)	0.368	0.48 (0.39,0.58)	0.162	0.57 (0.47,0.70)	0.121	0.74 (0.60,0.92)	0.170
MCPP	<=0.190	0.61 (0.49,0.75)		0.53 (0.45,0.64)		0.76 (0.64,0.90)		0.90 (0.75,1.09)	
	>0.190	0.55 (0.43,0.70)	0.490	0.46 (0.38,0.57)	0.233	0.64 (0.53,0.77)	0.135	0.74 (0.60,0.92)	0.159
MINP	<=2.363	0.43 (0.33,0.57)	*	0.41 (0.33,0.51)	*	0.63 (0.51,0.78)		0.79 (0.63,1.00)	
	>2.363 & <=5.491	0.70 (0.52,0.94)	0.015	0.61 (0.47,0.78)	0.007	0.86 (0.68,1.08)	0.037	1.02 (0.77,1.34)	0.178
	>5.491	0.63 (0.50,0.81)	0.033	0.50 (0.41,0.61)	0.124	0.64 (0.53,0.77)	0.918	0.70 (0.57,0.87)	0.446
MCiOP	<=0.130	0.56 (0.41,0.77)		0.51 (0.41,0.64)		0.74 (0.59,0.93)		0.91 (0.69,1.21)	
	>0.130	0.59 (0.49,0.71)	0.792	0.50 (0.42,0.59)	0.880	0.68 (0.58,0.79)	0.499	0.80 (0.68,0.94)	0.405
MiDP	<=0.720	0.58 (0.48,0.69)		0.50 (0.43,0.59)		0.70 (0.61,0.81)		0.83 (0.71,0.98)	
	>0.720	0.60 (0.39,0.91)	0.880	0.51 (0.37,0.70)	0.942	0.70 (0.52,0.93)	0.948	0.81 (0.59,1.11)	0.885
∑MBP(i+n)	<=2.860	0.66 (0.49,0.89)		0.56 (0.44,0.72)		0.78 (0.61,1.00)		0.92 (0.68,1.24)	
	>2.860 & <=5.986	0.58 (0.44,0.76)	0.503	0.49 (0.39,0.60)	0.310	0.66 (0.55,0.80)	0.263	0.77 (0.63,0.95)	0.331
	>5.986	0.52 (0.40,0.67)	0.211	0.46 (0.37,0.57)	0.165	0.67 (0.54,0.82)	0.295	0.81 (0.64,1.01)	0.495
∑DEHP	<=7.700	0.60 (0.45,0.79)		0.54 (0.44,0.65)		0.77 (0.63,0.95)		0.93 (0.73,1.17)	
	>7.700 & <=11.320	0.67 (0.52,0.87)	0.514	0.55 (0.43,0.70)	0.855	0.72 (0.58,0.89)	0.602	0.80 (0.62,1.04)	0.419
	>11.320	0.49 (0.37,0.65)	0.281	0.44 (0.35,0.55)	0.145	0.63 (0.52,0.78)	0.141	0.76 (0.61,0.95)	0.237
∑DiNP	<=3.700	0.43 (0.33,0.57)	*	0.41 (0.34,0.51)		0.65 (0.52,0.80)		0.81 (0.64,1.03)	
	>3.700 & <=8.292	0.66 (0.50,0.88)	0.028	0.58 (0.45,0.73)	0.021	0.82 (0.65,1.02)	0.107	0.96 (0.74,1.25)	0.360
	>8.292	0.65 (0.51,0.84)	0.024	0.51 (0.42,0.63)	0.096	0.66 (0.54,0.80)	0.883	0.72 (0.58,0.89)	0.451
∑DEHPDiNP	<=9.529	0.51 (0.39,0.68)		0.53 (0.43,0.65)		0.67 (0.54,0.82)		0.89 (0.69,1.14)	
	>9.529 & <=12.850	0.67 (0.51,0.86)	0.170	0.62 (0.50,0.77)	0.269	0.70 (0.56,0.88)	0.733	0.86 (0.67,1.12)	0.889
	>12.850	0.51 (0.39,0.68)	0.992	0.50 (0.41,0.61)	0.668	0.59 (0.49,0.71)	0.359	0.75 (0.60,0.93)	0.301
∑low MWphth	<=5.550	0.66 (0.49,0.90)		0.55 (0.43,0.70)		0.73 (0.57,0.93)		0.84 (0.64,1.11)	
	>5.550 & <=12.300	0.55 (0.42,0.72)	0.331	0.50 (0.41,0.62)	0.525	0.74 (0.60,0.90)	0.954	0.91 (0.72,1.15)	0.660
	>12.300	0.55 (0.43,0.70)	0.323	0.47 (0.37,0.58)	0.265	0.64 (0.52,0.78)	0.356	0.75 (0.60,0.93)	0.516
∑highMWphth	<=9.770	0.55 (0.41,0.73)		0.50 (0.41,0.62)		0.75 (0.60,0.93)		0.91 (0.71,1.17)	
	>9.770 & <=13.420	0.68 (0.52,0.88)	0.263	0.56 (0.44,0.71)	0.453	0.75 (0.59,0.95)	0.993	0.84 (0.65,1.10)	0.674
	>13.420	0.53 (0.40,0.70)	0.885	0.46 (0.37,0.57)	0.494	0.64 (0.53,0.77)	0.220	0.74 (0.59,0.91)	0.191
∑allphth	<=19.780	0.72 (0.54,0.96)		0.59 (0.47,0.75)		0.77 (0.60,0.98)		0.87 (0.64,1.17)	
	>19.780 & <=30.900	0.50 (0.37,0.67)	0.069	0.47 (0.38,0.59)	0.138	0.71 (0.59,0.87)	0.621	0.90 (0.73,1.10)	0.873
	>30.900	0.54 (0.43,0.69)	0.132	0.46 (0.37,0.58)	0.093	0.63 (0.52,0.77)	0.193	0.73 (0.59,0.92)	0.375

Models were adjusted for age at measurement. * Indicates a significant overall effect of the maternal serum phthalate levels on the outcome. Participants with hsCRP values greater than 10 were removed. Values below the limit of detection (0.15) were set as LOD/ √2

Table S33. Age Adjusted Associations Between Categorized Phthalate Metabolite Levels and Insulin (mU/L)

		14 year followup		17 year followup		20 year followup		22 year followup	
		Marginal Geometric Mean (95%CI)	p	Marginal Geometric Mean (95%CI)	p	Marginal Geometric Mean (95%CI)	p	Marginal Geometric Mean (95%CI)	p
MEP	<=1.750	9.62 (8.55,10.82)		6.50 (5.76,7.34)		3.47 (3.00,4.02)		5.81 (4.74,7.12)	
	>1.750 & <=5.714	10.55 (9.05,12.29)	0.353	6.51 (5.65,7.49)	0.990	3.17 (2.77,3.62)	0.324	4.96 (4.23,5.82)	0.190
	>5.714	9.83 (8.63,11.20)	0.810	6.43 (5.59,7.41)	0.889	3.32 (2.81,3.92)	0.676	5.43 (4.37,6.73)	0.624
MiBP	<=0.792	10.22 (9.09,11.48)		6.58 (5.79,7.48)		3.37 (2.85,3.98)		5.49 (4.38,6.89)	
	>0.792 & <=1.900	10.07 (8.46,11.98)	0.889	6.75 (5.78,7.87)	0.781	3.60 (3.06,4.23)	0.552	6.04 (4.99,7.30)	0.501
	>1.900	9.78 (8.70,10.99)	0.608	6.14 (5.42,6.95)	0.343	3.07 (2.72,3.46)	0.335	4.90 (4.18,5.75)	0.382
MnBP	<=1.999	9.85 (8.31,11.67)		6.39 (5.48,7.44)		3.28 (2.77,3.88)		5.33 (4.26,6.67)	
	>1.999 & <=4.099	10.23 (9.13,11.46)	0.717	6.70 (5.94,7.56)	0.570	3.47 (3.03,3.98)	0.581	5.69 (4.74,6.83)	0.634
	>4.099	9.96 (8.79,11.28)	0.915	6.35 (5.58,7.23)	0.942	3.20 (2.77,3.69)	0.817	5.14 (4.29,6.16)	0.782
MHBP	<=0.220	9.99 (9.11,10.96)		6.59 (5.93,7.33)		3.43 (3.07,3.83)		5.66 (4.95,6.46)	
	>0.220	10.12 (8.72,11.74)	0.892	6.20 (5.36,7.16)	0.405	2.99 (2.53,3.53)	0.153	4.67 (3.68,5.91)	0.128
MBzP	<=0.260	9.85 (8.92,10.87)		6.48 (5.82,7.21)		3.36 (3.01,3.76)		5.54 (4.87,6.32)	
	>0.260	10.39 (9.11,11.84)	0.524	6.50 (5.67,7.45)	0.972	3.20 (2.74,3.74)	0.583	5.08 (4.07,6.35)	0.464
MEHP	<=2.960	10.87 (9.35,12.64)		6.65 (5.82,7.61)		3.24 (2.77,3.78)		5.08 (4.26,6.05)	
	>2.960 & <=5.250	9.87 (8.66,11.26)	0.343	6.68 (5.83,7.66)	0.955	3.61 (3.07,4.24)	0.298	6.09 (4.90,7.57)	0.174
	>5.250	9.37 (8.29,10.60)	0.132	6.06 (5.31,6.92)	0.248	3.13 (2.75,3.56)	0.712	5.10 (4.25,6.13)	0.962
MECPP	<=0.785	9.99 (8.73,11.43)	*	6.63 (5.80,7.59)		3.45 (2.97,4.02)		5.72 (4.61,7.10)	
	>0.785 & <=1.241	11.42 (9.84,13.26)	0.185	7.06 (6.12,8.14)	0.465	3.42 (2.91,4.01)	0.918	5.37 (4.44,6.50)	0.644
	>1.241	8.96 (7.94,10.12)	0.241	5.96 (5.25,6.76)	0.164	3.10 (2.70,3.57)	0.285	5.14 (4.30,6.16)	0.415
MCMHP	<=1.153	10.89 (9.42,12.60)		6.86 (5.92,7.95)		3.40 (2.89,4.01)		5.39 (4.38,6.64)	
	>1.153 & <=1.810	10.38 (9.02,11.94)	0.635	6.58 (5.76,7.52)	0.628	3.29 (2.86,3.78)	0.739	5.24 (4.42,6.21)	0.824
	>1.810	9.01 (8.00,10.15)	0.049	6.10 (5.39,6.89)	0.152	3.26 (2.82,3.76)	0.673	5.44 (4.48,6.60)	0.951
MCPP	<=0.190	10.47 (9.47,11.58)		6.72 (6.01,7.50)		3.41 (3.01,3.86)		5.49 (4.7,6.41)	
	>0.190	9.41 (8.30,10.66)	0.191	6.17 (5.43,7.01)	0.207	3.20 (2.82,3.64)	0.454	5.24 (4.4,6.25)	0.666
MINP	<=2.363	10.10 (8.69,11.75)		6.44 (5.56,7.45)		3.24 (2.75,3.82)		5.22 (4.29,6.35)	
	>2.363 & <=5.491	10.40 (9.04,11.96)	0.786	6.68 (5.87,7.60)	0.664	3.39 (2.96,3.89)	0.652	5.49 (4.59,6.55)	0.690
	>5.491	9.61 (8.55,10.81)	0.608	6.33 (5.56,7.22)	0.847	3.30 (2.85,3.83)	0.863	5.45 (4.42,6.71)	0.754
MCiOP	<=0.130	10.94 (9.35,12.79)		6.82 (5.91,7.88)		3.38 (2.91,3.93)		5.35 (4.48,6.38)	
	>0.130	9.67 (8.85,10.56)	0.175	6.32 (5.68,7.04)	0.305	3.29 (2.94,3.67)	0.745	5.39 (4.63,6.26)	0.948
MiDP	<=0.720	9.95 (9.15,10.82)		6.46 (5.82,7.16)		3.32 (2.99,3.69)		5.41 (4.71,6.21)	
	>0.720	10.37 (8.35,12.88)	0.726	6.56 (5.50,7.83)	0.862	3.29 (2.73,3.96)	0.919	5.26 (4.14,6.68)	0.824
∑MBP(i+n)	<=2.860	10.17 (8.86,11.67)		6.60 (5.71,7.62)		3.40 (2.87,4.01)		5.54 (4.47,6.87)	
	>2.860 & <=5.986	9.99 (8.65,11.53)	0.862	6.63 (5.83,7.52)	0.958	3.49 (3.02,4.03)	0.795	5.79 (4.76,7.04)	0.750
	>5.986	9.90 (8.73,11.22)	0.777	6.20 (5.44,7.07)	0.453	3.08 (2.69,3.53)	0.345	4.91 (4.12,5.84)	0.350
∑DEHP	<=7.700	10.96 (9.34,12.86)		7.00 (6.09,8.04)		3.54 (2.99,4.19)		5.71 (4.69,6.95)	
	>7.700 & <=11.320	9.78 (8.63,11.09)	0.267	6.15 (5.38,7.01)	0.123	3.05 (2.66,3.51)	0.155	4.87 (4.01,5.92)	0.229
	>11.320	9.43 (8.36,10.65)	0.139	6.34 (5.56,7.24)	0.243	3.38 (2.94,3.88)	0.660	5.67 (4.68,6.86)	0.956
∑DiNP	<=3.700	9.83 (8.45,11.44)		6.23 (5.42,7.16)		3.13 (2.69,3.64)		5.03 (4.18,6.04)	
	>3.700 & <=8.292	10.55 (9.22,12.07)	0.497	6.83 (5.97,7.81)	0.265	3.51 (3.02,4.07)	0.247	5.73 (4.71,6.96)	0.305
	>8.292	9.73 (8.61,10.98)	0.915	6.35 (5.57,7.25)	0.806	3.29 (2.85,3.81)	0.611	5.41 (4.42,6.61)	0.570
∑DEHPDiNP	<=9.529	10.53 (8.97,12.36)		6.57 (5.71,7.56)		3.22 (2.74,3.79)		5.14 (4.21,6.29)	
	>9.529 & <=12.850	10.60 (9.31,12.07)	0.953	6.77 (5.91,7.75)	0.724	3.41 (2.91,3.99)	0.597	5.53 (4.57,6.69)	0.586
	>12.850	9.14 (8.16,10.24)	0.154	6.16 (5.41,7.02)	0.437	3.28 (2.87,3.74)	0.869	5.53 (4.58,6.69)	0.568
∑low MWphth	<=5.550	10.22 (8.97,11.64)		6.53 (5.72,7.45)		3.29 (2.82,3.84)		5.30 (4.29,6.55)	
	>5.550 & <=12.300	9.44 (8.12,10.98)	0.439	6.31 (5.48,7.25)	0.674	3.33 (2.87,3.85)	0.916	5.53 (4.61,6.64)	0.741
	>12.300	10.36 (9.14,11.75)	0.880	6.60 (5.78,7.55)	0.885	3.32 (2.87,3.85)	0.932	5.34 (4.43,6.43)	0.958
∑highMWphth	<=9.770	10.72 (9.15,12.56)		6.64 (5.78,7.62)		3.22 (2.74,3.78)		5.10 (4.18,6.22)	
	>9.770 & <=13.420	10.36 (9.13,11.75)	0.740	6.73 (5.84,7.77)	0.867	3.44 (2.92,4.05)	0.543	5.64 (4.62,6.88)	0.460
	>13.420	9.14 (8.11,10.28)	0.108	6.16 (5.43,6.98)	0.351	3.26 (2.87,3.71)	0.902	5.49 (4.57,6.60)	0.559
∑allphth	<=19.780	10.59 (9.06,12.37)		6.87 (5.94,7.95)		3.53 (2.96,4.21)		5.74 (4.54,7.25)	
	>19.780 & <=30.900	9.55 (8.48,10.76)	0.305	6.23 (5.52,7.04)	0.234	3.22 (2.83,3.67)	0.382	5.25 (4.41,6.26)	0.522
	>30.900	9.99 (8.74,11.42)	0.580	6.40 (5.57,7.36)	0.425	3.25 (2.81,3.76)	0.448	5.23 (4.37,6.25)	0.510

Models were adjusted for age at measurement. * Indicates a significant overall effect of the maternal serum phthalate levels on the outcome. Diabetics (participants with glucose greater than 7) were removed. Values below the limit of detection (2.0) were set as LOD/ √2

Table S34. Age Adjusted Associations Between Categorized Phthalate Metabolite Levels and Glucose (mmol/L)

		14 year followup		17 year followup		20 year followup		22 year followup	
		Marginal Mean (95%CI)	p	Marginal Mean (95%CI)	p	Marginal Mean (95%CI)	p	Marginal Mean (95%CI)	p
MEP	<=1.750	4.84 (4.76, 4.92)		4.84 (4.77, 4.91)		5.05 (4.98, 5.13)		5.10 (5.02, 5.19)	
	>1.750 & <=5.714	4.91 (4.83, 4.99)	0.212	4.90 (4.83, 4.97)	0.185	5.10 (5.03, 5.17)	0.341	5.14 (5.05, 5.23)	0.540
	>5.714	4.80 (4.72, 4.88)	0.502	4.80 (4.72, 4.87)	0.356	5.01 (4.93, 5.08)	0.369	5.06 (4.97, 5.15)	0.451
MiBP	<=0.792	4.90 (4.82, 4.99)		4.88 (4.81, 4.95)		5.07 (5.00, 5.15)		5.11 (5.02, 5.20)	
	>0.792 & <=1.900	4.87 (4.78, 4.95)	0.544	4.85 (4.78, 4.93)	0.531	5.05 (4.97, 5.13)	0.677	5.09 (4.99, 5.19)	0.802
	>1.900	4.79 (4.71, 4.87)	0.038	4.80 (4.73, 4.87)	0.076	5.04 (4.97, 5.11)	0.421	5.10 (5.02, 5.18)	0.858
MnBP	<=1.999	4.91 (4.82, 4.99)		4.88 (4.81, 4.95)		5.08 (5.00, 5.15)		5.11 (5.01, 5.21)	
	>1.999 & <=4.099	4.84 (4.76, 4.92)	0.252	4.84 (4.77, 4.91)	0.337	5.06 (4.98, 5.13)	0.695	5.11 (5.02, 5.19)	0.964
	>4.099	4.81 (4.73, 4.89)	0.096	4.81 (4.74, 4.88)	0.115	5.03 (4.96, 5.10)	0.366	5.08 (5.00, 5.17)	0.670
MHBP	<=0.220	4.82 (4.76, 4.87)		4.91 (4.87, 4.95)		5.01 (4.97, 5.06)		5.08 (5.02, 5.14)	
	>0.220	4.85 (4.76, 4.94)	0.566	4.94 (4.86, 5.01)	0.594	5.02 (4.95, 5.10)	0.771	5.09 (4.99, 5.19)	0.904
MBzP	<=0.260	4.87 (4.81, 4.93)		4.85 (4.79, 4.90)		5.04 (4.99, 5.10)		5.07 (5.00, 5.13)	
	>0.260	4.81 (4.73, 4.90)	0.234	4.84 (4.76, 4.91)	0.878	5.09 (5.02, 5.17)	0.243	5.16 (5.07, 5.25)	0.090
MEHP	<=2.960	4.85 (4.77, 4.93)		4.85 (4.78, 4.92)		5.06 (4.98, 5.13)		5.10 (5.02, 5.18)	
	>2.960 & <=5.250	4.81 (4.73, 4.89)	0.444	4.82 (4.75, 4.89)	0.535	5.04 (4.97, 5.12)	0.814	5.10 (5.01, 5.19)	0.999
	>5.250	4.89 (4.81, 4.97)	0.495	4.87 (4.80, 4.94)	0.576	5.07 (4.99, 5.14)	0.832	5.10 (5.01, 5.19)	0.998
MECPP	<=0.785	4.89 (4.8, 4.97)		4.88 (4.81, 4.95)		5.09 (5.01, 5.16)		5.13 (5.04, 5.23)	
	>0.785 & <=1.241	4.86 (4.78, 4.95)	0.703	4.85 (4.77, 4.92)	0.469	5.04 (4.97, 5.12)	0.383	5.08 (4.99, 5.17)	0.413
	>1.241	4.81 (4.73, 4.89)	0.183	4.82 (4.75, 4.89)	0.150	5.04 (4.97, 5.11)	0.290	5.09 (5.01, 5.17)	0.482
MCMHP	<=1.153	4.88 (4.8, 4.96)		4.87 (4.80, 4.94)		5.07 (4.99, 5.14)		5.11 (5.02, 5.20)	
	>1.153 & <=1.810	4.83 (4.75, 4.92)	0.395	4.83 (4.76, 4.91)	0.450	5.05 (4.97, 5.12)	0.708	5.10 (5.01, 5.19)	0.902
	>1.810	4.84 (4.76, 4.92)	0.450	4.84 (4.77, 4.91)	0.486	5.05 (4.98, 5.12)	0.702	5.10 (5.01, 5.18)	0.870
MCPP	<=0.190	4.85 (4.78, 4.91)		4.84 (4.78, 4.90)		5.05 (5.00, 5.11)		5.10 (5.03, 5.17)	
	>0.190	4.86 (4.78, 4.93)	0.793	4.85 (4.78, 4.91)	0.870	5.05 (4.99, 5.12)	0.990	5.10 (5.02, 5.18)	0.918
MINP	<=2.363	4.82 (4.74, 4.9)		4.81 (4.73, 4.88)		5.01 (4.94, 5.09)		5.06 (4.97, 5.15)	
	>2.363 & <=5.491	4.90 (4.81, 4.98)	0.183	4.87 (4.80, 4.94)	0.165	5.06 (4.99, 5.13)	0.326	5.10 (5.01, 5.19)	0.533
	>5.491	4.84 (4.76, 4.92)	0.683	4.85 (4.78, 4.92)	0.300	5.08 (5.01, 5.15)	0.155	5.15 (5.06, 5.23)	0.157
MCiOP	<=0.130	4.82 (4.73, 4.91)		4.82 (4.75, 4.90)		5.04 (4.96, 5.12)		5.09 (5.00, 5.19)	
	>0.130	4.87 (4.81, 4.92)	0.350	4.86 (4.80, 4.91)	0.386	5.06 (5.01, 5.12)	0.637	5.10 (5.04, 5.17)	0.845
MiDP	<=0.720	4.86 (4.81, 4.92)		4.85 (4.80, 4.91)		5.06 (5.01, 5.11)		5.11 (5.05, 5.16)	
	>0.720	4.80 (4.68, 4.91)	0.294	4.80 (4.70, 4.90)	0.290	5.02 (4.92, 5.12)	0.473	5.08 (4.96, 5.20)	0.664
∑MBP(i+n)	<=2.860	4.90 (4.82, 4.99)		4.88 (4.81, 4.95)		5.07 (5.00, 5.15)		5.11 (5.01, 5.20)	
	>2.860 & <=5.986	4.83 (4.75, 4.91)	0.218	4.84 (4.76, 4.91)	0.320	5.05 (4.98, 5.13)	0.722	5.11 (5.02, 5.19)	0.984
	>5.986	4.82 (4.74, 4.9)	0.145	4.82 (4.75, 4.89)	0.185	5.04 (4.96, 5.11)	0.482	5.09 (5.01, 5.17)	0.783
∑DEHP	<=7.700	4.84 (4.75, 4.92)		4.93 (4.86, 4.99)		5.02 (4.95, 5.09)		5.08 (5.00, 5.17)	
	>7.700 & <=11.320	4.80 (4.72, 4.88)	0.497	4.89 (4.83, 4.95)	0.460	4.99 (4.92, 5.06)	0.581	5.06 (4.98, 5.14)	0.710
	>11.320	4.84 (4.76, 4.92)	0.893	4.94 (4.88, 5.00)	0.769	5.04 (4.97, 5.10)	0.705	5.11 (5.02, 5.19)	0.715
∑DiNP	<=3.700	4.79 (4.70, 4.87)		4.88 (4.81, 4.94)		4.97 (4.90, 5.04)		5.04 (4.95, 5.12)	
	>3.700 & <=8.292	4.87 (4.79, 4.96)	0.130	4.95 (4.89, 5.01)	0.104	5.03 (4.96, 5.10)	0.233	5.09 (5.00, 5.17)	0.431
	>8.292	4.81 (4.74, 4.89)	0.614	4.93 (4.87, 4.99)	0.272	5.04 (4.97, 5.11)	0.155	5.12 (5.04, 5.21)	0.166
∑DEHPDiNP	<=9.529	4.83 (4.75, 4.91)		4.82 (4.74, 4.89)		5.02 (4.94, 5.10)		5.07 (4.98, 5.16)	
	>9.529 & <=12.850	4.92 (4.83, 5.00)	0.124	4.88 (4.80, 4.95)	0.191	5.05 (4.97, 5.12)	0.562	5.08 (4.99, 5.17)	0.912
	>12.850	4.82 (4.74, 4.90)	0.831	4.84 (4.77, 4.91)	0.582	5.08 (5.01, 5.15)	0.203	5.16 (5.07, 5.25)	0.145
∑low MWphth	<=5.550	4.85 (4.77, 4.93)		4.93 (4.87, 4.99)		5.01 (4.94, 5.08)		5.07 (4.98, 5.16)	
	>5.550 & <=12.300	4.83 (4.74, 4.91)	0.698	4.91 (4.85, 4.97)	0.635	4.99 (4.92, 5.06)	0.686	5.05 (4.97, 5.14)	0.759
	>12.300	4.80 (4.73, 4.88)	0.417	4.92 (4.86, 4.98)	0.795	5.04 (4.97, 5.10)	0.628	5.12 (5.04, 5.20)	0.425
∑highMWphth	<=9.770	4.84 (4.75, 4.92)		4.83 (4.76, 4.9)		5.04 (4.96, 5.11)		5.09 (5.00, 5.18)	
	>9.770 & <=13.420	4.91 (4.83, 4.99)	0.209	4.87 (4.80, 4.94)	0.368	5.05 (4.97, 5.12)	0.864	5.07 (4.98, 5.16)	0.814
	>13.420	4.81 (4.73, 4.89)	0.655	4.83 (4.77, 4.9)	0.944	5.07 (5.00, 5.14)	0.505	5.14 (5.05, 5.23)	0.380
∑allphth	<=19.780	4.87 (4.79, 4.95)		4.87 (4.80, 4.94)		5.09 (5.01, 5.17)		5.14 (5.05, 5.23)	
	>19.780 & <=30.900	4.86 (4.78, 4.94)	0.866	4.84 (4.77, 4.90)	0.437	5.03 (4.96, 5.10)	0.221	5.06 (4.97, 5.14)	0.203
	>30.900	4.83 (4.75, 4.91)	0.464	4.83 (4.76, 4.91)	0.419	5.06 (4.99, 5.13)	0.536	5.11 (5.03, 5.20)	0.666

Models were adjusted for age at measurement. Diabetics (participants with glucose greater than 7), and participants who were not fasting for their blood test were removed.

Table S35. Age Adjusted Associations Between Categorized Phthalate Metabolite Levels and Homeostatic Model Assessment (HOMA - fasting insulin (microunits per millilitre) × fasting glucose (millimoles per litre)/22.5)

		14 year followup		17 year followup		20 year followup		22 year followup	
		Marginal Geometric Mean (95%CI)	p	Marginal Geometric Mean (95%CI)	p	Marginal Geometric Mean (95%CI)	p	Marginal Geometric Mean (95%CI)	p
MEP	<=1.750	2.07 (1.83, 2.35)		1.37 (1.21, 1.56)		0.78 (0.67, 0.91)		1.32 (1.07, 1.63)	
	>1.750 & <=5.714	2.30 (1.94, 2.73)	0.342	1.39 (1.19, 1.61)	0.907	0.71 (0.62, 0.82)	0.380	1.13 (0.96, 1.33)	0.221
	>5.714	2.13 (1.86, 2.43)	0.791	1.35 (1.16, 1.56)	0.813	0.73 (0.62, 0.86)	0.549	1.20 (0.97, 1.48)	0.489
MiBP	<=0.792	2.25 (1.98, 2.56)		1.40 (1.22, 1.60)		0.75 (0.63, 0.88)		1.22 (0.97, 1.53)	
	>0.792 & <=1.900	2.17 (1.80, 2.62)	0.737	1.42 (1.20, 1.68)	0.864	0.80 (0.68, 0.95)	0.519	1.36 (1.12, 1.66)	0.426
	>1.900	2.08 (1.84, 2.36)	0.389	1.29 (1.13, 1.46)	0.276	0.68 (0.6, 0.77)	0.383	1.11 (0.94, 1.31)	0.492
MnBP	<=1.999	2.14 (1.77, 2.57)		1.36 (1.15, 1.60)		0.74 (0.62, 0.88)		1.22 (0.97, 1.53)	
	>1.999 & <=4.099	2.23 (1.98, 2.52)	0.696	1.42 (1.25, 1.62)	0.593	0.78 (0.67, 0.90)	0.632	1.29 (1.06, 1.56)	0.697
	>4.099	2.13 (1.86, 2.44)	0.990	1.32 (1.15, 1.51)	0.784	0.70 (0.61, 0.81)	0.644	1.14 (0.95, 1.36)	0.622
MHBP	<=0.220	2.14 (1.94, 2.37)		1.38 (1.23, 1.54)		0.76 (0.68, 0.85)		1.27 (1.11, 1.45)	
	>0.220	2.26 (1.93, 2.66)	0.564	1.34 (1.15, 1.56)	0.721	0.68 (0.57, 0.81)	0.256	1.06 (0.83, 1.36)	0.181
MBzP	<=0.260	2.13 (1.91, 2.37)		1.36 (1.21, 1.53)		0.74 (0.67, 0.83)		1.24 (1.09, 1.41)	
	>0.260	2.25 (1.96, 2.59)	0.523	1.38 (1.20, 1.59)	0.843	0.72 (0.61, 0.85)	0.756	1.16 (0.92, 1.47)	0.624
MEHP	<=2.960	2.37 (2.01, 2.79)		1.41 (1.22, 1.63)		0.72 (0.62, 0.85)		1.15 (0.96, 1.37)	
	>2.960 & <=5.250	2.15 (1.86, 2.47)	0.375	1.41 (1.22, 1.63)	0.981	0.80 (0.68, 0.94)	0.371	1.35 (1.09, 1.69)	0.224
	>5.250	2.01 (1.76, 2.30)	0.126	1.28 (1.11, 1.47)	0.251	0.70 (0.61, 0.80)	0.750	1.17 (0.96, 1.41)	0.896
MECPP	<=0.785	2.15 (1.86, 2.49)	*	1.40 (1.22, 1.62)		0.78 (0.66, 0.91)		1.31 (1.05, 1.63)	
	>0.785 & <=1.241	2.50 (2.14, 2.94)	0.166	1.50 (1.29, 1.75)	0.459	0.76 (0.65, 0.90)	0.873	1.21 (0.99, 1.47)	0.577
	>1.241	1.92 (1.68, 2.19)	0.249	1.24 (1.09, 1.42)	0.137	0.68 (0.59, 0.78)	0.203	1.14 (0.95, 1.37)	0.304
MCMHP	<=1.153	2.37 (2.03, 2.77)		1.46 (1.25, 1.70)		0.76 (0.64, 0.90)		1.22 (0.99, 1.51)	
	>1.153 & <=1.810	2.27 (1.95, 2.64)	0.676	1.40 (1.21, 1.61)	0.661	0.74 (0.64, 0.85)	0.752	1.19 (0.99, 1.42)	0.827
	>1.810	1.92 (1.68, 2.19)	0.042	1.27 (1.11, 1.45)	0.116	0.72 (0.62, 0.83)	0.566	1.21 (1.00, 1.47)	0.956
MCPP	<=0.190	2.26 (2.03, 2.52)		1.42 (1.26, 1.60)		0.76 (0.67, 0.87)		1.25 (1.06, 1.47)	
	>0.190	2.04 (1.78, 2.33)	0.244	1.30 (1.13, 1.48)	0.206	0.71 (0.62, 0.80)	0.357	1.16 (0.98, 1.39)	0.520
MINP	<=2.363	2.15 (1.83, 2.54)		1.34 (1.15, 1.57)		0.72 (0.61, 0.85)		1.17 (0.96, 1.43)	
	>2.363 & <=5.491	2.26 (1.94, 2.64)	0.669	1.42 (1.23, 1.63)	0.534	0.76 (0.66, 0.88)	0.534	1.25 (1.04, 1.50)	0.595
	>5.491	2.10 (1.85, 2.38)	0.815	1.34 (1.17, 1.53)	0.983	0.73 (0.63, 0.85)	0.845	1.21 (0.98, 1.50)	0.786
MCiOP	<=0.130	2.31 (1.94, 2.75)		1.43 (1.22, 1.66)		0.75 (0.65, 0.88)		1.22 (1.02, 1.46)	
	>0.130	2.11 (1.92, 2.32)	0.372	1.34 (1.20, 1.50)	0.447	0.73 (0.65, 0.82)	0.718	1.21 (1.04, 1.41)	0.920
MiDP	<=0.720	2.16 (1.97, 2.37)		1.37 (1.22, 1.52)		0.74 (0.67, 0.82)		1.22 (1.06, 1.40)	
	>0.720	2.20 (1.75, 2.78)	0.877	1.37 (1.14, 1.65)	0.959	0.73 (0.60, 0.89)	0.923	1.19 (0.93, 1.53)	0.876
∑MBP(i+n)	<=2.860	2.21 (1.90, 2.57)		1.40 (1.20, 1.63)		0.76 (0.64, 0.91)		1.27 (1.01, 1.58)	
	>2.860 & <=5.986	2.17 (1.86, 2.53)	0.881	1.39 (1.22, 1.59)	0.948	0.77 (0.67, 0.89)	0.961	1.28 (1.05, 1.56)	0.924
	>5.986	2.13 (1.86, 2.43)	0.733	1.30 (1.14, 1.50)	0.422	0.69 (0.60, 0.79)	0.321	1.11 (0.93, 1.33)	0.333
∑DEHP	<=7.700	2.39 (2.01, 2.85)		1.49 (1.28, 1.73)		0.79 (0.66, 0.94)		1.29 (1.06, 1.58)	
	>7.700 & <=11.320	2.12 (1.85, 2.42)	0.263	1.30 (1.13, 1.49)	0.125	0.68 (0.59, 0.79)	0.155	1.10 (0.89, 1.34)	0.232
	>11.320	2.02 (1.77, 2.31)	0.132	1.33 (1.15, 1.53)	0.220	0.75 (0.65, 0.86)	0.610	1.27 (1.05, 1.54)	0.924
∑DiNP	<=3.700	2.07 (1.75, 2.44)		1.29 (1.11, 1.50)		0.69 (0.59, 0.81)		1.13 (0.94, 1.36)	
	>3.700 & <=8.292	2.32 (2.01, 2.69)	0.304	1.46 (1.26, 1.69)	0.158	0.79 (0.68, 0.93)	0.187	1.30 (1.07, 1.60)	0.275
	>8.292	2.13 (1.87, 2.42)	0.792	1.34 (1.17, 1.54)	0.636	0.73 (0.63, 0.85)	0.600	1.20 (0.98, 1.48)	0.629
∑DEHPDiNP	<=9.529	2.25 (1.89, 2.68)		1.37 (1.18, 1.60)		0.71 (0.60, 0.84)		1.16 (0.94, 1.42)	
	>9.529 & <=12.850	2.38 (2.07, 2.73)	0.616	1.46 (1.27, 1.69)	0.489	0.77 (0.65, 0.90)	0.515	1.25 (1.03, 1.52)	0.584
	>12.850	1.93 (1.71, 2.19)	0.166	1.28 (1.12, 1.47)	0.439	0.73 (0.64, 0.83)	0.875	1.25 (1.03, 1.51)	0.564
∑low MWphth	<=5.550	2.20 (1.91, 2.55)		1.38 (1.20, 1.59)		0.74 (0.63, 0.87)		1.20 (0.96, 1.50)	
	>5.550 & <=12.300	2.07 (1.75, 2.44)	0.570	1.34 (1.15, 1.56)	0.729	0.74 (0.63, 0.86)	0.981	1.24 (1.02, 1.49)	0.846
	>12.300	2.22 (1.95, 2.54)	0.940	1.38 (1.21, 1.59)	0.960	0.74 (0.64, 0.85)	0.994	1.20 (1.00, 1.44)	0.991
∑highMWphth	<=9.770	2.29 (1.93, 2.73)		1.39 (1.20, 1.62)		0.72 (0.61, 0.85)		1.15 (0.94, 1.41)	
	>9.770 & <=13.420	2.32 (2.03, 2.66)	0.912	1.45 (1.25, 1.69)	0.645	0.77 (0.65, 0.91)	0.502	1.27 (1.03, 1.56)	0.489
	>13.420	1.93 (1.70, 2.20)	0.114	1.28 (1.12, 1.46)	0.329	0.72 (0.63, 0.82)	0.955	1.23 (1.03, 1.49)	0.591
∑allphth	<=19.780	2.30 (1.94, 2.72)		1.46 (1.25, 1.71)		0.80 (0.66, 0.96)		1.31 (1.03, 1.67)	
	>19.780 & <=30.900	2.07 (1.82, 2.37)	0.348	1.31 (1.15, 1.50)	0.230	0.71 (0.62, 0.82)	0.319	1.17 (0.98, 1.40)	0.434
	>30.900	2.14 (1.86, 2.47)	0.529	1.34 (1.16, 1.55)	0.364	0.72 (0.62, 0.83)	0.382	1.18 (0.99, 1.40)	0.449

Models were adjusted for age at measurement. * Indicates a significant overall effect of the maternal serum phthalate levels on the outcome. Diabetics (participants with glucose greater than 7), and participants who were not fasting for their blood test were removed. Values of insulin below the limit of detection (2.0) were set as LOD/ √2

Table S36. Age Adjusted Associations Between Categorized Phthalate Metabolite Levels and Creatinine (umol/L)

		14 year followup		20 year followup		22 year followup	
		Marginal Mean (95%CI)	p	Marginal Mean (95%CI)	p	Marginal Mean (95%CI)	p
MEP	<=1.750	64.04 (61.85,66.24)		80.75 (78.67,82.84)		81.85 (79.41,84.30)	
	>1.750 & <=5.714	61.32 (59.26,63.38)	0.075	79.30 (77.31,81.30)	0.306	80.86 (78.49,83.23)	0.559
	>5.714	62.65 (60.51,64.79)	0.371	81.78 (79.67,83.88)	0.481	83.74 (81.26,86.23)	0.276
MiBP	<=0.792	61.67 (59.50,63.84)		79.49 (77.41,81.57)		80.91 (78.43,83.39)	
	>0.792 & <=1.900	63.50 (61.28,65.72)	0.248	80.80 (78.61,82.99)	0.378	82.04 (79.38,84.70)	0.531
	>1.900	62.67 (60.63,64.71)	0.511	81.28 (79.32,83.23)	0.199	82.99 (80.76,85.22)	0.211
MnBP	<=1.999	61.97 (59.76,64.19)		79.77 (77.62,81.92)		81.26 (78.64,83.88)	
	>1.999 & <=4.099	64.58 (62.48,66.67)	0.093	82.11 (80.12,84.11)	0.106	83.50 (81.15,85.86)	0.199
	>4.099	61.22 (59.14,63.29)	0.625	79.65 (77.61,81.69)	0.932	81.36 (79.00,83.72)	0.953
MHBP	<=0.220	62.76 (61.33,64.18)		80.86 (79.42,82.30)		82.45 (80.76,84.13)	
	>0.220	62.12 (59.61,64.62)	0.663	79.64 (77.27,82.01)	0.368	81.02 (78.24,83.80)	0.378
MBzP	<=0.260	63.14 (61.65,64.64)		80.99 (79.50,82.47)		82.50 (80.72,84.28)	
	>0.260	61.43 (59.23,63.62)	0.204	79.60 (77.46,81.74)	0.272	81.24 (78.79,83.70)	0.402
MEHP	<=2.960	62.80 (60.66,64.93)		80.52 (78.46,82.58)		81.95 (79.58,84.31)	
	>2.960 & <=5.250	63.14 (60.99,65.30)	0.823	81.63 (79.56,83.71)	0.435	83.34 (80.89,85.79)	0.414
	>5.250	61.86 (59.72,63.99)	0.541	79.54 (77.47,81.61)	0.493	80.95 (78.44,83.47)	0.562
MECPP	<=0.785	61.54 (59.35,63.72)		79.05 (76.93,81.16)		80.38 (77.81,82.94)	
	>0.785 & <=1.241	62.48 (60.26,64.71)	0.551	80.54 (78.41,82.67)	0.312	82.06 (79.59,84.54)	0.341
	>1.241	63.63 (61.60,65.65)	0.168	81.86 (79.92,83.81)	0.046	83.45 (81.19,85.72)	0.070
MCMHP	<=1.153	61.44 (59.28,63.60)		78.16 (76.07,80.26)	*	79.20 (76.73,81.66)	*
	>1.153 & <=1.810	64.20 (62.01,66.40)	0.078	80.97 (78.90,83.04)	0.052	82.02 (79.57,84.47)	0.102
	>1.810	62.33 (60.28,64.38)	0.554	82.23 (80.26,84.19)	0.004	84.39 (82.13,86.65)	0.002
MCPP	<=0.190	62.20 (60.59,63.82)		80.34 (78.69,81.99)		81.94 (80.04,83.84)	
	>0.190	63.16 (61.23,65.09)	0.454	80.84 (79.02,82.67)	0.670	82.28 (80.12,84.44)	0.810
MINP	<=2.363	62.69 (60.57,64.81)		80.17 (78.01,82.33)		81.58 (79.09,84.06)	
	>2.363 & <=5.491	60.84 (58.62,63.05)	0.235	79.64 (77.56,81.71)	0.714	81.51 (79.08,83.95)	0.972
	>5.491	64.03 (61.97,66.08)	0.374	81.63 (79.64,83.62)	0.310	83.08 (80.68,85.48)	0.385
MCiOP	<=0.130	61.28 (59.03,63.54)		79.67 (77.52,81.81)		81.33 (78.75,83.90)	
	>0.130	63.16 (61.69,64.64)	0.171	80.97 (79.47,82.47)	0.305	82.43 (80.70,84.16)	0.473
MiDP	<=0.720	62.22 (60.86,63.57)		80.51 (79.13,81.89)		82.20 (80.59,83.81)	
	>0.720	64.57 (61.53,67.61)	0.165	80.70 (77.81,83.59)	0.902	81.62 (78.23,85.01)	0.757
∑MBP(i+n)	<=2.860	63.32 (61.11,65.52)		80.31 (78.18,82.44)		81.46 (78.89,84.02)	
	>2.860 & <=5.986	62.26 (60.15,64.38)	0.498	80.44 (78.40,82.49)	0.927	82.02 (79.59,84.44)	0.750
	>5.986	62.28 (60.17,64.39)	0.504	80.89 (78.85,82.93)	0.686	82.62 (80.27,84.97)	0.500
∑DEHP	<=7.700	63.02 (60.84,65.20)		79.28 (77.15,81.41)		80.26 (77.79,82.73)	
	>7.700 & <=11.320	62.11 (59.96,64.27)	0.561	81.80 (79.75,83.84)	0.081	84.00 (81.62,86.39)	0.029
	>11.320	62.70 (60.62,64.79)	0.836	80.37 (78.34,82.40)	0.450	81.86 (79.41,84.30)	0.356
∑DiNP	<=3.700	62.43 (60.28,64.59)		80.55 (78.39,82.71)		82.15 (79.67,84.62)	
	>3.700 & <=8.292	61.59 (59.39,63.79)	0.591	79.53 (77.46,81.60)	0.482	81.06 (78.61,83.51)	0.532
	>8.292	63.64 (61.58,65.70)	0.428	81.50 (79.51,83.48)	0.510	83.00 (80.60,85.40)	0.621
∑DEHPDiNP	<=9.529	61.63 (59.45,63.82)		80.02 (77.84,82.19)		81.67 (79.18,84.17)	
	>9.529 & <=12.850	62.76 (60.56,64.97)	0.473	80.96 (78.87,83.05)	0.521	82.55 (80.10,85.00)	0.619
	>12.850	63.28 (61.24,65.33)	0.279	80.69 (78.72,82.66)	0.641	81.99 (79.59,84.39)	0.853
∑low MWphth	<=5.550	63.57 (61.37,65.76)		80.61 (78.46,82.75)		81.81 (79.26,84.35)	
	>5.550 & <=12.300	62.55 (60.37,64.73)	0.517	79.97 (77.92,82.02)	0.664	81.31 (78.85,83.77)	0.777
	>12.300	61.83 (59.77,63.88)	0.256	81.01 (79.00,83.02)	0.780	82.98 (80.64,85.31)	0.497
∑highMWphth	<=9.770	62.08 (59.90,64.27)		80.00 (77.83,82.16)		81.50 (79.02,83.99)	
	>9.770 & <=13.420	62.12 (59.96,64.28)	0.980	80.80 (78.68,82.91)	0.587	82.58 (80.09,85.06)	0.543
	>13.420	63.49 (61.42,65.56)	0.359	80.85 (78.89,82.81)	0.552	82.16 (79.78,84.54)	0.703
∑allphth	<=19.780	62.90 (60.67,65.13)		80.71 (78.49,82.93)		82.15 (79.52,84.77)	
	>19.780 & <=30.900	62.94 (60.83,65.04)	0.982	79.72 (77.75,81.69)	0.496	80.78 (78.43,83.13)	0.436
	>30.900	61.98 (59.88,64.08)	0.556	81.36 (79.32,83.40)	0.659	83.36 (80.99,85.73)	0.494

Models were adjusted for age at measurement. * Indicates a significant overall effect of the maternal serum phthalate levels on the outcome.

Table S37. Age Adjusted Associations Between Categorized Phthalate Metabolite Levels and Triglycerides (TG, mmol/L)

		14 year followup		17 year followup		20 year followup		22 year followup	
		Geometric Marginal Mean (95%CI)	p	Geometric Marginal Mean (95%CI)	p	Geometric Marginal Mean (95%CI)	p	Geometric Marginal Mean (95%CI)	p
MEP	<=1.750	0.86 (0.78,0.94)		0.93 (0.86,1.01)		1.02 (0.93,1.11)		1.08 (0.98,1.20)	
	>1.750 & <=5.714	0.84 (0.77,0.92)	0.831	0.90 (0.83,0.97)	0.489	0.95 (0.88,1.04)	0.296	1.00 (0.90,1.10)	0.264
	>5.714	0.93 (0.85,1.02)	0.213	0.98 (0.91,1.06)	0.355	1.04 (0.95,1.13)	0.738	1.08 (0.97,1.20)	0.984
MiBP	<=0.792	0.83 (0.75,0.91)		0.89 (0.82,0.96)		0.96 (0.88,1.04)		1.01 (0.91,1.12)	
	>0.792 & <=1.900	0.92 (0.84,1.02)	0.102	0.99 (0.91,1.07)	0.067	1.06 (0.96,1.16)	0.125	1.11 (0.99,1.24)	0.228
	>1.900	0.88 (0.80,0.96)	0.344	0.94 (0.87,1.01)	0.336	1.00 (0.92,1.08)	0.463	1.05 (0.95,1.15)	0.597
MnBP	<=1.999	0.84 (0.76,0.92)		0.88 (0.82,0.96)		0.93 (0.85,1.02)	*	0.97 (0.87,1.08)	*
	>1.999 & <=4.099	0.90 (0.82,0.99)	0.287	0.99 (0.91,1.07)	**0.050**	1.09 (1.00,1.18)	**0.016**	1.16 (1.05,1.28)	**0.017**
	>4.099	0.88 (0.81,0.97)	0.465	0.93 (0.86,1.01)	0.361	0.98 (0.90,1.07)	0.384	1.02 (0.93,1.13)	0.453
MHBP	<=0.220	0.88 (0.83,0.94)		0.95 (0.90,1.00)		1.01 (0.96,1.08)		1.07 (1.00,1.14)	
	>0.220	0.85 (0.77,0.95)	0.593	0.91 (0.83,0.99)	0.421	0.96 (0.87,1.06)	0.369	1.01 (0.89,1.13)	0.397
MBzP	<=0.260	0.85 (0.80,0.91)		0.92 (0.87,0.97)		0.99 (0.93,1.05)		1.04 (0.97,1.12)	
	>0.260	0.93 (0.84,1.02)	0.147	0.98 (0.90,1.06)	0.173	1.04 (0.95,1.13)	0.356	1.08 (0.97,1.20)	0.560
MEHP	<=2.960	0.95 (0.86,1.04)		1.00 (0.93,1.08)	*	1.06 (0.98,1.15)	*	1.11 (1.00,1.22)	*
	>2.960 & <=5.250	0.88 (0.80,0.96)	0.278	0.96 (0.89,1.04)	0.497	1.06 (0.97,1.15)	0.961	1.13 (1.02,1.25)	0.763
	>5.250	0.81 (0.74,0.89)	**0.019**	0.85 (0.78,0.92)	**0.003**	0.89 (0.82,0.97)	**0.004**	0.92 (0.83,1.02)	**0.013**
MECPP	<=0.785	0.84 (0.77,0.93)	*	0.92 (0.85,0.99)		1.00 (0.91,1.09)		1.06 (0.95,1.19)	
	>0.785 & <=1.241	0.96 (0.88,1.06)	**0.047**	1.01 (0.93,1.09)	0.098	1.06 (0.97,1.15)	0.399	1.09 (0.98,1.21)	0.748
	>1.241	0.83 (0.76,0.91)	0.833	0.89 (0.83,0.96)	0.628	0.96 (0.88,1.04)	0.511	1.01 (0.92,1.11)	0.499
MCMHP	<=1.153	0.90 (0.82,0.99)		0.95 (0.88,1.03)		1.01 (0.93,1.11)		1.06 (0.95,1.18)	
	>1.153 & <=1.810	0.93 (0.84,1.02)	0.660	0.97 (0.89,1.05)	0.767	1.02 (0.93,1.11)	0.952	1.05 (0.95,1.17)	0.943
	>1.810	0.81 (0.75,0.89)	0.127	0.89 (0.82,0.96)	0.199	0.97 (0.90,1.06)	0.491	1.04 (0.94,1.14)	0.767
MCPP	<=0.190	0.88 (0.82,0.94)		0.94 (0.89,1.00)		1.01 (0.95,1.08)		1.07 (0.98,1.15)	
	>0.190	0.87 (0.80,0.94)	0.774	0.92 (0.86,0.99)	0.643	0.99 (0.91,1.06)	0.589	1.03 (0.94,1.13)	0.603
MINP	<=2.363	0.90 (0.82,0.99)		0.98 (0.90,1.06)		1.06 (0.97,1.15)		1.12 (1.01,1.24)	
	>2.363 & <=5.491	0.82 (0.75,0.90)	0.158	0.87 (0.81,0.95)	0.059	0.94 (0.86,1.02)	0.053	0.98 (0.89,1.09)	0.081
	>5.491	0.90 (0.82,0.99)	0.993	0.96 (0.89,1.03)	0.739	1.02 (0.94,1.11)	0.549	1.06 (0.96,1.18)	0.499
MCiOP	<=0.130	0.91 (0.83,1.01)		0.97 (0.90,1.06)		1.04 (0.95,1.14)		1.09 (0.98,1.22)	
	>0.130	0.86 (0.81,0.92)	0.309	0.92 (0.87,0.97)	0.242	0.98 (0.93,1.04)	0.306	1.03 (0.96,1.11)	0.408
MiDP	<=0.720	0.85 (0.81, 0.9)		0.92 (0.88, 0.97)		1.00 (0.94, 1.05)		1.05 (0.99, 1.12)	
	>0.720	0.99 (0.87, 1.13)	**0.042**	1.01 (0.90, 1.14)	0.128	1.04 (0.92, 1.18)	0.547	1.06 (0.91, 1.22)	0.966
∑MBP(i+n)	<=2.860	0.83 (0.75,0.91)		0.88 (0.81,0.95)		0.94 (0.86,1.02)		0.98 (0.88,1.09)	
	>2.860 & <=5.986	0.92 (0.84,1.01)	0.114	1.00 (0.92,1.08)	**0.026**	1.08 (1.00,1.18)	**0.020**	1.15 (1.04,1.28)	**0.034**
	>5.986	0.88 (0.80,0.97)	0.361	0.93 (0.86,1.01)	0.325	0.99 (0.91,1.07)	0.421	1.03 (0.93,1.13)	0.537
∑DEHP	<=7.700	0.93 (0.85,1.02)	*	1.00 (0.92,1.08)	*	1.08 (0.99,1.18)		1.14 (1.02,1.26)	
	>7.700 & <=11.320	0.91 (0.83,1.00)	0.812	0.95 (0.88,1.03)	0.367	0.99 (0.91,1.08)	0.161	1.02 (0.92,1.12)	0.129
	>11.320	0.79 (0.72,0.87)	**0.015**	0.86 (0.80,0.93)	**0.011**	0.95 (0.87,1.03)	**0.042**	1.01 (0.91,1.12)	0.128
∑DiNP	<=3.700	0.89 (0.81,0.98)		0.96 (0.89,1.04)		1.03 (0.95,1.13)		1.09 (0.98,1.21)	
	>3.700 & <=8.292	0.83 (0.76,0.92)	0.307	0.89 (0.82,0.96)	0.199	0.96 (0.88,1.04)	0.212	1.00 (0.91,1.11)	0.275
	>8.292	0.90 (0.82,0.98)	0.909	0.96 (0.88,1.03)	0.932	1.02 (0.93,1.11)	0.778	1.06 (0.96,1.18)	0.721
∑DEHPDiNP	<=9.529	0.91 (0.83,1.00)		0.97 (0.89,1.05)	*	1.03 (0.94,1.12)	*	1.07 (0.97,1.19)	*
	>9.529 & <=12.850	0.91 (0.83,1.00)	0.982	0.99 (0.92,1.07)	0.688	1.08 (0.99,1.18)	0.440	1.15 (1.03,1.27)	0.376
	>12.850	0.81 (0.74,0.88)	0.062	0.86 (0.80,0.93)	**0.031**	0.91 (0.84,0.99)	0.054	0.95 (0.86,1.05)	0.112
∑low MWphth	<=5.550	0.84 (0.76,0.92)		0.90 (0.83,0.97)		0.96 (0.88,1.05)		1.00 (0.90,1.12)	
	>5.550 & <=12.300	0.88 (0.80,0.97)	0.483	0.95 (0.88,1.03)	0.268	1.04 (0.95,1.13)	0.208	1.10 (0.99,1.22)	0.232
	>12.300	0.90 (0.82,0.99)	0.280	0.95 (0.88,1.03)	0.272	1.01 (0.93,1.10)	0.402	1.05 (0.95,1.16)	0.542
∑highMWphth	<=9.770	0.92 (0.83,1.01)		0.97 (0.90,1.05)	*	1.03 (0.94,1.13)	*	1.07 (0.97,1.19)	
	>9.770 & <=13.420	0.90 (0.82,0.99)	0.839	0.98 (0.91,1.06)	0.843	1.07 (0.98,1.17)	0.556	1.14 (1.02,1.26)	0.462
	>13.420	0.81 (0.74,0.89)	0.070	0.86 (0.80,0.93)	**0.039**	0.92 (0.85,1.00)	0.068	0.96 (0.87,1.06)	0.137
∑allphth	<=19.780	0.87 (0.79,0.96)		0.93 (0.86,1.01)		1.00 (0.91,1.09)		1.05 (0.94,1.17)	
	>19.780 & <=30.900	0.87 (0.79,0.95)	0.936	0.95 (0.88,1.02)	0.777	1.04 (0.95,1.13)	0.543	1.10 (1.00,1.22)	0.475
	>30.900	0.89 (0.81,0.97)	0.801	0.93 (0.86,1.00)	0.933	0.97 (0.89,1.06)	0.670	1.00 (0.91,1.11)	0.573

Models were adjusted for age at measurement. * Indicates a significant overall effect of the maternal serum phthalate levels on the outcome. Participants who were not fasting for their blood test were removed.

Table S38. Age Adjusted Associations Between Categorized Phthalate Metabolite Levels and Fat Mass (g)

		27 year followup		Change from 20-27	
		Marginal Geometric Mean (95%CI)	p	Marginal Geometric Mean (95%CI)	p
MEP	<=1.750	19895 (17664,22408)		3823 (3574,4075)	
	>1.750 & <=5.714	21746 (19413,24359)	0.289	5102 (4670,5568)	0.495
	>5.714	20119 (17884,22632)	0.896	4641 (4216,5106)	0.544
MiBP	<=0.792	19469 (17334,21866)		3843 (3545,4159)	
	>0.792 & <=1.900	21046 (18660,23738)	0.361	4029 (3727,4345)	0.929
	>1.900	21186 (18944,23694)	0.304	5429 (4997,5892)	0.327
MnBP	<=1.999	19906 (17701,22386)		4147 (3848,4458)	
	>1.999 & <=4.099	22080 (19645,24818)	0.220	4620 (4195,5087)	0.989
	>4.099	19755 (17625,22142)	0.927	4717 (4343,5116)	0.621
MHBP	<=0.220	20827 (19263,22517)		4324 (4095,4561)	
	>0.220	19923 (17433,22770)	0.574	4953 (4418,5549)	0.482
MBzP	<=0.260	20690 (19093,22421)		4325 (4072,4591)	
	>0.260	20351 (17986,23027)	0.826	4981 (4571,5416)	0.534
MEHP	<=2.960	21631 (19276,24274)		5215 (4818,5634)	
	>2.960 & <=5.250	20108 (17830,22677)	0.390	5073 (4542,5666)	0.855
	>5.250	20100 (17929,22534)	0.375	3268 (3062,3477)	0.218
MECPP	<=0.785	20636 (18300,23271)		3036 (2829,3249)	
	>0.785 & <=1.241	21792 (19242,24681)	0.537	6484 (5792,7256)	0.021
	>1.241	19757 (17749,21991)	0.596	4223 (3919,4544)	0.292
MCMHP	<=1.153	20085 (17831,22625)		3344 (3124,3567)	
	>1.153 & <=1.810	22475 (20008,25247)	0.186	6457 (5899,7061)	0.056
	>1.810	19402 (17387,21649)	0.675	3791 (3553,4035)	0.657
MCPP	<=0.190	20938 (19121,22928)		3817 (3578,4068)	
	>0.190	20117 (18192,22246)	0.563	5223 (4810,5668)	0.130
MINP	<=2.363	20306 (17874,23069)		4417 (3937,4954)	
	>2.363 & <=5.491	20388 (18195,22846)	0.963	2826 (2725,2908)	0.258
	>5.491	20976 (18792,23414)	0.706	5865 (5379,6391)	0.315
MCiOP	<=0.130	20515 (18165,23168)		4671 (4273,5099)	
	>0.130	20610 (19007,22348)	0.815	4423 (4170,4690)	0.950
MiDP	<=0.720	20098 (18662,21644)		4637 (4363,4928)	
	>0.720	23097 (19745,27018)	0.116	3182 (3155,3111)	0.196
∑MBP(i+n)	<=2.860	19929 (17784,22333)		3661 (3466,3848)	
	>2.860 & <=5.986	21998 (19471,24854)	0.342	5399 (4809,6060)	0.246
	>5.986	20055 (17908,22459)	0.433	4654 (4324,4999)	0.939
∑DEHP	<=7.700	22345 (19762,25266)		5102 (4670,5566)	
	>7.700 & <=11.320	19849 (17758,22187)	0.161	4857 (4450,5297)	0.794
	>11.320	19863 (17712,22276)	0.170	3571 (3315,3837)	0.467
∑DiNP	<=3.700	19939 (17583,22611)		4444 (4000,4934)	
	>3.700 & <=8.292	20966 (18713,23491)	0.561	3354 (3181,3519)	0.360
	>8.292	20727 (18547,23163)	0.651	5391 (4937,5882)	0.549
∑DEHPDiNP	<=9.529	20876 (18463,23605)		4585 (4180,5023)	
	>9.529 & <=12.850	20831 (18466,23500)	0.980	4572 (4208,4958)	0.998
	>12.850	20144 (18074,22452)	0.669	4374 (4032,4740)	0.968
∑low MWphth	<=5.550	19524 (17275,22065)		3613 (3370,3859)	
	>5.550 & <=12.300	22191 (19805,24865)	0.133	6259 (5703,6866)	0.114
	>12.300	20045 (17925,22415)	0.756	3675 (3440,3916)	0.979
∑highMWphth	<=9.770	21118 (18704,23843)		5139 (4691,5622)	
	>9.770 & <=13.420	20465 (18086,23157)	0.723	4041 (3709,4395)	0.497
	>13.420	20252 (18196,22541)	0.612	4358 (4034,4703)	0.644
∑allphth	<=19.780	20666 (18182,23489)		4041 (3730,4365)	
	>19.780 & <=30.900	20943 (18665,23500)	0.472	5072 (4554,5648)	0.879
	>30.900	20223 (18118,22572)	0.804	4286 (4018,4560)	0.801

Models were adjusted for age at measurement.

Table S39. Age Adjusted Associations Between Categorized Phthalate Metabolite Levels and Lean Mass (g)

		27 year followup		Change from 20-27	
		Marginal Mean (95%CI)	p	Marginal Mean (95%CI)	p
MEP	<=1.750	58669 (56579, 60759)		2212 (2193, 2231)	
	>1.750 & <=5.714	60003 (58070, 61935)	0.359	2455 (2410, 2500)	0.756
	>5.714	58131 (56138, 60124)	0.715	658 (600, 716)	0.054
MiBP	<=0.792	58788 (56764, 60811)		2191 (2114, 2269)	
	>0.792 & <=1.900	59512 (57423, 61600)	0.626	1399 (1324, 1474)	0.349
	>1.900	58723 (56753, 60694)	0.964	1798 (1733, 1864)	0.617
MnBP	<=1.999	59203 (57154, 61253)		2122 (2081, 2163)	
	>1.999 & <=4.099	59736 (57750, 61722)	0.715	2170 (2089, 2252)	0.954
	>4.099	58100 (56096, 60103)	0.450	1253 (1203, 1303)	0.279
MHBP	<=0.220	59088 (57729, 60447)		2002 (1959, 2045)	
	>0.220	58714 (56422, 61007)	0.784	1256 (1154, 1357)	0.332
MBzP	<=0.260	59262 (57870, 60655)		1917 (1870, 1965)	
	>0.260	58385 (56248, 60523)	0.500	1558 (1508, 1607)	0.637
MEHP	<=2.960	59004 (56987, 61021)		100 (26, 175)	*
	>2.960 & <=5.250	58772 (56715, 60828)	0.875	2210 (2084, 2336)	0.008
	>5.250	59139 (57130, 61148)	0.926	2902 (2819, 2985)	<.001
MECPP	<=0.785	58232 (56147, 60316)		1811 (1747, 1875)	
	>0.785 & <=1.241	59075 (56950, 61200)	0.579	1634 (1542, 1726)	0.840
	>1.241	59560 (57683, 61437)	0.354	1957 (1909, 2005)	0.854
MCMHP	<=1.153	58564 (56484, 60643)		1781 (1713, 1850)	
	>1.153 & <=1.810	58052 (56017, 60087)	0.730	1313 (1248, 1378)	0.577
	>1.810	60228 (58302, 62154)	0.250	2296 (2242, 2350)	0.528
MCPP	<=0.190	58688 (57120, 60257)		1737 (1687, 1787)	
	>0.190	59394 (57647, 61140)	0.556	1921 (1864, 1977)	0.784
MINP	<=2.363	57699 (55563, 59835)		499 (374, 624)	*
	>2.363 & <=5.491	59050 (57050, 61050)	0.366	2672 (2630, 2714)	0.013
	>5.491	59799 (57871, 61727)	0.153	1812 (1749, 1875)	0.122
MCiOP	<=0.130	55699 (53658, 57741)		1304 (1253, 1355)	
	>0.130	60476 (59114, 61838)	<.001	2039 (2001, 2076)	0.313
MiDP	<=0.720	58872 (57590, 60154)		1761 (1713, 1809)	
	>0.720	59610 (56814, 62405)	0.639	2096 (2106, 2085)	0.709
∑MBP(i+n)	<=2.860	59964 (57965, 61964)		2157 (2129, 2186)	
	>2.860 & <=5.986	59021 (56976, 61067)	0.518	2402 (2288, 2516)	0.771
	>5.986	58142 (56145, 60140)	0.207	1015 (972, 1059)	0.145
∑DEHP	<=7.700	57860 (55741, 59979)		985 (919, 1051)	*
	>7.700 & <=11.320	58604 (56664, 60543)	0.612	1264 (1197, 1331)	0.733
	>11.320	60394 (58401, 62386)	0.088	3063 (3000, 3126)	0.013
∑DiNP	<=3.700	57132 (55014, 59250)		329 (233, 425)	*
	>3.700 & <=8.292	59303 (57330, 61276)	0.142	2892 (2851, 2933)	0.003
	>8.292	60122 (58190, 62053)	0.041	1821 (1761, 1881)	0.070
∑DEHPDiNP	<=9.529	57015 (54930, 59101)		472 (411, 534)	*
	>9.529 & <=12.850	59347 (57284, 61409)	0.119	1934 (1892, 1977)	0.089
	>12.850	60226 (58362, 62090)	0.025	2613 (2564, 2663)	0.007
∑low MWphth	<=5.550	58990 (56827, 61153)		3052 (2979, 3125)	*
	>5.550 & <=12.300	58599 (56629, 60569)	0.793	1004 (913, 1096)	0.012
	>12.300	59242 (57285, 61199)	0.866	1388 (1309, 1466)	0.038
∑highMWphth	<=9.770	56949 (54883, 59016)		416 (361, 471)	*
	>9.770 & <=13.420	59296 (57195, 61398)	0.119	2014 (1958, 2070)	0.067
	>13.420	60326 (58479, 62173)	0.017	2578 (2533, 2623)	0.006
∑allphth	<=19.780	58889 (56662, 61117)		2793 (2724, 2861)	
	>19.780 &	58302 (56348, 60257)	0.698	1041 (932, 1151)	0.036
	>30.900	59705 (57776, 61633)	0.588	1828 (1779, 1877)	0.248

Models were adjusted for age at measurement. * Indicates a significant overall effect of the maternal serum phthalate levels on the outcome.

Table S40. Age Adjusted Associations Between Categorized Phthalate Metabolite Levels and Non-Alcoholic Fatty Liver Disease (NAFLD)

		Non-Alcoholic Fatty Liver Disease	
		OR (95% CI)	p
MEP	<=1.750	ref	
	>1.750 &	1.36 (0.43, 4.32)	0.603
	>5.714	1.56 (0.50, 4.83)	0.441
MiBP	<=0.792	ref	
	>0.792 &	1.06 (0.34, 3.30)	0.915
	>1.900	1.03 (0.34, 3.15)	0.959
MnBP	<=1.999	ref	
	>1.999 &	4.48 (1.13, 17.77)	0.033
	>4.099	3.12 (0.72, 13.46)	0.127
MHBP	<=0.220	ref	
	>0.220	4.48 (0.88, 22.94)	0.072
MBzP	<=0.260	ref	
	>0.260	3.53 (0.74, 16.91)	0.114
MEHP	<=2.960	ref	
	>2.960 &	1.57 (0.48, 5.18)	0.456
	>5.250	1.82 (0.55, 5.99)	0.325
MECPP	<=0.785	ref	
	>0.785 &	0.71 (0.21, 2.44)	0.586
	>1.241	0.82 (0.28, 2.38)	0.714
MCMHP	<=1.153	ref	
	>1.153 &	1.03 (0.33, 3.20)	0.957
	>1.810	0.69 (0.22, 2.11)	0.514
MCPP	<=0.190	ref	
	>0.190	3.47 (0.70, 17.17)	0.127
MINP	<=2.363	ref	
	>2.363 &	1.80 (0.57, 5.68)	0.318
	>5.491	0.97 (0.27, 3.40)	0.958
MCiOP	<=0.130	ref	
	>0.130	3.56 (0.73, 17.31)	0.116
MiDP	<=0.720	ref	
	>0.720	3.48 (0.71, 17.01)	0.123
∑MBP(i+n)	<=2.860	ref	
	>2.860 &	1.30 (0.40, 4.21)	0.659
	>5.986	1.62 (0.51, 5.13)	0.415
∑DEHP	<=7.700	ref	
	>7.700 &	1.05 (0.30, 3.69)	0.945
	>11.320	1.74 (0.55, 5.56)	0.347
∑DiNP	<=3.700	ref	
	>3.700 &	3.23 (0.84, 12.39)	0.087
	>8.292	1.92 (0.47, 7.92)	0.366
∑DEHPDiNP	<=9.529	ref	
	>9.529 &	1.90 (0.46, 7.81)	0.375
	>12.850	2.72 (0.71, 10.39)	0.143
∑low MWphth	<=5.550	ref	
	>5.550 &	2.56 (0.74, 8.91)	0.139
	>12.300	2.41 (0.68, 8.60)	0.175
∑highMWphth	<=9.770	ref	
	>9.770 &	2.33 (0.58, 9.35)	0.235
	>13.420	2.32 (0.60, 8.95)	0.221
∑allphth	<=19.780	ref	
	>19.780 &	1.00 (0.30, 3.37)	1.000
	>30.900	1.62 (0.50, 5.25)	0.418

Models were adjusted for age at measurement.
